# Engineering Metal Pyrazolate Coordination Polymers for Highly Selective Direct Air Capture via Metal–Ligand Cooperativity and Moisture-Gated Mechanism

**DOI:** 10.1021/jacs.6c15929

**Published:** 2026-10-02

**Authors:** Tsu-Chieh Yu, Chun-Kai Huang, Ru-Yun Yang, Yi-Tsung Lai, Qing-Hui Lin, Ming-Jun Han, Swatilekha Pratihar, Sue-min Chang, Yu-Chen Chung, Bo-Hao Chen, Yu-Chun Chuang, Chia-Her Lin, Ho-Hsiu Chou, Tsai-Te Lu

**Affiliations:** † Institute of Biomedical Engineering, 34881 National Tsing Hua University, Hsinchu 300044, Taiwan; ‡ Department of Chemistry, National Tsing Hua University, Hsinchu 300044, Taiwan; § Department of Chemical Engineering, National Tsing Hua University, Hsinchu 300044, Taiwan; ∥ Institute of Environmental Engineering, 34914 National Yang Ming Chiao Tung University, Hsinchu 300093, Taiwan; ⊥ 57815 National Synchrotron Radiation Research Center, Hsinchu 30076, Taiwan; # Department of Chemistry, Chung Yuan Christian University, Taoyuan 320314, Taiwan

## Abstract

Direct air capture (DAC) of CO_2_ has emerged as an indispensable negative emission technology, yet current sorbents remain constrained by the persistent trade-off between intrinsic capture capacity and regeneration energy alongside the extrinsic challenge of atmospheric moisture inhibition. Herein, engineering of crystalline and thermally stable alkali metal pyrazolate coordination polymers (**M** ^**R**^ **Pyr**) is established as a novel paradigm for the metal–ligand cooperative (MLC), moisture-gated, and highly selective capture of CO_2_ from ambient air streams. While inert in low-humidity air, activation of solid-state **M** ^**R**^ **Pyr** by atmospheric moisture unlocks exceptional CO_2_-capture capacities (7.4–9.2 mmol/g) and realizes a substantial CO_2_:pyrazolate stoichiometry (0.83–0.89) through formation of metal carbamate (**M** ^**R**^ **Carbamate**). To elucidate the operative mechanism, crystallographic snapshots and *in*/*ex situ* spectroscopic analyses unveil that MLC hydration of **K** ^**Me**^ **Pyr** yielding **K** ^**Me**^ **Pyr**·**H**_**2**_ **O** triggers a substantial structural rearrangement from a compact double-ladder chain into an expanded zig-zag architecture. This moisture-gated transformation relieves steric shielding and unleashes intrinsic nucleophilicity of pyrazolate ligands to initiate CO_2_ capture via a thermodynamically favored displacement of bound water. Finally, quantitative regeneration of **M** ^**R**^ **Pyr** from CO_2_-saturated **M** ^**R**^ **Carbamate** adducts and demonstrated multiple-cycle recyclability under a temperature-vacuum swing adsorption process position the robust **M** ^**R**^ **Pyr** platform as a paradigm-shifting class of sorbents for sustainable DAC applications.

## Graphical abstract

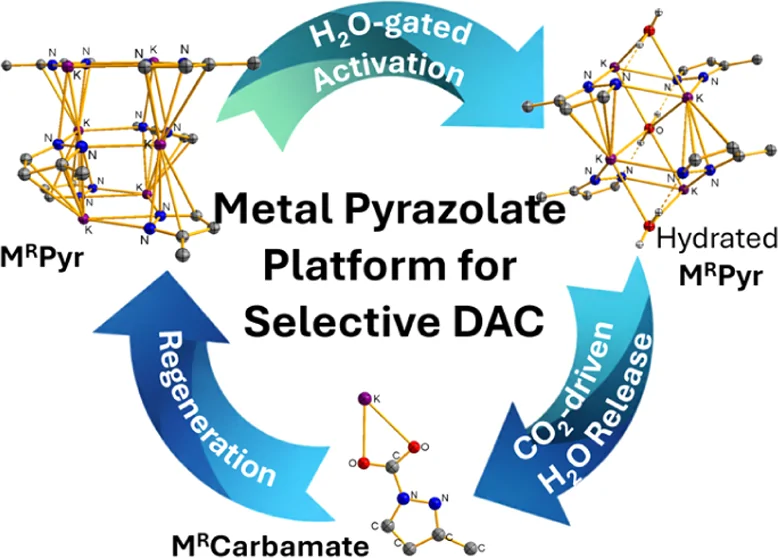

## Introduction

The escalating crisis of anthropogenic climate change, driven by atmospheric CO_2_ concentrations exceeding 422 ppm, necessitates the immediate deployment of CO_2_ neutralization technologies.^1–6^ These strategies are critical to address both the current high atmospheric CO_2_ levels and ongoing emissions stemming from industrial processes and fossil fuel combustion. While point-source capture effectively targets concentrated CO_2_ streams from industrial flues, the Intergovernmental Panel on Climate Change (IPCC) has identified negative emission technologies, specifically direct air capture (DAC), as indispensable for remediating legacy emissions and meeting the 1.5 °C climate stabilization goal.^6–17^ Owing to the geographic flexibility and land efficiency of DAC, the International Energy Agency (IEA) has projected an expansion in annual capture capacity from ∼0.02 Mt CO_2_ in 2024 to ∼980 Mt CO_2_ by 2050.^18^ To meet this goal, the advancement of DAC technologies hinges upon engineered sorbents capable of simultaneously effecting efficient CO_2_ capture from ultra-dilute sources (∼400 ppm), sustaining robust cyclability under temperature-/vacuum-swing adsorption processes, and preserving exquisite selectivity against a backdrop of competitive atmospheric moisture.^7–10,19–24^ Consequently, the efficacy of DAC is intrinsically governed by the molecular design of sorbents that can reconcile the conflicting demands of high CO_2_-capture capacity, thermodynamic stability, and kinetic selectivity under ambient conditions.^25–27^

Despite the urgent need for scalable DAC deployment, state-of-the-art adsorbent technologies remain constrained by the persistent trade-off between intrinsic CO_2_-capture capacity and regeneration energetics alongside the extrinsic challenge of atmospheric moisture inhibition (Table 1).^19,28,29^ Inorganic sorbents, such as alkaline earth metal oxides and hydroxides, offer distinct advantages in terms of chemical stability, low cost, and widespread availability.^30–36^ However, these sorbents suffer from a severe energetic penalty, as the strong metal–oxygen bonds in the metal (bi)­carbonates formed during chemisorption impose prohibitively high regeneration temperatures (typically ≥700 °C).^19,30–32^ While the recent development of carbon-supported hydroxides (porous carbon sorbent-hydroxide, PCS–OH) in combination with an electrically driven Joule heating process has successfully lowered the regeneration temperature to 90 °C, PCS–OH bears a substantial trade-off in CO_2_-capture capacity (0.2 mmol-CO_2_/g-sorbent).^36^ In comparison, molecular amines (such as monoethanolamine, MEA) afford milder regeneration conditions owing to the thermodynamic lability of the resulting carbamate N–C bonds, yet such systems are hindered by intrinsic volatility and oxidative degradation.^26,37–39^ To mitigate these stability and durability issues, the prevailing strategy has been to construct self-supported solid amine networks or incorporate amines into (porous) solid supports (e.g., resins, mesoporous silica, and metal–organic frameworks (MOFs)).^28,37,40–58^ Although effective in ameliorating the issues of volatility and oxidative degradation, the amine immobilization approach introduces a fundamental limitation regarding gravimetric efficiency. That is, the inert support constitutes a non-adsorbing parasitic mass that dilutes active site density and caps the gravimetric CO_2_-capture capacity (typically ≤4.2 mmol-CO_2_/g-sorbent, Table 1).^28,37,38,42,43,45–54^ Recently, through a hydrogen-bonding-mediated supramolecular assembly, a crystalline solid amine TriH has been successfully engineered to achieve a record-breaking capacity of 8.9 mmol-CO_2_/g-sorbent while preserving a mild regeneration at 100 °C.^44^

**Table 1:** Selected Parameters of Solid-State Sorbents for DAC of CO_2_

sorbent type	sorbent name	CO_2_-capture capacity (mmol-CO_2_/g-sorbent)^a^	CO_2_/active site molar ratio^a^	–Δ*H*_ads_ (kJ/mol-sorbent)	regeneration		ref
					temperature	energy Δ*H*_reg_ (kJ/mol-CO_2_)	
alkaline metal (hydr)oxides	CaO	3.6	0.20	171.2	850 °C (N_2_ purge)^b^	-	^31,32^
	NaOH	2.3 (25 °C)	0.09	131.5	700 °C (steam)	171.5^d^	^30^
hydroxide-based (porous) materials	PCS–OH^c^	0.2 (30 °C)	0.17	137	100 °C (Joule heating)	286	^36^
	[Zn(ZnOH)_4_(bibta)_3_]^c^	2.2 (27 °C)	0.57	71^e^	100 °C (N_2_ purge)	-	^68^
	MOF-Ni–OH	2.7 (27 °C)	0.73	84	150 °C (N_2_ purge)	-	^69^
	MOF-Co–OH	1.6 (27 °C)	0.43	68	100 °C (N_2_ purge)	-	
	Rb_2_CO_3_ CD-MOF ST^c^	0.7 (25 °C)	0.54	64	80 °C (dry N_2_ purge)	109^e^	^70^
guanidine-based platform	AA/PyBIG^c^	3.1 (21 °C)	0.76	70.7	120 °C	223	^22^
	AA/m-BBIG^c^	3.3 (25 °C)	0.80	69	120 °C	265	^71^
self-supported solid amines	TriH^c^	8.9 (20 °C)	0.49	74	100 °C (N_2_ purge)	-	^44^
	COF-999	2.05 (25 °C)	0.33	53	60 °C (N_2_ purge)	-	^45^
	TEPA-MXDA^c^	1.41 (RT)	0.14	-	80 °C	-	^46^
supported solid amine sorbents	D-HMS-T-50^c^	4.20 (25 °C)	0.33	71	100° C (N_2_ purge)	80	^72^
	[Mg_2_(dobdc)(N_2_H_4_)_1.8_]^c^	3.89 (25 °C)	0.32	90	130° C (N_2_ purge)	133	^47^
	en-Mg_2_(dobpdc)^c^	2.83 (25 °C)	0.32	50	150 °C (air purge)	-	^48^
	TEPA-silica^c^	2.50 (35 °C)	0.25	-	125 °C	-	^49^
	HP20/PEI-50^c^	2.26 (25 °C)	0.20	-	100 °C (N_2_ purge)	-	^50^
	mmen-Mg_2_(dobpdc)^c^	2.00 (25 °C)	0.29	71	150° C (N_2_ purge)	103	^43^
	FS-PEI-33^c^	1.74 (25 °C)	0.23	-	85 °C (65 mTorr)	-	^37^
	SBA-15/DEA/PEI	1.62 (25 °C)	0.20	-	70 °C (N_2_ purge)	-	^52^
	L-PPI/SBA-15^c^	1.25 (35 °C)	0.17	105	110 °C (N_2_ purge)	-	^51^
	MOF-808-lys^c^	1.21 (25 °C)	0.61	-	140 °C (Ar purge)	-	^42^
	PEI-SBA-15^c^	0.65 (30 °C)	0.10	78	110 °C (He purge)	-	^53,54^
physical adsorbents (porous materials)	Mg(dobdc)^c^	1.35 (RT)	-	47	110 °C (Ar purge)	-	^55^
	SIFSIX-3-Cu	1.24 (25 °C)	-	54	50° C (N_2_ purge)	-	^56^
	sodium mordenite	1.15 (30 °C)	-	30	100 °C (Ar/He purge)	-	^57^
	zeolite 13X	0.21 (25 °C)	-	42	150 °C (N_2_ purge)	-	^58^
metal pyrazolate	**NaPyr**	9.2 ± 0.3 (RT)	0.83 ± 0.03	-	200 ^°^C (vacuum)	49^f^	this work
	**KPyr**	8.3 ± 0.1 (RT)	0.88 ± 0.01	-	-	74^f^	this work
	**K** ^**Me**^ **Pyr**	7.4 ± 0.3 (RT)	0.89 ± 0.04	78.9	200 ^°^C (vacuum)	73^f^	this work
	**K** ^**Me**^ **Pyr**·**H**_**2**_ **O**	5.8 ± 0.5 (RT)	0.70 ± 0.08	-	-	-	this work

a Evaluated under dynamic DAC conditions, with the operating temperatures provided in the brackets.

b After capture of CO_2_ from high-concentration sources.

c PCS–OH = porous carbon sorbent-hydroxide; bibta = 5,5′-bibenzotriazolate; CD-MOF = hydroxide-based cyclodextrin MOF; ST = solvothermal; PyBIG = 2,6-pyridinebis­(iminoguanidine); *m*-BBIG = meta-benzene-bis­(iminoguanidine); AA = amino acids such as glycine and sarcosine; TriH = 1,3,5-tris­(aminomethyl)­benzene; TEPA-MXDA = tetraethylenepentamine (TEPA) linked with *N*,*N*,*N′*,*N′*-tetrakis (2,3-epoxypropyl)-*m*-xylene-alpha,alpha′-diamine; HMS = hierarchical mesoporous silica; D = 3-[2-(2-aminoethylamino)­ethylamino]­propyl-trimethoxysilane (DT); T = tetraethylenepentamine (TEPA); dobdc = 2,5-dioxido-1,4-benzenedicarboxylate; dobpdc = 4,4′-dioxido-3,3′-biphenyldicarboxylate; mmen = *N*,*N*′-dimethylethylenediamine; en = ethylenediamine; HP20 = diaion HP20 resin; L-PPI = linear poly­(propylenimine); FS-PEI-33 = fumed silica-polyethylenimine; MOF-808-Lys = Lys-functionalized MOF-808.

d Standard enthalpy of regeneration.

e Based on DFT calculation.

f The regeneration energy was directly quantified in kJ/mol-sorbent via DSC analysis and subsequently converted to kJ/mol-CO_2_ by dividing the raw values by the measured CO_2_:active site molar ratio for each metal pyrazolate coordination polymer.

Beyond these capacity–energy trade-offs, an even more critical challenge lies in circumventing the deactivation of active sites in the DAC sorbents by atmospheric moisture under real-world conditions. For the majority of DAC sorbents ranging from alkaline metal (hydr)­oxides to (self-)­supported solid amines (Table 1), the limited selectivity of active sites for CO_2_ versus atmospheric H_2_O renders moisture a potent competitive inhibitor, thereby suppressing site utilization efficiency to typically ≤0.33.^24,30–32,36,37,42,43,45–54,59–61^ Overcoming the stoichiometric limit of conventional amine-based DAC sorbents, under wet conditions, the pioneering Lys-functionalized MOF-808 (MOF-808-Lys) redirects the CO_2_-capture mechanism from carbamate formation to water-enhanced bicarbonate generation, thereby achieving an enhanced site utilization efficiency of ∼0.61 yet limited to a moderate CO_2_-capture capacity of 1.21 mmol-CO_2_/g-sorbent (Table 1).^42^ Consequently, the rational design of a novel class of solid-state adsorbents capable of simultaneously delivering (a) high active-site density devoid of inert “dead weight,” (b) low/moderate regeneration energy, (c) exceptional selectivity for CO_2_ against humidity, and (d) sustained site utilization efficiency under realistic ambient conditions represents a formidable challenge essential for a paradigm shift in the field.

Recently, a diversity of metal pyrazolate complexes ([M_*x*_(^R^Pyr)_*y*_(L)_*z*_]^*n*−^, M = Mg^2+^, Al^3+^, Ga^3+^, Sc^3+^, Ti^3+/4+^, Fe^2+^, Ce^3+/4+^; R = H, Me, ^*i*^Pr, ^*t*^Bu, CF_3_; L = NO) has been established to effect the stoichiometric capture of CO_2_ from pure gas streams.^62–67^ These CO_2_-capture processes invariably proceed via a metal–ligand cooperative (MLC) mechanism yielding stable metal carbamates. Specifically, the reaction of an iron carbamate complex [(NO)_2_Fe­(^Me^PyrCO_2_)]^−^ (**Fe** ^**Me**^ **Carbamate**, ^Me^PyrCO_2_ = 3-methylcarbamate) with a Lewis acidic Ca­(OTf)_2_ was shown to induce reduction of the captured CO_2_ as precipitated calcium oxalate.^62^ This process, moreover, regenerated the parent iron pyrazolate complex [(NO)_2_Fe­(μ-^Me^Pyr)_2_Fe­(NO)_2_]^2–^, thereby closing a synthetic cycle for the conversion of CO_2_ into C_2_ products. While these preceding works underscore the efficacy of the anionic and aromatic pyrazolate motifs for CO_2_ binding and activation, a critical electronic constraint remains. The substantial Lewis acidity of the polyvalent metal ions (M^*n*+^, *n* ≥ 2) may attenuate the nucleophilicity of the bridging pyrazolate ligands, thereby restricting their reactivity to high-concentration CO_2_ environments. Consequently, the utilization of monocationic alkali metals (e.g. Na^+^, K^+^) offers a rational strategy to unleash the nucleophilic potency of the pyrazolate moiety for DAC. However, the realization of such alkali metal-based systems capable of efficient and selective DAC remains elusive.

In this study, this design principle is realized through the development of a series of crystalline and thermally stable alkali metal pyrazolate coordination polymers [Metal]­[^R^Pyr] (**M** ^**R**^ **Pyr**, Metal = Na^+^, K^+^; R = H, Me) that exhibit a serendipitous “moisture-gated” mechanism for the capture of CO_2_ from ambient air streams. Moreover, crystallographic and spectroscopic investigations reveal that atmospheric moisture triggers a hydration-induced structural rearrangement in the compact **M** ^**R**^ **Pyr** lattice and initiates the selective capture of CO_2_ from air by driving the concomitant release of bound water. This exceptional thermodynamic selectivity for CO_2_ over H_2_O is evidenced by the stoichiometric formation of metal carbamates, achieving a substantial CO_2_/pyrazolate stoichiometry of 0.83–0.89 that far exceeds those of conventional DAC sorbents ranging from metal (hydr)­oxides to supported amines. Finally, the integration of this novel type of DAC sorbent with a temperature-vacuum swing adsorption (TVSA) process establishes the **M** ^**R**^ **Pyr** system as a recyclable and high-atom-economy platform for the selective extraction of CO_2_ from ambient air.

## Results and Discussion

### Synthesis and Characterization of Metal Pyrazolate Coordination Polymer (**M** ^**R**^ **Pyr**)

As shown in Scheme 1a, reaction of 3-methylpyrazole (8.2 g, 0.1 mol) with potassium hydride (4.0 g, 0.1 mol) resulted in the production of potassium 3-methylpyrazolate (**K** ^**Me**^ **Pyr**), which was characterized by single-crystal and powder X-ray diffraction (PXRD), magic angle spinning solid-state ^13^C NMR (^13^C MAS NMR), and thermogravimetric analysis (TGA). The single-crystal X-ray structure of **K** ^**Me**^ **Pyr** is depicted in Figure 1a,b. **K** ^**Me**^ **Pyr** displayed a one-dimensional coordination polymer with a double-ladder chain structure along with the *c-*axis (Figure 1a). Along with the longitudinal direction within each single-ladder chain: (a) K(2) and K(1) ions were sandwiched by 3-methylpyrazolate ligands through [K(2)­(η^2^-^Me^Pyr)] and [K(1)­(η^5^-^Me^Pyr)] cation–π interactions, whereas (b) [K(3)­(η^4^-^Me^Pyr)] cation–π interactions facilitated the bridging interactions between K(3) ions. In the *a*–*b* plane, assembly of [K­(μ-^Me^Pyr)_2_K] units occurred to build each layer of the double-ladder chain structure. Moreover, each one-dimensional and double-ladder-like coordination polymer chain was surrounded by six identical chains in a compact and parallel manner, which completed the single-crystal structure of **K** ^**Me**^ **Pyr** (Figure 1b).

**Scheme 1:**
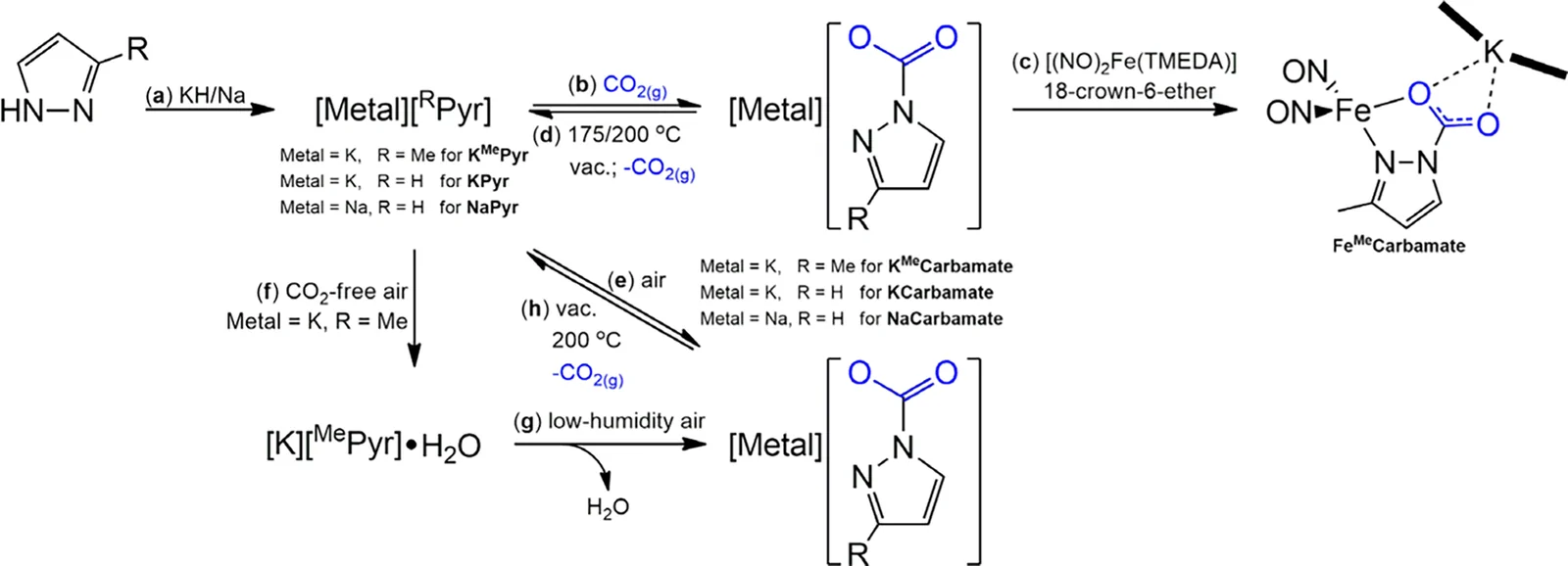
TVSA Cycle for MLC and Moisture-Gated Capture of CO_2(g)_ from Air by Metal Pyrazolate Polymers (**M** ^**R**^ **Pyr**) and Heat-/Vacuum-Induced Conversion of Metal Carbamates (**M** ^**R**^ **Carbamate**) into **M** ^**R**^ **Pyr**

**Figure 1:**
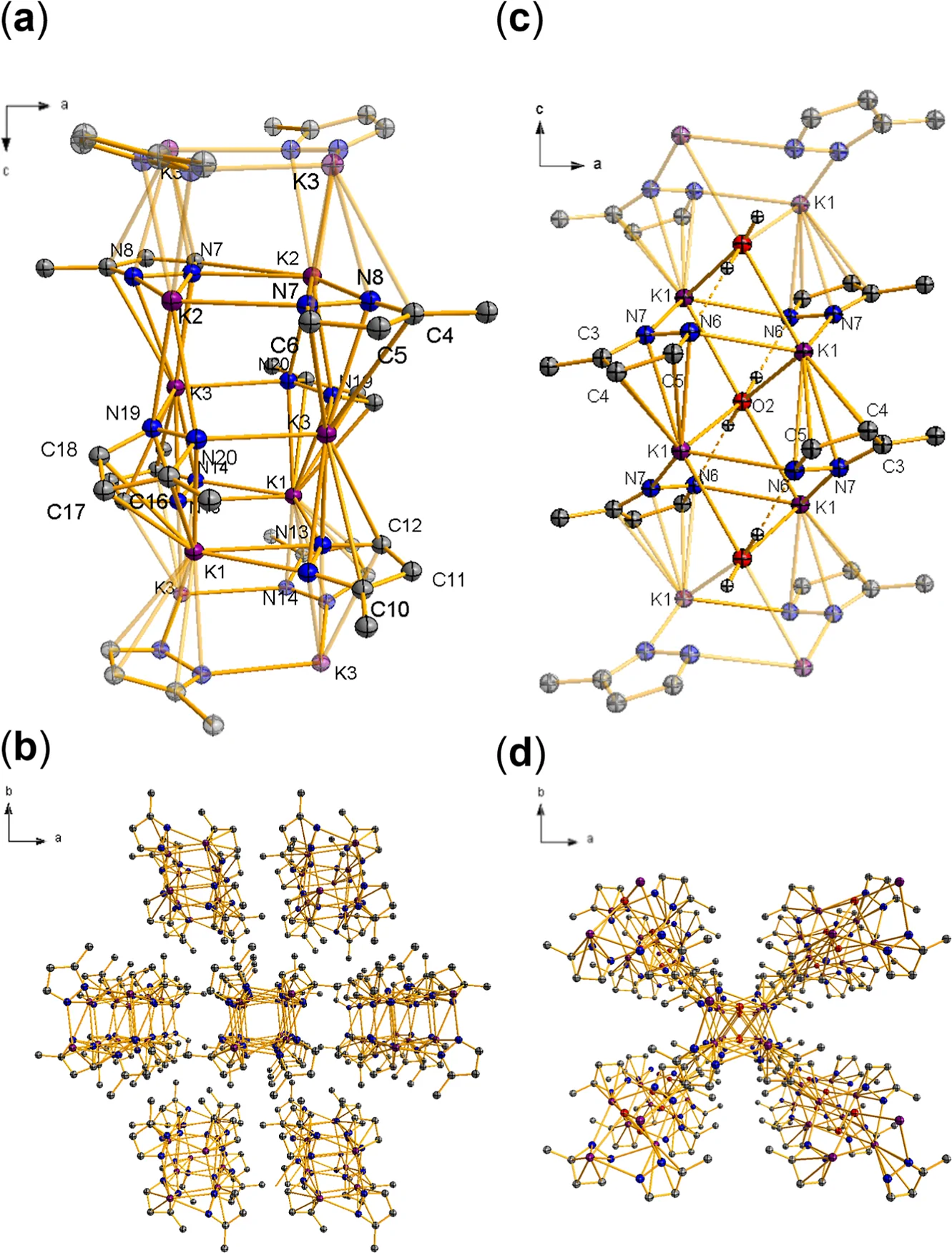
(a) One-dimensional coordination polymer and double-ladder-like chain of **K** ^**Me**^ **Pyr** with the thermal ellipsoids at the 80% probability level. (b) Packing diagram of the single-crystal structure of **K** ^**Me**^ **Pyr** showing a chain structure viewed down the *c-*axis. (c) One-dimensional coordination polymer and zig-zag architecture of **K** ^**Me**^ **Pyr**·**H**_**2**_ **O** with the thermal ellipsoids at the 80% probability level. (d) Packing diagram showing a three-dimensional structure viewed down the *c-*axis.

As shown in Figure 2a, the PXRD pattern of the as-prepared **K** ^**Me**^ **Pyr** was fully indexed to the (020), (110), (040), (200), (122), (060), and (232) facets based on the simulated pattern of single-crystal **K** ^**Me**^ **Pyr**. That is, the as-prepared **K** ^**Me**^ **Pyr** consisted of a high homogeneity of crystalline potassium 3-methylpyrazolate coordination polymer. This high homogeneity of the as-prepared **K** ^**Me**^ **Pyr** also reflected on the ^13^C MAS NMR signals of 3-methylpyrazolate linker at 14.6, 102.6, 139.9, and 147.6 ppm (Figure 2c). Furthermore, thermal stability of **K** ^**Me**^ **Pyr** was investigated by TGA. In addition to the evaporation of water (∼4%) observed before ∼150 °C, the TGA curve of **K** ^**Me**^ **Pyr** exhibited a weight loss of 29.1% at 338–350 °C, which demonstrated the thermal stability of **K** ^**Me**^ **Pyr** up to ∼330 °C (Figure 2b).

**Figure 2:**
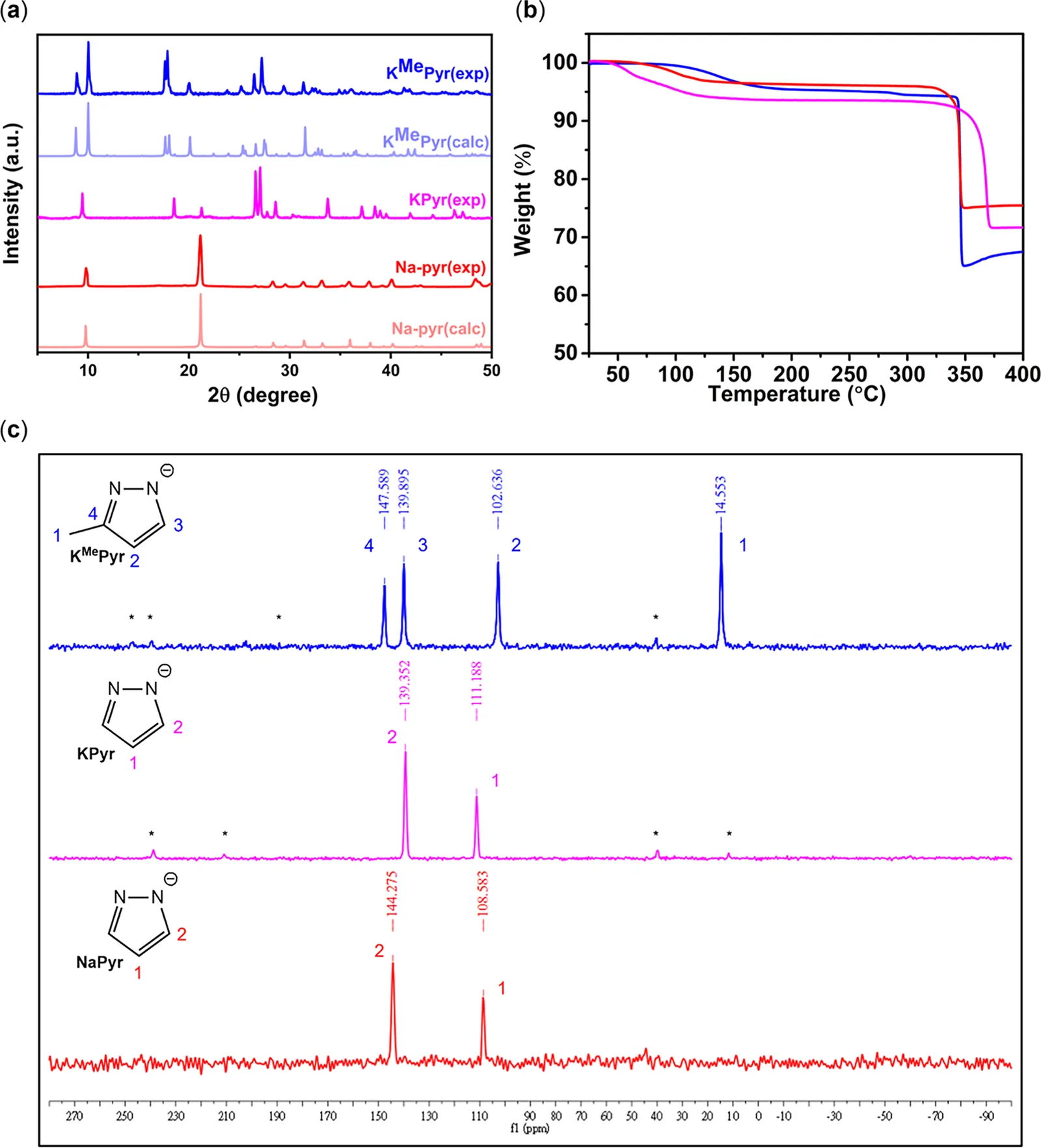
(a) PXRD patterns of the as-prepared **K** ^**Me**^ **Pyr** (blue), **KPyr** (magenta), and **NaPyr** (red). The calculated PXRD spectra of single-crystal **K** ^**Me**^ **Pyr** and **NaPyr** are depicted in light blue and light red. (b) TGA curves and (c) ^13^C MAS NMR spectra of the as-prepared **K** ^**Me**^ **Pyr** (blue), **KPyr** (magenta), and **NaPyr** (red). *: spinning side bands.

Under a similar synthetic methodology, preparations of potassium pyrazolate (**KPyr**) and sodium pyrazolate (**NaPyr**) were also performed via reactions of KH (or Na) with pyrazolate in a 1:1 stoichiometry. Based on TGA, the as-prepared **KPyr** and **NaPyr** similarly displayed thermal stability up to ∼300–310 °C, with major weight losses of 23.7% (**KPyr**) and 20.9% (**NaPyr**) observed at 310–373 °C and 310–350 °C, respectively (Figure 2b). In addition, similar to **K** ^**Me**^ **Pyr**, **NaPyr** was characterized as a coordination polymer based on the single-crystal X-ray diffraction analysis (Figure 3), whereas detailed descriptions of the single-crystal X-ray structure of **NaPyr** are discussed in the Supporting Information. Moreover, the as-prepared **NaPyr** featured a crystalline nature and high homogeneity based on the PXRD patterns and ^13^C MAS NMR signals, both of which were consistent with those of single-crystal **NaPyr** (Figure 2a,c). With the buildup of a chemical library of crystalline and thermally stable metal pyrazolate coordination polymers, **M** ^**R**^ **Pyr**, effects of the metal nodes and substituents of pyrazolate linkers on the capture of CO_2_ were further investigated.

**Figure 3:**
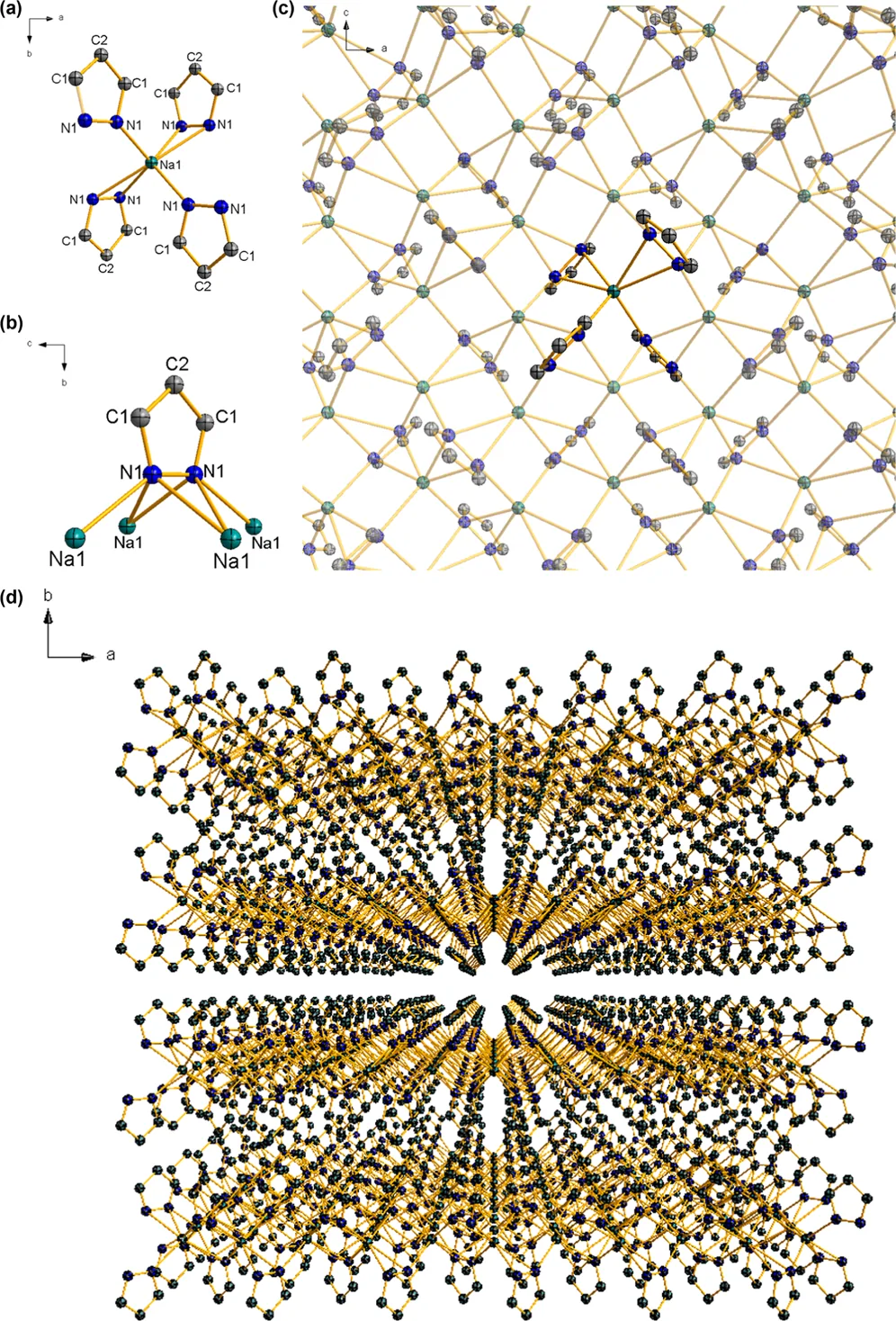
ORTEP drawing of local structures of (a) [Na­(η^1^-Pyr)_2_(η^2^-Pyr)_2_] and (b) [Na_4_(μ^4^-Pyr)] units in **NaPyr** at the 80% probability level. (c) Two-dimensional and grid-like structure of **NaPyr** with the thermal ellipsoids at the 80% probability level. (d) Packing diagram of a single-crystal structure of **NaPyr** showing a two-dimensional and grid-like structure viewed down the *c-*axis.

### Capture of CO_2_ by **M** ^**R**^ **Pyr** Yielding Metal Carbamates (**M** ^**R**^ **Carbamate**) and Heat-/Vacuum-Induced Regeneration of **M** ^**R**^ **Pyr** from **M** ^**R**^ **Carbamate**

Upon titration of the solid-state **K** ^**Me**^ **Pyr** with CO_2(g)_, gradual increase of an IR absorption peak at 1681 cm^–1^ saturating upon treatment of one equivalent of CO_2_ indicated the stoichiometric capture of CO_2_ by solid-state **K** ^**Me**^ **Pyr** via formation of potassium 3-methylcarbamate (**K** ^**Me**^ **Carbamate**, Scheme 1b and Figure 4a). Moreover, this stoichiometric capture of CO_2_ by **K** ^**Me**^ **Pyr,** leading to the assembly of **K** ^**Me**^ **Carbamate,** was investigated using IR, ^13^C MAS NMR, and PXRD. When ^13^CO_2(g)_ was used for reaction with **K** ^**Me**^ **Pyr**, the IR ν_CO_ stretching peak shifted from 1681 cm^–1^ to 1633 cm^–1^ (Figure 4b), which was relevant to the isotopic shift of the IR ν_CO_ stretching peak from 1723 cm^–1^ to 1699 cm^–1^ in the reported complex [K-18-crown-6-ether]­[(NO)_2_Fe­(^Me^PyrCO_2_)] (**Fe** ^**Me**^ **Carbamate**).^62^ Similar to the ^13^C NMR signal of Fe-bound 3-methylcarbamate in **Fe** ^**Me**^ **Carbamate** at 153.0 ppm,^62^ the ^13^C MAS NMR of captured CO_2_ in **K** ^**Me**^ **Carbamate** located at 152.1 ppm (Figure 4c). As shown in Scheme 1c and Figure S1, reaction of **K** ^**Me**^ **Carbamate** with complex [(NO)_2_Fe­(TMEDA)] (TMEDA = tetramethylethylenediamine) in the presence of 18-crown-6-ether resulted in the formation of molecular complex **Fe** ^**Me**^ **Carbamate**. This assembly of molecular complex **Fe** ^**Me**^ **Carbamate** via a ligand–exchange reaction provided additional support to the assembly of 3-methylcarbamate in **K** ^**Me**^ **Carbamate**, which was derived from the nucleophilic attack of 3-methylpyrazolate in **K** ^**Me**^ **Pyr** toward CO_2_. On the other hand, the shift in PXRD peak positions during the conversion of **K** ^**Me**^ **Pyr** into **K** ^**Me**^ **Carbamate** (Figure 4d) demonstrated that capture of CO_2_ by **K** ^**Me**^ **Pyr** promoted a solid-state phase transition into **K** ^**Me**^ **Carbamate**.

**Figure 4:**
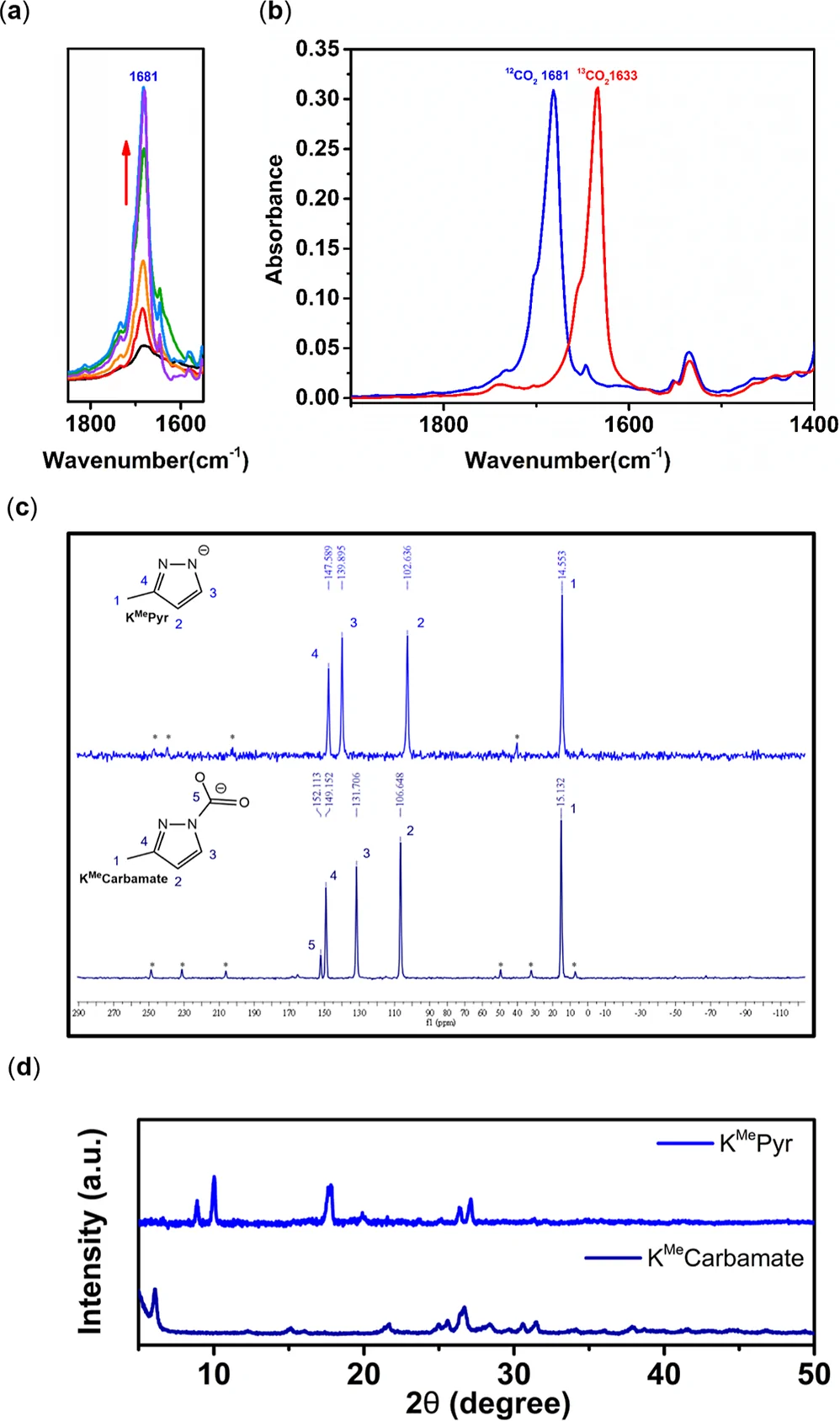
(a) Solid-state IR spectra for **K** ^**Me**^ **Pyr** (black) before and after its reaction with 0.25 (red), 0.5 (orange), 0.75 (green), 1.0 (blue), and 1.25 (purple) equiv of CO_2(g)_. (b) Solid-state IR spectra for **K** ^**Me**^ **Pyr** treated with 1.0 equiv of CO_2(g)_ (blue) and ^13^CO_2(g)_ (red). (c) ^13^C MAS NMR spectra and (d) PXRD patterns for **K** ^**Me**^ **Pyr** before (blue) and after reaction with stoichiometric CO_2(g)_ (navy). *: spinning side bands.

Data for titrations of solid-state **KPyr** (or **NaPyr**) with CO_2(g)_ and IR/^13^C MAS NMR/PXRD analyses on the obtained **KCarbamate** (or **NaCarbamate**) were collected in Figures S2,S3. Together with the investigations described above, solid-state phase transition of metal pyrazolate coordination polymers (**M** ^**R**^ **Pyr**/**K** ^**Me**^ **Pyr**/**KPyr**/**NaPyr**) into the corresponding metal carbamates (**M** ^**R**^ **Carbamate**/**K** ^**Me**^ **Carbamate**/**KCarbamate**/**NaCarbamate**) served as a MLC mechanism for efficient and stoichiometric capture of CO_2_.

Inspired by the formation of **M** ^**R**^ **Carbamate** through the capture of CO_2_ by **M** ^**R**^ **Pyr**, the poor solubility of **M** ^**R**^ **Carbamate** in most aprotic solvents, however, precluded the conventional single-crystal X-ray crystallographic study. Leveraging the high-resolution powder X-ray diffraction data of **K** ^**Me**^ **Carbamate**, its crystal structure was determined via *ab initio* structure determination from powder diffraction (SDPD; see Experimental Section 1.11 for details). The Rietveld refinement result, packing diagram, and local structures of **K** ^**Me**^ **Carbamate** are depicted in Figure 5. In the reported structure of **Fe** ^**Me**^ **Carbamate**, the monoanionic 3-methyl-pyrazole-1-carboxylate [^Me^PyrCO_2_]^−^ planar ligand chelates the K^+^ cation through an O,O-mode (K–O bond distances = 2.743 Å and 2.948 Å) and chelates the Fe center in the [Fe­(NO)_2_] unit through an O,N-mode (Fe–O bond distance = 2.079 Å; Fe–N bond distance = 2.049 Å).^62^ A slight dislocation of the K^+^ cation from the [^Me^PyrCO_2_]^−^ ligand plane was also noted. Herein, **K** ^**Me**^ **Carbamate** displays similar chelation modes of the [^Me^PyrCO_2_]^−^ ligand to K^+^ cations (Figure 5d), through (a) an O,O-chelation mode with K–O bond distances of 2.615 Å and 3.245 Å and (b) an O,N-chelation mode with K–O and K–N bond distances of 3.118 Å and 2.873 Å. In addition, twisting of the carboxylate moiety and dislocation of chelated K^+^ cations from the 3-methylpyrazolate plane occur, enabling interaction with additional K^+^ cations and [^Me^PyrCO_2_]^−^ ligands along the *a*-axis (Figure 5b). Moreover, the [^Me^PyrCO_2_]^−^ ligand also serves as an organic linker bridging four K^+^ cations along the *a*- and *b*-axes, with K–O bond distances of 2.560 Å, 2.786 Å, 2.793 Å, and 3.118 Å, respectively. As shown in Figure 5e, each K^+^ cation serves as a metal node interacting with five [^Me^PyrCO_2_]^−^ ligands through (a) η^1^-O, (b) η^2^-O,O, and (c) η^2^-O,N binding modes. Taken together, these K^+^-[^Me^PyrCO_2_]^−^ interactions result in the assembly of **K** ^**Me**^ **Carbamate** as a two-dimensional coordination polymer extending along the *a*- and *b*-axes (Figure 5b,e). Furthermore, stacking of these two-dimensional sheets along the *c*-axis completed the structure of **K** ^**Me**^ **Carbamate**.

**Figure 5:**
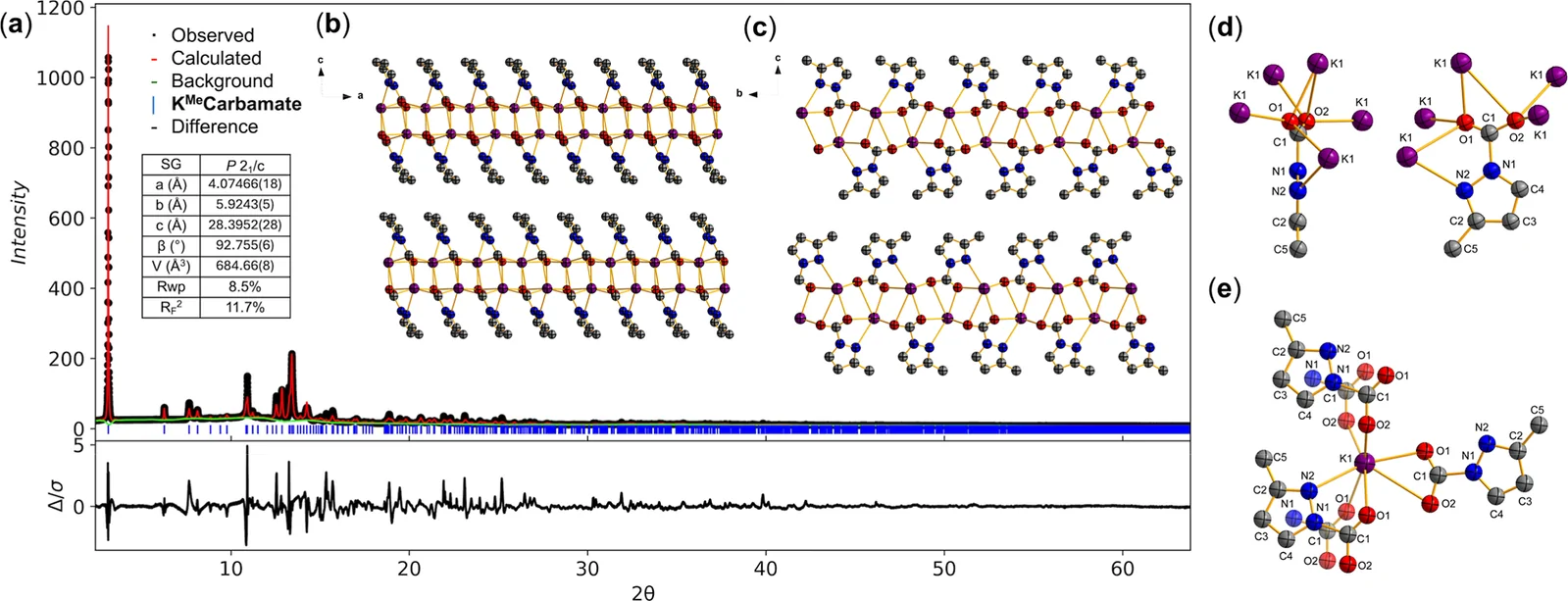
(a) Rietveld refinement result of **K** ^**Me**^ **Carbamate**. (b,c) Packing diagrams of **K** ^**Me**^ **Carbamate** viewed along the (b) *a*-axis and (c) *b*-axis. (d,e) ORTEP drawing of local structures of (d) [K_5_(μ^5^-^Me^PyrCO_2_)] and (e) [K­(η^1^-O-^Me^PyrCO_2_)_3_(η^2^-O,O-^Me^PyrCO_2_)­(η^2^-O,N-^Me^PyrCO_2_)] units in **K** ^**Me**^ **Carbamate** at the 80% probability level.

After the discovery of effective capture of CO_2_ through the transformation of **M** ^**R**^ **Pyr** into **M** ^**R**^ **Carbamates**, efforts were further made to regenerate **M** ^**R**^ **Pyr** via incubation of **M** ^**R**^ **Carbamates** under a heat bath and vacuum. As shown in Figure 6a, **K** ^**Me**^ **Carbamate** exhibited an inert nature under vacuum at 125 °C (or 150 °C). Upon elevation of the temperature to 175 °C (or 200 °C), in contrast, the shift of ^13^C MAS NMR signals from (15.1, 106.6, 131.7, 149.1, 152.1) ppm to (14.9, 102.6, 140.1, 148.0) ppm supported the successful regeneration of **K** ^**Me**^ **Pyr** from **K** ^**Me**^ **Carbamate** (Scheme 1d). Moreover, effective regeneration of **K** ^**Me**^ **Pyr** from **K** ^**Me**^ **Carbamate** was further corroborated based on the ^1^H NMR analysis (Figure S4a). Under a similar methodology, incubation of **NaCarbamate** under vacuum at 175 °C (or 200 °C) also induced effective regeneration of **NaPyr** (Figure 6b and S4b). Consequently, CO_2_-induced conversion of **M** ^**R**^ **Pyr** into **M** ^**R**^ **Carbamates** and heat-/vacuum-triggered transformation of **M** ^**R**^ **Carbamates** into **M** ^**R**^ **Pyr** established a TVSA process for capture of CO_2_ (Scheme 1b,d).

**Figure 6:**
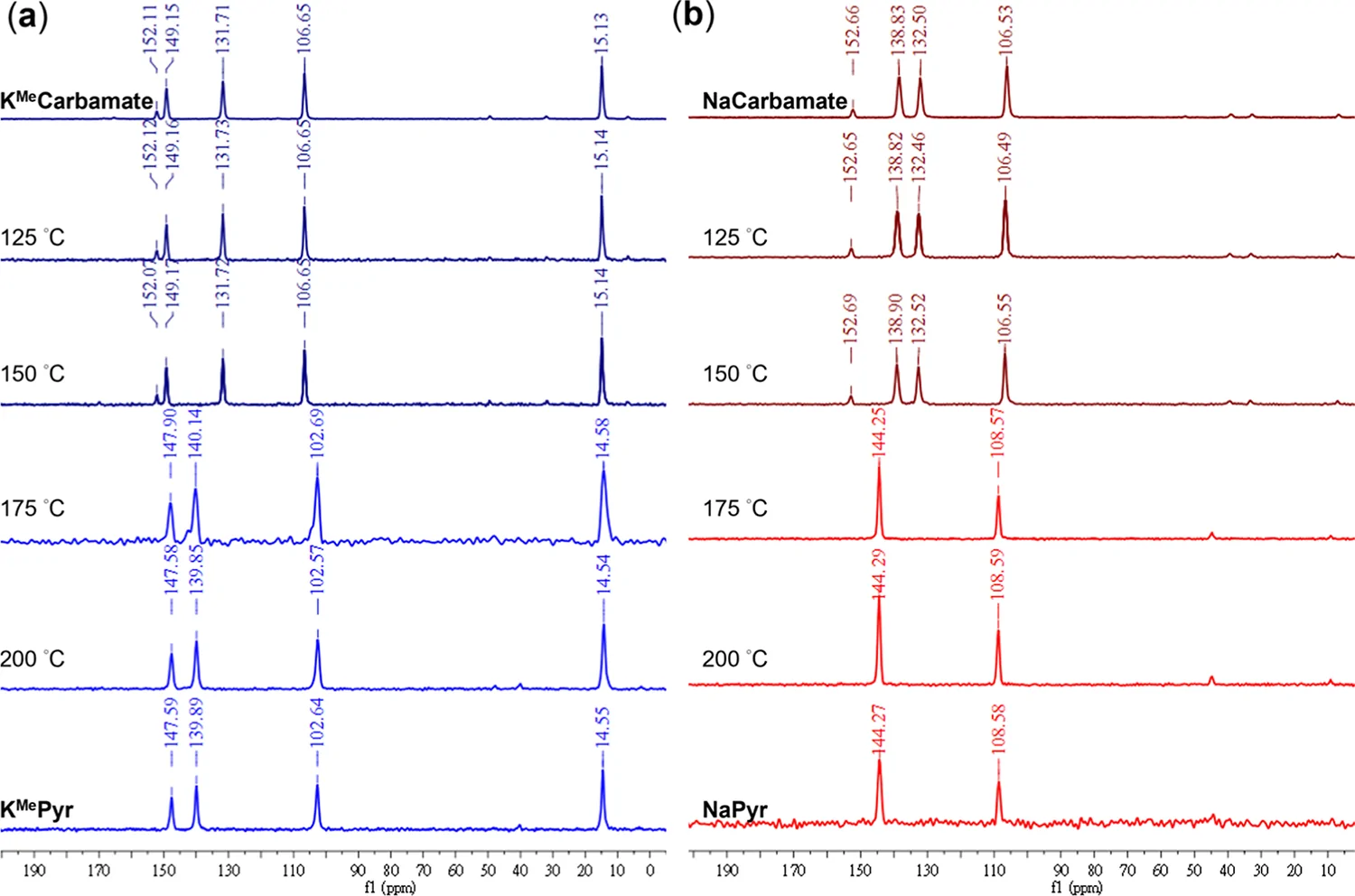
^13^C MAS NMR spectra for (a) **K** ^**Me**^ **Carbamate** and (b) **NaCarbamate** incubated in a heat bath under vacuum at different temperatures for 1 h. ^13^C MAS NMR spectra for as-prepared **K** ^**Me**^ **Pyr**/**NaPyr** and **K** ^**Me**^ **Carbamate**/**NaCarbamate** are depicted in blue/red and navy/maroon. *: spinning side bands.

### Efficacy and Kinetics of Moisture-Gated Capture of CO_2_ Directly from Air Using the **M** ^**R**^ **Pyr** Coordination Polymer

Similar to the assembly of **K** ^**Me**^ **Carbamate** via the reaction of **K** ^**Me**^ **Pyr** and CO_2(g)_, exposure of solid-state **K** ^**Me**^ **Pyr** to ambient air resulted in the formation of an IR absorption peak at 1681 cm^–1^, which indicated the formation of **K** ^**Me**^ **Carbamate** (Scheme 1e and Figure S5). Of interest, retarded transformation of **K** ^**Me**^ **Pyr** into **K** ^**Me**^ **Carbamate** was observed upon treatment with low-humidity air (Scheme S1b and Figure S5). Inspired by the effective capture of CO_2_ from ambient air under static conditions, a DAC system was further assembled to evaluate the CO_2_-capture capacities, kinetics, and mechanisms of **M** ^**R**^ **Pyr** under a continuous flow of ambient, low-humidity, and humidified air streams across varying relative humidity levels at room temperature (Scheme S2).

Breakthrough curves for DAC using the different **M** ^**R**^ **Pyr** are depicted in Figure 7a–c. As opposed to the negligible reactivity of **K** ^**Me**^ **Pyr** toward CO_2_ under a low-humidity air stream (5% RH), the efficient capture of low-concentration CO_2_ by **K** ^**Me**^ **Pyr** from ambient air (20%–40% RH) resulted in an instantaneous reduction of the CO_2_ concentration from 490–560 ppm to 5–10 ppm (Figure 7b). After exposure of **K** ^**Me**^ **Pyr** to a continuous flow of ambient air for 4.5 h, increase of CO_2_ concentration to that same as ambient air indicates the saturation of **K** ^**Me**^ **Pyr** with CO_2_. Through comparison with the concentration of CO_2_ at the inlet, integration of the breakthrough curve reveals a CO_2_-capture capacity of 7.4 ± 0.3 mmol-CO_2_/g-sorbent featured by **K** ^**Me**^ **Pyr** under a continuous flow of ambient air. Similarly, the other metal pyrazolate coordination polymers (**KPyr**/**NaPyr**) also displayed moisture-gated CO_2_ capture profiles under a continuous flow of ambient air (Figures 7a,c). Significantly, the effective DAC capacities of 7.4 ± 0.3, 8.3 ± 0.1, and 9.2 ± 0.3 mmol-CO_2_/g-sorbent exhibited by **K** ^**Me**^ **Pyr**, **KPyr**, and **NaPyr**, respectively, correspond to exceptional site utilization efficiencies of 0.89 ± 0.04, 0.88 ± 0.01, and 0.83 ± 0.03 (Table 1), which closely approach the theoretical limit of one CO_2_ molecule per pyrazole active site. Of importance, the simultaneous achievement of effective DAC capacities (7.4–9.2 mmol-CO_2_/g-sorbent) and substantial site utilization efficiencies (0.83–0.89) by the **M** ^**R**^ **Pyr** platform significantly surpasses the performance metrics typical of conventional DAC sorbents spanning from alkaline metal (hydr)­oxides and hydroxide-based (porous) materials to various guanidine-based platform and (self-)­supported solid amines (DAC capacity generally <4.2 mmol/g; site utilization efficiency ≤ ∼0.80; Table 1). This unique feature underscores the exquisite selectivity of the **M** ^**R**^ **Pyr** platform for CO_2_ over atmospheric moisture, which initiates rather than inhibits CO_2_ capture reactivity. Motivated by the moisture-gated and highly selective capture of low-concentration CO_2_ directly from ambient air using the **M** ^**R**^ **Pyr** platform, the effects of humidity and flow rate of the ambient air stream on CO_2_-capture efficacy and kinetics are further examined below.

**Figure 7:**
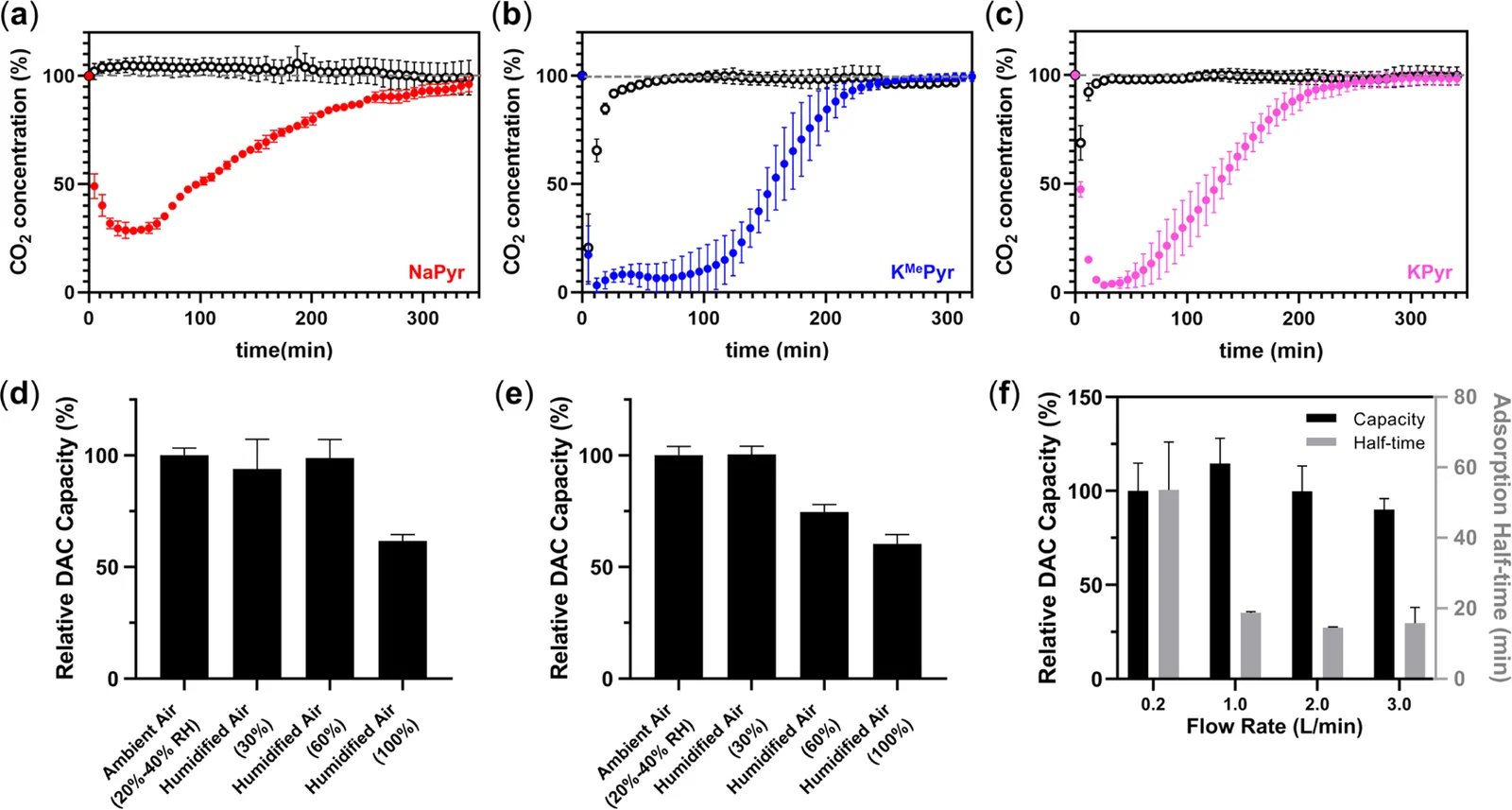
(a–c) Breakthrough curves for DAC of CO_2_ using as-prepared (a) **NaPyr** (solid red dot), (b) **K** ^**Me**^ **Pyr** (solid blue dot), and (c) **KPyr** (solid magenta dot) under a continuous flow of low-humidity air (open black circle) and ambient air (solid circle) at a flow rate of 0.2 L/min. The dashed gray line represents the atmospheric CO_2_ level (490–560 ppm). (d,e) Relative DAC capacities of (d) **NaPyr** and (e) **K** ^**Me**^ **Pyr** under a continuous flow of humidified air at different relative humidity levels (flow rate = 0.2 L/min), which were normalized to the DAC capacities of **NaPyr** and **K** ^**Me**^ **Pyr**, respectively, under a continuous flow of ambient air (20%–40% RH). (f) Effect of inlet air flow rate on the relative DAC capacity and CO_2_ adsorption half-time of **K** ^**Me**^ **Pyr** under a continuous flow of ambient air (20–40% RH). Data show the mean ± standard deviation (SD) from independent experiments.

Given that humidity levels vary considerably under realistic DAC conditions, the CO_2_-capture reactivities of **K** ^**Me**^ **Pyr** and **NaPyr** toward humidified air with varying humidity levels were systematically investigated. As shown in Figure 7d, **NaPyr** sustained a comparable relative DAC capacity across ambient air (20%–40% RH) and humidified air up to 60% RH, with a noticeable decline observed at 100% RH. These profiles demonstrated that the moisture-gated activation of **NaPyr** for DAC remained highly effective across a broad humidity spectrum (20%–60% RH), which underscored the operational versatility and technological relevance of the **NaPyr** platform for potential DAC deployment under diverse environmental conditions. In comparison, **K** ^**Me**^ **Pyr** maintained its capacity only up to 40% RH but experienced progressive capacity loss at 60% RH and 100% RH (Figure 7e). Presumably, this differential sensitivity of **NaPyr** and **K** ^**Me**^ **Pyr** to humidity may mirror their distinct H_2_O adsorption isotherms (Figure S26d,f). Namely, the earlier onset of water uptake exhibited by **K** ^**Me**^ **Pyr** rendered its susceptibility to moisture-induced attenuation of the DAC activity under high-humidity conditions.

To quantitatively evaluate the adsorption kinetics of **M** ^**R**^ **Pyr** under DAC conditions, time-dependent CO_2_ uptake profiles were derived by integrating the breakthrough curves presented in Figures 7a–c and S6. These kinetic profiles were subsequently analyzed using the classical kinetic models, including pseudo-first-order, pseudo-second-order, and Elovich models.^73,74^ As shown in Figure S6,S7, fitting the kinetic profiles to the pseudo-first-order model yielded the best fit among all the tested models (*R* ^2^ = 0.99–0.97). Based on the best fit, the adsorption rate constants (*k*_ad_) of 1.13 × 10^–2^ min^–1^, 1.02 × 10^–2^ min^–1^, and 4.51 × 10^–3^ min^–1^ and adsorption half-time (*t*_1/2_) of 53.6, 68, and 145 min were determined for **K** ^**Me**^ **Pyr**, **KPyr**, and **NaPyr**, respectively. That is, despite the initially nonporous nature of the as-prepared **M** ^**R**^ **Pyr**, these results confirm that the moisture-gated structural rearrangement effectively facilitates rapid gas diffusion and efficient site accessibility.

In the attempt to maximize the kinetic efficiency of the DAC process, the effect of inlet air flow rate on the CO_2_ adsorption half-time and DAC capacity of **K** ^**Me**^ **Pyr** was further evaluated, using a CO_2_:humidity meter (LI-850, LI-COR) for online dual monitoring of H_2_O/CO_2_ concentrations in the outlet of the DAC system. As shown in Figure S6a, the rapid decrease of outlet CO_2_ and H_2_O concentrations to their respective minima within 1 and 5 min, respectively, indicates rapid gas-phase uptake of both H_2_O and CO_2_ by **K** ^**Me**^ **Pyr** under DAC conditions. As the flow rate was increased, the CO_2_ adsorption half-time decreased substantially from 53.6 ± 13.6 min (0.2 L/min) to 18.8 ± 0.3 min (1.0 L/min) and 14.6 ± 0.2 min (2.0 L/min), while comparable DAC capacities were retained across these flow rates (Figure 7f). At the highest flow rate examined (3.0 L/min), the relative DAC capacity declined to ∼90% with a comparable CO_2_ adsorption half-time of 15.8 ± 4.5 min. These results identify 1.0 L/min as the optimal flow rate that enhances adsorption kinetics while minimizing the energy input associated with air delivery and preserving the DAC capacity of **K** ^**Me**^ **Pyr**. While CO_2_ adsorption kinetics is governed by the intrinsic kinetics and thermodynamic capacity of the DAC sorbent, as well as the extrinsic factors such as the inlet flow rate and CO_2_ concentration, this optimized CO_2_ adsorption half-time of **K** ^**Me**^ **Pyr** (18.8 min at 1.0 L/min) lies within the 1–960 min range reported for other DAC sorbents.^75–77^

### Mechanistic Investigation on Moisture-Gated Capture of CO_2_ Directly from Air Using **M** ^**R**^ **Pyr** Coordination Polymers

After effective and moisture-gated capture of CO_2_ from the ambient air using **M** ^**R**^ **Pyr** coordination polymers, the CO_2_-capture adducts isolated from the DAC system were characterized using *ex situ* IR, ^13^C MAS NMR, and PXRD (Figures S8–S10). Of importance, the diagnostic IR ν_CO_ absorption peaks, PXRD patterns, and ^13^C MAS NMR signals support that moisture-gated chemisorption of CO_2_ by **M** ^**R**^ **Pyr** led to the dominant assembly of **M** ^**R**^ **Carbamate,** rather than MHCO_3_ or M_2_CO_3_, under a continuous flow of ambient air (Scheme 1e), consistent with that observed upon the reaction of **M** ^**R**^ **Pyr** with pure CO_2(g)_ (Scheme 1b). As shown in Figure 8a, in particular, the experimental PXRD pattern of **K** ^**Me**^ **Carbamate** derived from DAC using **K** ^**Me**^ **Pyr** closely matches the calculated PXRD pattern generated from the crystal structure of **K** ^**Me**^ **Carbamate**, indicating the minimal presence of residual H_2_O in DAC-derived **K** ^**Me**^ **Carbamate**. On the other hand, the reactivity of **NaPyr** in THF, as well as solid-state ([(Na-18-crown-6-ether)­(Na)]­[Pyr]_2_)_2_, toward low-humidity air, ambient air, and CO_2(g)_, respectively, was further investigated to unravel the mechanism underlying the moisture-gated capture of CO_2_ from ambient air by **M** ^**R**^ **Pyr** (see Supplementary Results and Discussion).

**Figure 8:**
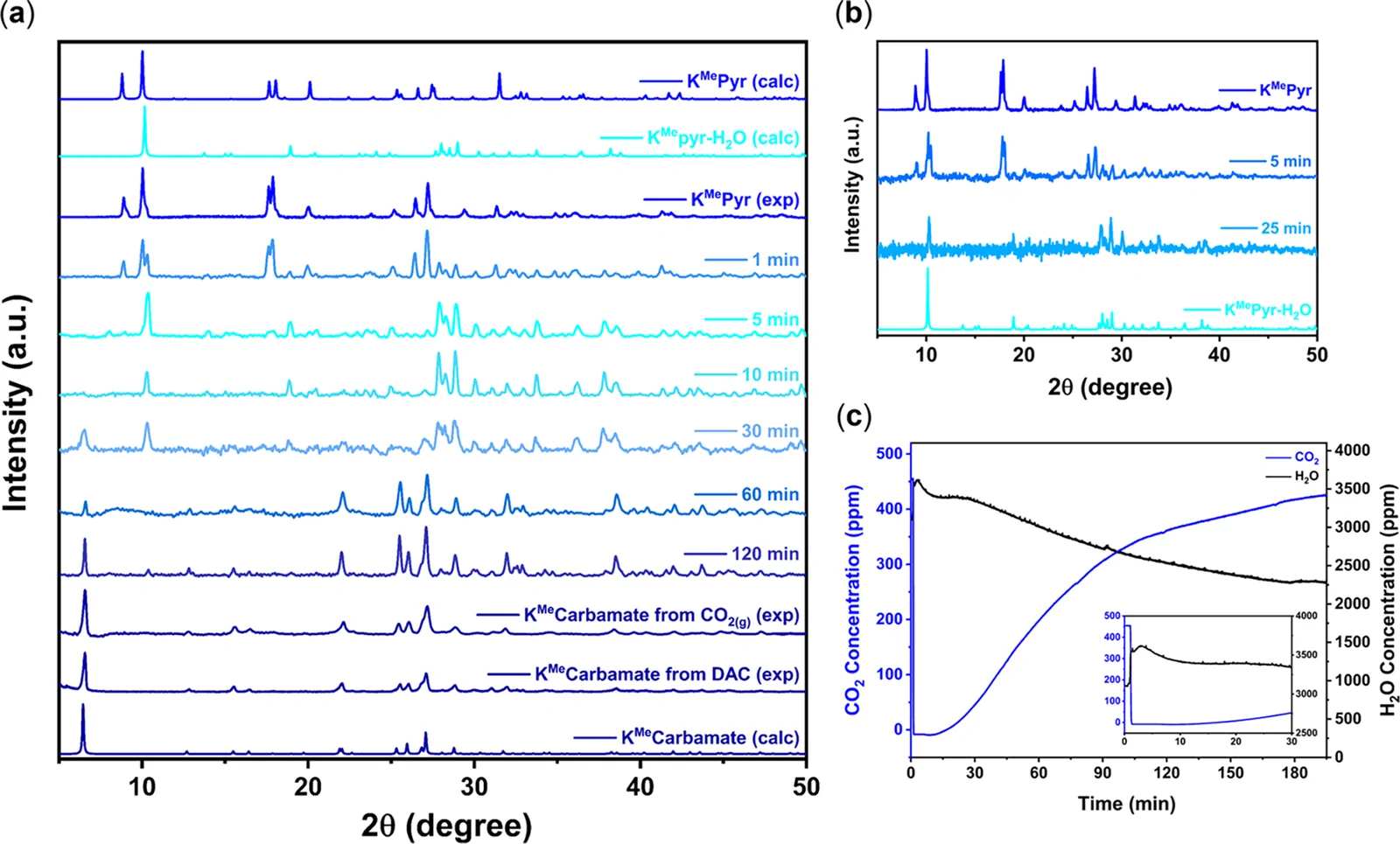
(a) PXRD patterns of as-prepared **K** ^**Me**^ **Pyr** before and after exposure to a continuous flow of ambient air for the indicated time periods. These *ex situ* PXRD patterns are compared with the calculated PXRD patterns of **K** ^**Me**^ **Pyr**, **K** ^**Me**^ **Pyr**·**H**_**2**_ **O**, and **K** ^**Me**^ **Carbamate**, as well as the experimental PXRD patterns of **K** ^**Me**^ **Carbamate** obtained from the reaction of **K** ^**Me**^ **Pyr** with pure CO_2(g)_ and from DAC using **K** ^**Me**^ **Pyr**. (b) PXRD patterns of as-prepared **K** ^**Me**^ **Pyr** before (blue) and after exposure to CO_2_-free air flow for 5 and 25 min. Calculated PXRD spectrum of single-crystal hydrated **K** ^**Me**^ **Pyr**·**H**_**2**_ **O** was depicted in cyan. (c) CO_2_ capture (blue) and H_2_O release (black) profiles for **K** ^**Me**^ **Pyr**·**H**_**2**_ **O** upon exposure to a continuous flow of low-humidity air (flow rate = 0.2 L/min).

Complementary to these reactivity studies, structural analysis of hydrated **K** ^**Me**^ **Pyr** was performed to obtain direct crystallographic insight into the operative mechanism for moisture-gated capture of CO_2_ from ambient air, leading to the formation of **K** ^**Me**^ **Carbamate**. The single-crystal X-ray structure of hydrated **K** ^**Me**^ **Pyr** (**K** ^**Me**^ **Pyr**·**H**_**2**_ **O**) is illustrated in Figure 1c,d and S11, which provides direct insight into the hydration-induced structural rearrangement through comparison with the single-crystal structure of **K** ^**Me**^ **Pyr** (Figure 1a,b). As discussed above, cation–π interactions between each planar [K­(μ-^Me^Pyr)_2_K] unit facilitated the buildup of the double-ladder chain structure of **K** ^**Me**^ **Pyr**. Upon hydration, the geometry of each [K­(μ-^Me^Pyr)_2_K] unit distorted from a planar form in **K** ^**Me**^ **Pyr** into a chair form-like structure in **K** ^**Me**^ **Pyr**·**H**_**2**_ **O**, in order to accommodate one H_2_O molecule between each pair of [K­(μ-^Me^Pyr)_2_K] units. In addition, for each [K­(μ-^Me^Pyr)_2_K] unit in **K** ^**Me**^ **Pyr**, the coordination environment of each K^+^ center was completed through cation–π interactions with two 3-methylpyrazolate ligands in the longitudinal direction (along with the *c*-axis), namely through [K­(η^2^-^Me^Pyr)], [K­(η^5^-^Me^Pyr)], and [K­(η^4^-^Me^Pyr)] cation–π interactions. In **K** ^**Me**^ **Pyr**·**H**_**2**_ **O**, besides one [K­(η^5^-^Me^Pyr)] cation–π interaction, the other cation–π interaction observed in **K** ^**Me**^ **Pyr** was replaced by coordination of two H_2_O toward each K^+^ in the [K­(μ-^Me^Pyr)_2_K] unit (K–OH_2_ bond distances = 2.844 Å and 3.047 Å). Moreover, this coordination of H_2_O toward K^+^ was stabilized with the hydrogen-bonding interaction between H_2_O and proximal 3-methylpyrazolate ligand (N_MePyr_···H_2_O bond distance = 1.913 Å). Accordingly, this hydration process perturbed the parallel/compact stacking of [K­(μ-^Me^Pyr)_2_K] units in the double-ladder chain structure of **K** ^**Me**^ **Pyr** and promoted the rearrangement of [K­(μ-^Me^Pyr)_2_K] units into a zig-zag architecture for the accommodation of H_2_O molecules in **K** ^**Me**^ **Pyr**·**H**_**2**_ **O**. As shown in Figure 1d, each one-dimensional coordination polymer in **K** ^**Me**^ **Pyr**·**H**_**2**_ **O** was surrounded by four identical chains. Unlike the absent interaction between each chain in **K** ^**Me**^ **Pyr** along with the longitudinal direction, a series of K···C_MePyr_ interactions (with K···C_MePyr_ distance = 3.196 Å) in **K** ^**Me**^ **Pyr**·**H**_**2**_ **O** was observed to provide a driving force for stacking of one-dimensional chain into a three-dimensional structure (Figures 1d and S11).

Inspired by the structural analyses on **K** ^**Me**^ **Pyr**, **K** ^**Me**^ **Pyr**·**H**_**2**_ **O**, and **K** ^**Me**^ **Carbamate**, exposure of **K** ^**Me**^ **Pyr** to ambient air (20%–40% RH) for different time periods coupled with *ex situ* PXRD characterizations was further performed. As shown in Figure 8a, exposure of **K** ^**Me**^ **Pyr** to ambient air for 1 min resulted in the rapid emergence of **K** ^**Me**^ **Pyr**·**H**_**2**_ **O** alongside unreacted **K** ^**Me**^ **Pyr**, with complete conversion to **K** ^**Me**^ **Pyr**·**H**_**2**_ **O** observed at 5–10 min. Moreover, extended exposure to ambient air then triggered the replacement of H_2_O with CO_2_, yielding **K** ^**Me**^ **Carbamate**, which first appeared at 30 min and fully replaced **K** ^**Me**^ **Pyr**·**H**_**2**_ **O** by 60–120 min. The resulting PXRD pattern at 120 min was consistent with that of independently synthesized **K** ^**Me**^ **Carbamate** (Figure 8a). These results demonstrate a sequential moisture-gated mechanism “**K** ^**Me**^ **Pyr** → **K** ^**Me**^ **Pyr**·**H**_**2**_ **O** → **K** ^**Me**^ **Carbamate**” for the effective and selective capture of CO_2_ from ambient air.

Building on the sequential mechanism established above, the discrete **K** ^**Me**^ **Pyr** → **K** ^**Me**^ **Pyr**·**H**_**2**_ **O** and **K** ^**Me**^ **Pyr**·**H**_**2**_ **O** → **K** ^**Me**^ **Carbamate** steps were further validated by synthesizing hydrated **K** ^**Me**^ **Pyr**·**H**_**2**_ **O** and examining its subsequent CO_2_-capture reactivity toward low-humidity air (Scheme 1f,g). After the exposure of **K** ^**Me**^ **Pyr** to CO_2_-free air for 25 min, hydrated **K** ^**Me**^ **Pyr** exhibited a PXRD pattern (Figure 8b) that was fully indexed to the (110), (021), (212), (122), (231), (113), and (023) facets based on the simulated patterns of the single-crystal structure of **K** ^**Me**^ **Pyr**·**H**_**2**_ **O**. As shown in Figures 8c and S12, **K** ^**Me**^ **Pyr**·**H**_**2**_ **O** displayed an efficient nature for capture of CO_2_ directly from low-humidity air (Scheme 1g and Table 1; CO_2_-capture capacity = 5.8 ± 0.5 mmol-CO_2_/g-sorbent, adsorption rate constants = 1.77 × 10^–2^ min^–1^, CO_2_ adsorption half-time = 36.3 min), which is in contrast to the inert nature of the as-prepared **K** ^**Me**^ **Pyr**. During this DAC process of **K** ^**Me**^ **Pyr**·**H**_**2**_ **O** against low-humidity air, of importance, concomitant capture of CO_2_ and release of H_2_O supported the competitive binding of CO_2_ toward pyrazolate active site in **K** ^**Me**^ **Pyr**·**H**_**2**_ **O,** overwhelming the captured H_2_O derived from the preceding hydration process (Figure 8c). This displacement of H_2_O in **K** ^**Me**^ **Pyr**·**H**_**2**_ **O** by incoming CO_2_ was also corroborated by the CO_2_/H_2_O breakthrough profiles of as-prepared **K** ^**Me**^ **Pyr** under a continuous flow of ambient air (Figure S6a), in which the outlet H_2_O concentration, after reaching a minimum, progressively increased, whereas the outlet CO_2_ concentration remained low.

Based on the crystallographic insights and reactivity studies discussed above, a stepwise mechanism for moisture-gated, MLC, and highly selective capture of CO_2_ directly from ambient air by **K** ^**Me**^ **Pyr** is elucidated. Regarding the moisture-gated activation process, MLC hydration of **K** ^**Me**^ **Pyr** (mediated by synergistic [K···O_water_] and [N_MePyr_···H_water_] interactions) triggers the intercalation of water molecules into the compact [K­(μ-^Me^Pyr)_2_K] layers assembled via cation–π interactions within the double-ladder chain structure. This hydration process induces a substantial structural rearrangement of **K** ^**Me**^ **Pyr**, expanding the [K­(μ-^Me^Pyr)_2_K] layers into a zig-zag architecture and elongating the interlayer separation from ∼5.7 Å (the K_1_···K_2_ and K_3_···K_3_ distances) in **K** ^**Me**^ **Pyr** to ∼7.7 Å (the K_1_···K_1_ distances) in **K** ^**Me**^ **Pyr**·**H**_**2**_ **O** (Figure 1a,c). Moreover, computational pore analysis reveals the emergence of 1D connectivity/pathway as evidenced by the increase in percolated dimensions from 0 for **K** ^**Me**^ **Pyr** to 1 for **K** ^**Me**^ **Pyr**·**H**_**2**_ **O** (Figure S13). Physically, this moisture-gated expansion relieves the steric shielding of the pyrazolate motifs, thereby permitting exogenous CO_2_ to access these nucleophilic active sites. Chemically, the coordination of water attenuates the Lewis acidity of the K^+^ centers, effectively unleashing the intrinsic nucleophilicity of the anionic pyrazolate ligands to initiate the moisture-gated CO_2_ capture.

While moisture in the air initiates MLC activation of **K** ^**Me**^ **Pyr** and triggers its structural transformation into **K** ^**Me**^ **Pyr**·**H**_**2**_ **O**, comparison of the structures of **K** ^**Me**^ **Pyr**·**H**_**2**_ **O** and **K** ^**Me**^ **Carbamate** provides direct crystallographic insights into thermodynamic preference for the selective binding of CO_2_ over H_2_O. As shown in Figures 5b–d and 1c, the metal carbamate linkage ([N_MePyr_-CO_2_···K]) in **K** ^**Me**^ **Carbamate** is stabilized by both covalent N–C bond within the anionic [^Me^PyrCO_2_]^−^ ligand and Lewis acid–base bonding interaction between this ligand and the K^+^ cation, whereas the [N_MePyr_···H_2_O···K] unit in **K** ^**Me**^ **Pyr**·**H**_**2**_ **O** relies on hydrogen-bonding and weaker Lewis acid–base bonding interactions between the neutral H_2_O molecule and the K^+^ cation. Moreover, in contrast to the intercalation of water molecules into the [K­(μ-^Me^Pyr)_2_K] layers in **K** ^**Me**^ **Pyr**·**H**_**2**_ **O**, the bridging coordination modes of the anionic [^Me^PyrCO_2_]^−^ ligands observed in the [K_5_(μ^5^-^Me^PyrCO_2_)] and [K­(η^1^-O-^Me^PyrCO_2_)_3_(η^2^-O,O-^Me^PyrCO_2_)­(η^2^-O,N-^Me^PyrCO_2_)] units in **K** ^**Me**^ **Carbamate** provide an additional thermodynamic driving force for the effective displacement of bound water by incoming CO_2_. Consequently, these structural comparisons rationalize the stoichiometric transformation of **K** ^**Me**^ **Pyr**·**H**_**2**_ **O** into **K** ^**Me**^ **Carbamate** accompanied by the concomitant release of moisture back into the atmosphere.

In contrast to conventional DAC chemisorbents ranging from alkaline metal (hydr)­oxides and hydroxide-based (porous) materials to various guanidine-based platform and (self-)­supported solid amines (Table 1), this MLC and moisture-gated mechanism of the metal pyrazolate coordination polymer establishes a distinct paradigm in the design of DAC sorbents for highly selective capture of CO_2_ over H_2_O directly from ambient air stream. Distinct from neutral amines typically constrained by a 2:1 stoichiometry for the capture of CO_2_ (2 R_2_NH + CO_2_ → [R_2_NH_2_]­[R_2_NCO_2_]), this anionic pyrazolate system facilitates a highly atom-economical 1:1 capture pathway ([Metal]­[^R^Pyr] + CO_2_ → [Metal]­[^R^Carbamate]). Furthermore, competitive inhibition by atmospheric moisture in conventional DAC chemisorbents typically precludes the realization of theoretical site utilization (e.g., 2:1 for amines and 1:1 for metal (hydr)­oxides). In the metal pyrazolate coordination polymer, conversely, atmospheric moisture serves as a transient allosteric switch to unlock the atom-economical 1:1 stoichiometry for CO_2_ capture without compromising selectivity.

### Regeneration Thermodynamics and Kinetics of DAC-Derived **M** ^**R**^ **Carbamate**

After saturation of **NaPyr**/**K** ^**Me**^ **Pyr** with CO_2_ from ambient air stream using the DAC system, incubation of the obtained **NaCarbamate**/**K** ^**Me**^ **Carbamate** adducts under vacuum at 200 °C triggered the effective regeneration of **NaPyr**/**K** ^**Me**^ **Pyr** (Scheme 1h, Figures 9a,b and S14). Notably, the requisite regeneration temperature of 200 °C for the **M** ^**R**^ **Pyr** coordination polymer lies between those of conventional alkaline earth metal (hydr)­oxides (typically ≥700 °C) and hydroxide-based (porous) materials/guanidine-based platform/solid amines (typically 60 °C–150 °C) compiled in Table 1.

**Figure 9:**
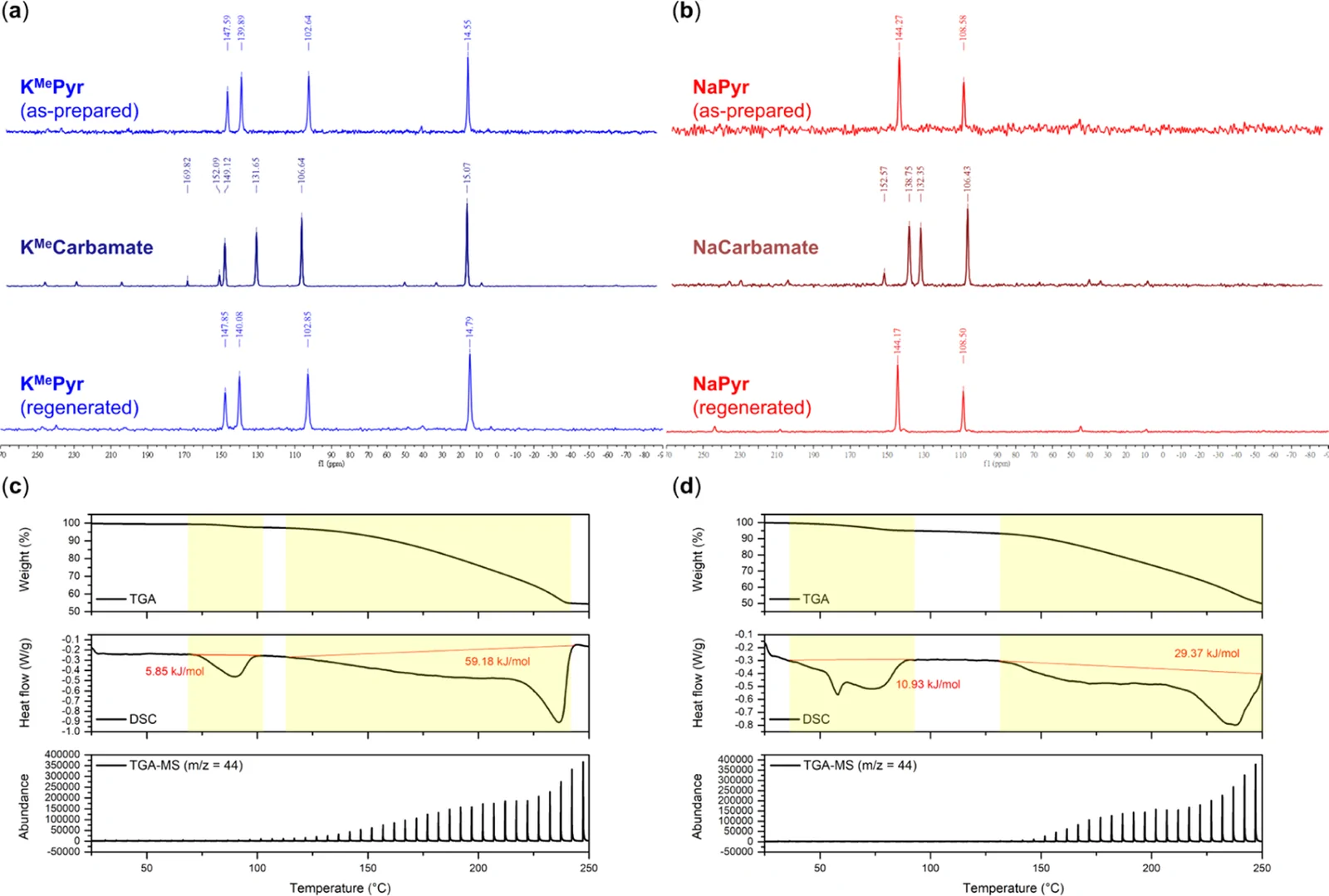
^13^C MAS NMR spectra for (a) as-prepared **K** ^**Me**^ **Pyr** (top), **K** ^**Me**^ **Carbamate** derived from DAC using **K** ^**Me**^ **Pyr** (middle), regenerated **K** ^**Me**^ **Pyr** obtained from incubation of **K** ^**Me**^ **Carbamate** under vacuum at 200 °C for 1 h (bottom) and (b) as-prepared **NaPyr** (top), **NaCarbamate** derived from DAC using **NaPyr** (middle), regenerated **NaPyr** obtained from incubation of **NaCarbamate** under vacuum at 200 °C for1 h (bottom). *: spinning side bands. (c,d) TGA (top), DSC (middle), and TGA-MS (for CO_2_ at *m*/*z* = 44, bottom) analyses of (c) **K** ^**Me**^ **Carbamate** and (d) **NaCarbamate** derived from DAC using **K** ^**Me**^ **Pyr** and **NaPyr**, respectively.

Beyond the regeneration temperature, the regeneration energy represents another critical metric for evaluating the technological viability and energetic efficiency of DAC sorbents.^22,71^ Consequently, the thermodynamic and kinetic profiles for the heat-induced desorption of CO_2_ from **M** ^**R**^ **Carbamate** were systematically investigated using synchronized TGA–DSC coupled with MS analysis. As shown in Figure 9c, the TGA curve of **K** ^**Me**^ **Carbamate** exhibited a primary weight loss step of 42.6% between 114 °C and 243 °C, which was confirmed as CO_2_ desorption by the synchronized MS signals. Based on the DSC analysis, the enthalpies required for the desorption of CO_2_ from **K** ^**Me**^ **Carbamate** (Δ*H*_des_) and the overall regeneration of **K** ^**Me**^ **Pyr** (Δ*H*_reg_) were quantified to be 59.2 kJ/mol-sorbent and 65.0 kJ/mol-sorbent, respectively. Under a similar experimental methodology, **KCarbamate**/**NaCarbamate** displayed Δ*H*_des_ of 63.2/29.4 kJ/mol-sorbent and Δ*H*_reg_ of 65.0/40.3 kJ/mol-sorbent (Figures S15 and 9d). When normalized to the measured CO_2_/active site molar ratio for each metal pyrazolate coordination polymer, these Δ*H*_reg_ values corresponded to overall regeneration energies of 73.0, 73.9, and 48.6 kJ/mol-CO_2_ for **K** ^**Me**^ **Carbamate**, **KCarbamate**, and **NaCarbamate**, respectively (Table 1). Through a systematic benchmarking comparison with various state-of-the-art DAC sorbents compiled in Table 1, the **M** ^**R**^ **Pyr** coordination polymers feature regeneration energies of 49–74 kJ/mol-CO_2_, which are lower than those of 80–286 kJ/mol-CO_2_ displayed by other solid-state DAC sorbents (Table 1) as well as monoethanolamine and amine-based solutions.^22,30,36,43,47,70,71,78^ This energetic efficiency, presumably, is enabled by the solid-state nature of the **M** ^**R**^ **Pyr** coordination polymers, the thermodynamic reversibility of CO_2_ adsorption and desorption, and the exceptional selectivity for the capture of CO_2_ over H_2_O under DAC. Despite the low regeneration energy of **M** ^**R**^ **Pyr**, its regeneration temperature of 200 °C remains higher than desirable for large-scale operation, which motivates continued efforts toward reduction of regeneration temperature through modifications of the countercation (e.g., alkyl ammonium cations) or pyrazolate substituents. On the other hand, to evaluate the kinetics for the desorption of CO_2_ from **M** ^**R**^ **Carbamate**, isothermal TGA–MS measurement was conducted at 200 °C, and the resulting time-dependent CO_2_ release profile of **K** ^**Me**^ **Carbamate** was fitted to zero-order, pseudo-first-order, and pseudo-second-order kinetic models (Figure S16). According to the best fit obtained from the zero-order model, **K** ^**Me**^ **Carbamate** featured a desorption half-time (*t*_1/2_) of 7.8 min.

### Recyclability of **M** ^**R**^ **Pyr** under Temperature–Vacuum Swing Adsorption

Building on the successful regeneration of **NaPyr**/**K** ^**Me**^ **Pyr** from **NaCarbamate**/**K** ^**Me**^ **Carbamate**, a viable TVSA process was implemented to evaluate the recyclability of **NaPyr**/**K** ^**Me**^ **Pyr** via alternative cycles of (a) saturation of as-prepared/regenerated **M** ^**R**^ **Pyr** with CO_2_ from ambient air stream at room temperature and (b) incubation of obtained **M** ^**R**^ **Carbamates** under vacuum at 200 °C for 1 h. As shown in Figure 10, the DAC capacity of **M** ^**R**^ **Pyr** during each cycle was normalized to that of the as-prepared form of corresponding sorbents. Accordingly, the relative DAC capacity of **NaPyr** (or **K** ^**Me**^ **Pyr**) was gradually decreased from 100.0 ± 8.0% (or 100.0 ± 5.7%) in the first cycle to 96.5 ± 9.6% (or 97.2 ± 6.5%) in the third cycle and reached 94.4 ± 9.7% (or 95.0 ± 12.2%) by the fifth cycle. Although the DAC capacity of both **K** ^**Me**^ **Pyr** and **NaPyr** remained above 90% through the five-cycle recyclability test, efforts were further dedicated to unraveling the degradation mechanism that competes with the dominant regeneration of **K** ^**Me**^ **Pyr**/**NaPyr** during the heat–vacuum treatment of **K** ^**Me**^ **Carbamate**/**NaCarbamate** derived from DAC.

**Figure 10:**
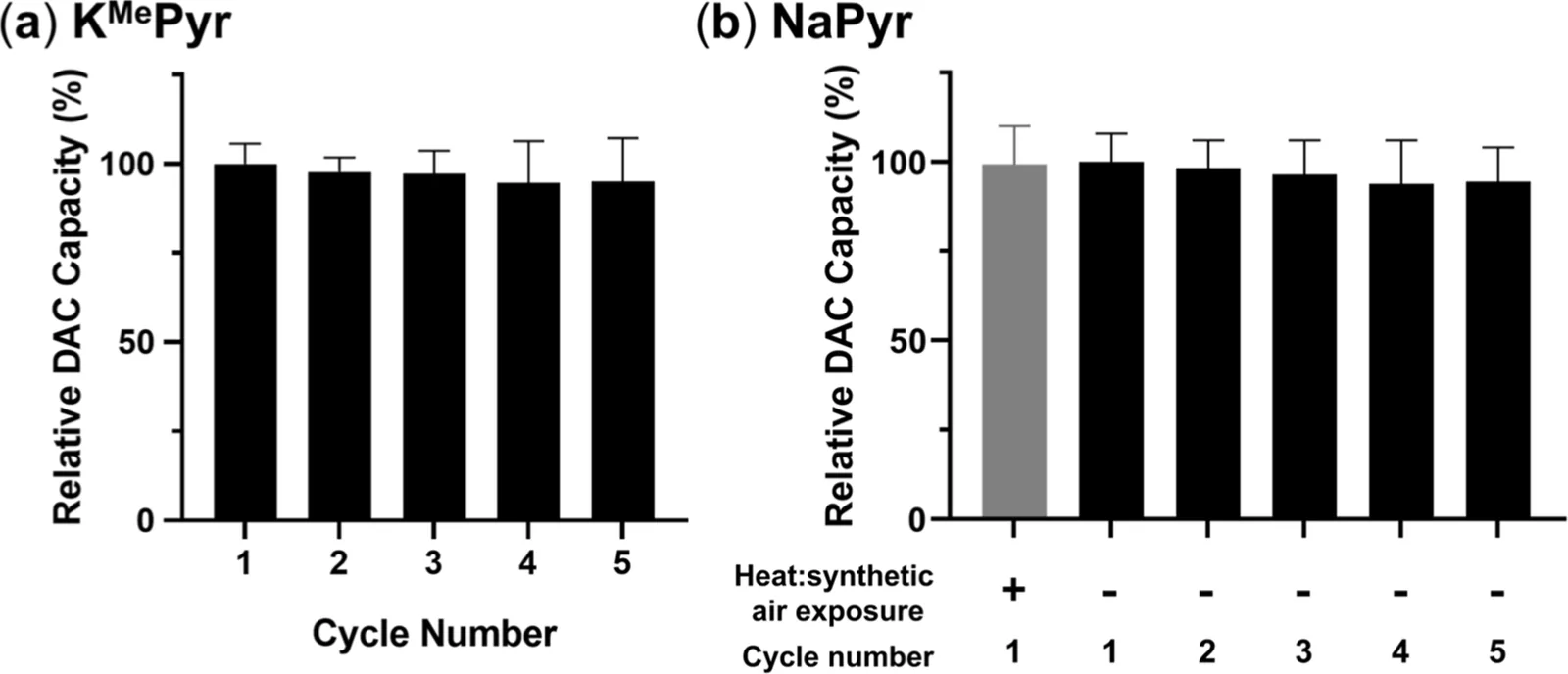
Relative DAC capacity of (a) **K** ^**Me**^ **Pyr** and (b) **NaPyr** during the five-cycle reusability test. Relative DAC capacity of **NaPyr** after exposure to the synthetic air (a mixture gas of 21% O_2(g)_ and 79% N_2(g)_) at 175 ^°^C for 24 h is depicted in gray. Data show the mean ± SD from at least three independent experiments.

During the heat–vacuum treatment of **K** ^**Me**^ **Carbamate**/**NaCarbamate**, in addition to the dominant regeneration of **K** ^**Me**^ **Pyr**/**NaPyr**, minor formation of K_2_CO_3_/Na_2_CO_3_ was observed based on the solid-state IR spectra of obtained products (Figure S17a,b). Moreover, the concomitant formation of 3-methylpyrazole/pyrazole and release of H_2_O were captured and characterized by ^1^H NMR spectroscopy (Figure S17c,d). That is, heat-/vacuum-induced release of captured CO_2_ and trace amounts of H_2_O from **K** ^**Me**^ **Carbamate**/**NaCarbamate** governs the dominant regeneration of **K** ^**Me**^ **Pyr**/**NaPyr** (Scheme S3). However, the elevated temperature under heating, meanwhile, may promote the minor protonation of **K** ^**Me**^ **Carbamate**/**NaCarbamate** by H_2_O and lead to the formation of 3-methylpyrazole/pyrazole and KHCO_3_/NaHCO_3_, the latter of which subsequently converts into K_2_CO_3_/Na_2_CO_3_. Of importance, this degradation mechanism, competing with the regeneration of **K** ^**Me**^ **Pyr**/**NaPyr** upon the heat–vacuum treatment of **K** ^**Me**^ **carbamate**/**NaCarbamate**, provides a mechanistic explanation for the gradual decrease in relative DAC capacity observed during the recyclability test of **K** ^**Me**^ **Pyr**/**NaPyr** under TVSA.

### Stability of **M** ^**R**^ **Pyr** and **M** ^**R**^ **Carbamate** under Extreme Operational Conditions: Oxidative Exposure at Elevated Temperature and Bulk Liquid Water

Beyond the minor protonation-mediated generation of K_2_CO_3_ and 3-methylpyrazole discussed above, the potential for oxidative degradation of (regenerated) **M** ^**R**^ **Pyr** at elevated temperatures, arising from O_2_ exposure during the operational switch between ambient air capture at room temperature and heat–vacuum regeneration, represents an additional concern in the TVSA process. To address this, the oxidative stability of **M** ^**R**^ **Pyr** was evaluated via exposure of **NaPyr** to a synthetic air stream (21% O_2(g)_ and 79% N_2(g)_) at 175 ^°^C for 24 h. As shown in Figures 10d and S18, the heat-/synthetic air-treated **NaPyr** exhibited DAC capacity and mechanism comparable to those of the as-prepared **NaPyr**. These results supported the oxidative stability of **NaPyr** and excluded its oxidative degradation during the TVSA process.

While oxidative degradation represents one extreme operational scenario, exposure to bulk liquid water constitutes another, analogous to precipitation events encountered under real-world DAC deployments. Accordingly, the stability of **K** ^**Me**^ **Pyr** (or **NaPyr**) in liquid water, representing the extreme limit of the humidity spectrum examined above (5–100% RH), was further evaluated. In DMSO-*d*_6_, both **K** ^**Me**^ **Pyr** and **NaPyr** exhibited characteristic ^1^H NMR signals that are distinctive from those of (3-methyl)­pyrazole (Figure S19a,b). Following overnight incubation in D_2_O, the ^1^H NMR signals for **K** ^**Me**^ **Pyr** shifted to (2.24, 6.13, 7.53) ppm, comparable to those at (2.20, 6.08, 7.48) ppm exhibited by 3-methylpyrazole (Figure S19c). As shown in Figure S19d, both **NaPyr** and pyrazole displayed similar ^1^H NMR signals in D_2_O. These comparable ^1^H NMR signals of **M** ^**R**^ **Pyr** and their corresponding free pyrazoles in D_2_O may be ascribed to either extensive hydrogen-bonding interactions or solvent-mediated protonation of **M** ^**R**^ **Pyr** in liquid water. Of importance, upon the removal of D_2_O under vacuum, the recovered solid products displayed ^1^H NMR spectra in DMSO-*d*_6_ identical to those of the original **M** ^**R**^ **Pyr** (Figure S19e,f). These results demonstrate that the incubation of **K** ^**Me**^ **Pyr**/**NaPyr** in liquid water is characterized by reversible hydration without irreversible protonation to (3-methyl)­pyrazole, thereby, underscoring the stability of **M** ^**R**^ **Pyr** in liquid water.

In light of the CO_2_-capture performance of **K** ^**Me**^ **Pyr**·**H**_**2**_ **O** toward low-humidity air and the stability of **K** ^**Me**^ **Pyr** in liquid water, the reactivity of an aqueous solution of **K** ^**Me**^ **Pyr** (or **NaPyr**) toward CO_2_ was investigated. Treatment of aqueous **K** ^**Me**^ **Pyr** (or **NaPyr**) with a stoichiometric amount of CO_2(g)_ resulted in the formation of 3-methylpyrazole and KHCO_3_ (or pyrazole and NaHCO_3_, Figure S20). That is, although the capture of CO_2_ by hydrated **M** ^**R**^ **Pyr** yielded **M** ^**R**^ **Carbamate** following the mechanism described above, the presence of bulk liquid water promotes the protonation of the pyrazolate moiety within **M** ^**R**^ **Carbamate,** leading to the formation of (3-methyl)­pyrazole accompanied by MHCO_3_. This unstable nature of **K** ^**Me**^ **Carbamate** against bulk liquid water was also corroborated by the similar generation of 3-methylpyrazole and KHCO_3_ observed upon the incubation of **K** ^**Me**^ **Carbamate** in liquid water (Figure S21). Furthermore, the long-term stability of **K** ^**Me**^ **Pyr** and its CO_2_-saturated state, **K** ^**Me**^ **Carbamate**, toward gaseous H_2_O from ambient air was evaluated through extended exposure to a continuous flow of ambient air (20%–40% RH). As shown in Figure S9a, after the saturation of **K** ^**Me**^ **Pyr** with CO_2_ from air, extended exposure of the generated **K** ^**Me**^ **Carbamate** to the ambient air stream for 24 h resulted in absent conversion into KHCO_3_ and 3-methylpyrazole. Despite the unstable nature of **K** ^**Me**^ **Carbamate** in bulk liquid water (representing an extreme liquid-phase condition), its stability against low-concentration gaseous moisture ensures the technological relevance and durability of the **M** ^**R**^ **Pyr** platform under realistic DAC conditions. Nevertheless, during practical DAC operations, regeneration of **M** ^**R**^ **Pyr** immediately following its saturation with CO_2_ from ambient air is designed to maximize the operational efficiency and to preserve long-term sorbent durability.

## Conclusion

In this study, the engineering of metal pyrazolate coordination polymers (**M** ^**R**^ **Pyr**) establishes a novel paradigm for the MLC, moisture-gated, and highly selective capture of CO_2_ directly from ambient air stream via the formation of metal carbamate adducts (**M** ^**R**^ **Carbamate**). Exploiting the synergy between nucleophilic anionic pyrazolate ligands and Lewis acidic alkali metal centers, the **M** ^**R**^ **Pyr** platform simultaneously achieves (a) exceptional DAC capacities (7.4–9.2 mmol-CO_2_/g-sorbent), (b) substantial site utilization efficiencies (0.83–0.89), (c) low regeneration energies (49–74 kJ/mol-CO_2_), and (d) reliable multiple-cycle recyclability during the TVSA process. These DAC parameters surpass the persistent trade-offs between intrinsic CO_2_-capture capacity and regeneration energetics alongside the extrinsic challenge of atmospheric moisture inhibition typical of conventional DAC sorbents. Moreover, through crystallographic and spectroscopic investigations, synergistic hydration perturbs the sterically hindered compact packing of one-dimensional double-ladder-like chains in **K** ^**Me**^ **Pyr** and triggers a structural rearrangement into an expanded zig-zag architecture of **K** ^**Me**^ **Pyr**·**H**_**2**_ **O**, which physically relieves steric shielding and chemically unleashes the intrinsic nucleophilicity of the pyrazolate ligands. Subsequently, an exquisite thermodynamic preference for metal carbamate formation drives the effective displacement of bound water by incoming CO_2_, thereby rationalizing the moisture-gated and highly selective capture of CO_2_ by the **M** ^**R**^ **Pyr** platform directly from ambient air streams. Finally, among the investigated **M** ^**R**^ **Pyr** coordination polymers, **NaPyr** sustains effective DAC capacities across a broad humidity spectrum (20%–60% RH) and features remarkable oxidative stability at elevated temperatures. Taken together, these comprehensive attributes underscore the operational versatility and technological relevance of the **M** ^**R**^ **Pyr** platform, particularly **NaPyr** and its (formulated) derivatives, for scalable DAC deployment under diverse and real-world environmental conditions.

## Supplementary Material



## Notes

Additional experimental details, materials, methods, and supplementary results and discussion, including reaction schemes for reactivities of **M** ^**R**^ **Pyr** with CO_2_, regeneration of **M** ^**R**^ **Pyr** from **M** ^**R**^ **Carbamate**, and degradation of **M** ^**R**^ **Carbamate**; schematic illustrations of experimental setups; (solid-state) IR, ^1^H NMR, ^13^C MAS NMR, and experimental/simulated PXRD spectra for all compounds; CO_2_/H_2_O capture breakthrough curves and CO_2_/H_2_O uptake/release profiles for all compounds; single-crystal X-ray structures of **NaCrownPyr** and **K** ^**Me**^ **Pyr**·**H**_**2**_ **O**; space-filling models of **K** ^**Me**^ **Pyr** and **K** ^**Me**^ **Pyr**·**H**_**2**_ **O**; TGA/DSC/TGA–MS analysis of **K** ^**Me**^ **Carbamate** and **KCarbamate**; N_2_/CO_2_ adsorption and H_2_O adsorption–desorption isotherms of **NaPyr**, **KPyr**, and **K** ^**Me**^ **Pyr**; initial model, anisotropic microstrain, and crystallite size of **K** ^**Me**^ **Carbamate**; and crystallographic data, atomic coordinates, displacement parameters, and bond distances and bond angle list of **K** ^**Me**^ **Carbamate** (PDF)
