# Supplementary material for: Engineering Metal Pyrazolate Coordination Polymers for Highly Selective Direct Air Capture via Metal–Ligand Cooperativity and Moisture-Gated Mechanism
Source: J Am Chem Soc. 2026 Oct 2;148(40):43782–98. doi: 10.1021/jacs.6c15929 (PMC13674280; doi:10.1021/jacs.6c15929)
Supplement: Supplementary file 1 [file ja6c15929_si_001.pdf]

Supporting Information

Engineering Metal Pyrazolate Coordination Polymers for Highly Selective Direct Air Capture via Metal-Ligand Cooperativity and Moisture-Gated Mechanism

Tsu-Chieh Yu,[1,$] Chun-Kai Huang,[2,$] Ru-Yun Yang,[1,3,$] Yi-Tsung Lai,[2] Qing-Hui Lin,[1] Ming-Jun Han,[2] Swatilekha Pratihar,[1] Sue-min Chang,[4] Yu-Chen Chung,[2] Bo-Hao Chen,[5*] Yu-Chun Chuang,[5*] Chia-Her Lin,[2*] Ho-Hsiu Chou,[3*] Tsai-Te Lu[1,2,6*]

Affiliations:

[1]Institute of Biomedical Engineering, [2]Department of Chemistry, [3]Department of Chemical Engineering, National Tsing Hua University, Hsinchu, 300044, Taiwan

[4]Institute of Environmental Engineering, National Yang Ming Chiao Tung University, Hsinchu 300093, Taiwan

[5]National Synchrotron Radiation Research Center, Hsinchu 30076, Taiwan.

[6]Department of Chemistry, Chung Yuan Christian University, Taoyuan 320314, Taiwan.

[$]T.-C.Y., C.-K.H., and R.-Y.Y. contributed equally to this paper.

[*]Corresponding author:

Bo-Hao Chen, National Synchrotron Radiation Research Center, Hsinchu 30076, Taiwan. E-mail: chen.bh@nsrrc.org.tw.

Yu-Chun Chuang, Ph.D., National Synchrotron Radiation Research Center, Hsinchu 30076, Taiwan. E-mail: chuang.yc@nsrrc.org.tw.

Chia-Her Lin, Ph.D., Department of Chemistry, National Tsing Hua University, Hsinchu, Taiwan. E-mail: chiaher@mx.nthu.edu.tw.

Ho-Hsiu Chou, Ph.D., Department of Chemical Engineering, National Tsing Hua University, Hsinchu, Taiwan. E-mail: hhchou@mx.nthu.edu.tw.

Tsai-Te Lu, Ph.D., Institute of Biomedical Engineering, Department of Chemistry, National Tsing Hua University, Hsinchu, Taiwan. E-mail: ttlu@mx.nthu.edu.tw.

1. Experimental Section.

Manipulations, reactions, and transfers were conducted under $N_{2(g)}$ according to Schlenk technique or in a glovebox. Organic solvents were distilled under $N_{2(g)}$ from appropriate drying agents (*n*-hexane, diethyl ether, and tetrahydrofuran (THF) from sodium benzophenone) and stored in dried, $N_{2(g)}$-filled flasks over 4 Å molecular sieves. $N_{2(g)}$ was purged through these solvents before use. Solvent was transferred to the reaction vessel and tubes via stainless cannula under positive pressure of $N_{2(g)}$. The reagents pyrazole (Matrix scientific), 3-methylpyrazole (Thermo Scientific), sodium (Thermo Scientific), potassium hydride (Sigma Aldrich), 18-crown-6-ether (TCI), and $^{13}CO_{2(g)}$ (99 atom % ^{13}C, <3 atom % ^{18}O, Sigma Aldrich) were used as received. Complex $(NO)_2Fe(TMEDA)$ (TMEDA = tetramethylethylenediamine) was prepared according to the reported procedures.[1, 2] Powder X-ray diffraction (PXRD) patterns were recorded with an X-ray diffractometer (Bruker D2 Phaser, Bruker D8 ADVANCE Eco, SmartLab SE diffractometer (Rigaku Corporation)) using Cu K_α radiation (λ = 1.5418 Å or 1.5406 Å). Magic angle spinning solid-state ^{13}C NMR (^{13}C MAS NMR) spectra were obtained on a Bruker Avance III 400 NMR Spectrometer. Thermogravimetric analysis (TGA) and differential scanning calorimeter (DSC) study were performed using a Mettler TGA/DSC 2, which is coupled with a GC-MS (Agilent 7890B and Agilent 5977A). For the air-sensitive samples (ex: **K^{Me}Pyr** and **NaPyr**), the samples were loaded into an aluminum crucible standard and sealed with an aluminum crucible lid inside an $N_{2(g)}$-filled glovebox, and the lid was punctured immediately before the crucible was secured under $N_{2(g)}$ flow for TGA measurement. For all other TGA/DSC/TGA-MS measurements, an open-top alumina crucible was used. For variable-temperature TGA/DSC/TGA-MS experiments, the samples were heated from 25 ^{o}C to 250 ^{o}C at a temperature ramp of 5 ^{o}C/min under $N_{2(g)}$ at a flow rate of 35

mL/min; for isothermal TGA/DSC/TGA-MS measurements, the samples were rapidly heated to 200 °C and held at 200 °C under $N_{2(g)}$ at a flow rate of 35 mL/min. Fourier-transform infrared (FT-IR) spectra were recorded on a Perkin-Elmer spectrum two spectrophotometer with pressed KBr pellets or sealed solution cells (0.1 mm, CaF_2 windows). ^1H NMR spectra were obtained on Varian VNMRS-500 MHz spectrometers.

1.1 Preparation of Metal-Pyrazolate Polymers [Metal][^{R}Pyr] (M^{R}Pyr, Metal = Na or K; ^{R}Pyr = pyrazolate for R = H or 3-methylpyrazolate for R = Me). Potassium hydride (4.0 g, 0.1 mol) was loaded into a 300-mL Schlenk flask before 50 mL of THF was added under $N_{2(g)}$ at ambient temperature. After 3-methylpyrazole (8.8 mL, 8.2 g, 0.1 mol) was injected into this 300-mL Schlenk flask using a gas-tight syringe, the reaction solution was stirred at ambient temperature for 12 h. Then, this reaction solution was concentrated before the addition of *n*-hexane (30 mL), which led to the precipitation of white potassium 3-methyl-pyrazolate (**K^{Me}Pyr**) as insoluble powder. The supernatant solution was removed via cannula under positive $N_{2(g)}$ before the white powder of **K^{Me}Pyr** was dried under vacuum (yield 10.06±0.43 g, 83.8±3.6%). ^{13}C MAS NMR (400 MHz) δ = 14.6 (CH_3), 102.6 (N=C-(C)), 139.9 (N-C), 147.6 (N=C) ppm. Crystals of **K^{Me}Pyr** suitable for single-crystal X-ray diffraction analysis were obtained by layering the THF solution of **K^{Me}Pyr** with *n*-hexane at -20 °C for one week.

Under a similar methodology, potassium pyrazolate (**KPyr**) and sodium pyrazolate (**NaPyr**) were prepared through reactions of pyrazole (7.017 g, 0.1 mol) with potassium hydride (4.0 g, 0.1 mol) and sodium (2.3 g, 0.1 mol), respectively. **KPyr**: yield 9.93 g, 73.0%; ^{13}C MAS NMR (400 MHz) δ = 111.19 (N=C-(C)), 139.35 (N-C, 2C) ppm. **NaPyr**: yield 10.68±0.52 g, 89.0±4.3%; ^{13}C MAS NMR (400 MHz) δ = 108.583 (N=C-(C)), 144.275 (N-C, 2C) ppm. Crystals of **NaPyr** suitable for single-crystal X-ray diffraction analysis were obtained by layering the THF solution of **NaPyr**

with *n*-hexane at -20 °C for one week.

1.2 Reaction of M^RPyr with $CO_{2(g)}$ (or Ambient/Low-humidity Air) Yielding Metal Carbamates (M^RCarbamates). Titration of **K^{Me}Pyr** with $CO_{2(g)}$ was executed following the procedure described below. To a 25-mL Schlenk tube containing 10-mL THF solution of **K^{Me}Pyr** (0.120 g, 1.0 mmol), 6 mL of $CO_{2(g)}$ was added via a gas-tight syringe at an ambient temperature. After this reaction solution was stirred for 1 h, this reaction solution was dried under vacuum before the solid-state IR spectrum for the obtained crude product was measured. Solid-state IR spectra for **K^{Me}Pyr** treated with 12 mL, 18 mL, 24 mL, and 30 mL of $CO_{2(g)}$, respectively, were measured under a similar procedure, which revealed a gradual increase of an IR absorption peak at 1681 cm^{-1}. Titration of **KPyr** (or **NaPyr**, 1.0 mmol) with $CO_{2(g)}$ (6 mL, 12 mL, 18 mL, 24 mL, and 30 mL) was performed under a similar methodology.

Preparation of potassium 3-methyl carbamate (**K^{Me}Carbamate**) through reaction of **K^{Me}Pyr** and $CO_{2(g)}$ was executed following the procedure described below. $CO_{2(g)}$ (1 atm, flow rate: 50 mL/min) was purged through a 20-mL THF solution of **K^{Me}Pyr** (0.96 g, 8 mmol) in a 250-mL Schlenk flask for 4.8 min. After this reaction solution was stirred for 30 min, formation of white potassium 3-methyl carbamate (**K^{Me}Carbamate**) as insoluble powder was observed. Then, **K^{Me}Carbamate** was collected after this reaction solution was dried under vacuum. Yield: 1.164±0.089 g, 88.7±6.8%. IR ν_{CO}: 1681 cm^{-1} (KBr). ^{13}C MAS NMR (400 MHz) δ = 152.1 (N-CO_2), 15.1 (CH_3), 106.6 (N=C-(C)), 131.7 (N-C), 149.2 (N=C) ppm. Under a similar methodology, $^{13}CO_2$-labeled **K^{Me}Carbamate** was synthesized via reaction of **K^{Me}Pyr** with $^{13}CO_{2(g)}$. IR ν_{CO}: 1633 cm^{-1} (KBr). Syntheses of potassium carbamate (**KCarbamate**) and sodium carbamate (**NaCarbamate**) were performed under a similar methodology. **KCarbamate**: yield 1.103±0.052 g, 92.0±4.3%. IR ν_{CO}: 1690

cm^{-1} (KBr). ^{13}C MAS NMR (400 MHz) δ = 151.9 (N-CO_2), 106.0 (N=C-(C)), 130.4 (N-C), 140.0 (N=C) ppm. **NaCarbamate**: yield 0.962±0.045 g, 89.7±4.2%. IR ν_{CO}: 1716 cm^{-1} (KBr). ^{13}C MAS NMR (400 MHz) δ = 152.6 (N-CO_2), 106.5 (N=C-(C)), 132.4 (N-C), 138.8 (N=C) ppm. $^{13}CO_2$-labeled **NaCarbamate**: IR ν_{CO}: 1668 cm^{-1} (KBr).

Following the procedures described above, (a) reaction of solid-state **K^{Me}Pyr** (0.5 mmol) with ambient air, (b) reaction of solid-state **K^{Me}Pyr** (0.5 mmol) with low-humidity air, (c) reactions of dry $CO_{2(g)}$/humid $CO_{2(g)}$/ambient air/low-humidity air with **NaPyr** (0.5 mmol) in THF, and (d) reactions of $CO_{2(g)}$/ambient air/low-humidity air with solid-state **NaCrownPyr** (0.5 mmol) were also performed.

1.3 Reaction of K^{Me}Carbamate with [$(NO)_2$Fe(TMEDA)]. After a 5-mL THF solution of complex $(NO)_2$Fe(TMEDA) (0.046 g, 0.2 mmol) was prepared in a 25-mL Schlenk tube, this THF solution was then transferred to another 25-mL Schlenk tube loaded with 18-crown-6-ether (0.053 g, 0.2 mmol) and **K^{Me}Carbamate** (0.0328 g, 0.2 mmol) via cannula under positive $N_{2(g)}$ at 35 ^{o}C. This mixture solution was stirred for 5 min at 35 ^{o}C and monitored with FT-IR. Shift of IR ν_{NO} absorption peaks from (1697, 1643) to (1723, 1686, 1636) cm^{-1} supported the conversion of complex $(NO)_2$Fe(TMEDA) into complex [K-18-crown-6-ether][$(NO)_2$Fe(^{Me}PyrCO_2)]$^{-}$ (**Fe^{Me}Carbamate**, ^{Me}PyrCO_2 = 3-methylcarbamate).

1.4 Heat:Vacuum-induced Regeneration of M^{R}Pyr from M^{R}Carbamates. After **K^{Me}Carbamate** (164 mg, 1 mmol) was loaded into a 50-mL Schlenk flask under $N_{2(g)}$, **K^{Me}Carbamate** was incubated in a pre-heated oil bath at 175 °C (or 125 °C, 150 °C, and 200 °C) for 30 min (or 60 min), while the Schlenk flask was connected to vacuum. The heat-treated **K^{Me}Carbamate** was collected and analyzed using ^{13}C MAS NMR. Shift of ^{13}C MAS NMR signals from (15.1, 106.6, 131.7, 149.1, 152.1) ppm to (14.9,

102.6, 140.1, 148.0) ppm supported the successful regeneration of **K^{Me}Pyr** from **K^{Me}Carbamate** through a heat:vacuum process. Then, the regenerated **K^{Me}Pyr** was dissolved in d_6-DMSO for quantitative NMR analysis using 1,3,5-trimethoxybenzene as an internal standard (yield 73.7±3.5%, Figure S4). Treatment of **NaCarbamate** with a heat-vacuum process at 175 °C for 30 min and characterization/quantitation of regenerated **NaPyr** (78.5±5.0%) were executed under a similar methodology.

Regenerations of (a) **K^{Me}Pyr** from **K^{Me}Carbamate** (derived from DAC of CO_2 using **K^{Me}Pyr**) and (b) **NaPyr** from **NaCarbamate** (derived from reaction of **NaPyr** with $CO_{2(g)}$ or DAC of CO_2 using **NaPyr**) were performed under a similar heat:vacuum-incubation process and analyzed using ^{1}H NMR/^{13}C MAS NMR.

1.5 Evaluation of the CO_2-capture Reactivity of As-prepared (or Heat:Synthetic Air-treated) M^RPyr under Continuous Flow of Ambient Air/Low-humidity Air/Humidified Air under Different Humidity Levels Using a Direct Air Capture (DAC) System. Design for a direct air capture (DAC) system was depicted in Scheme S2. An air compressor (component 1, REXON, Ol20-9) was used to compress ambient air as the gas source and to generate a flow of ambient air. For the DAC experiments, a flow of ambient air at 0.2 standard liter per minute (SLPM) was set using a mass flow controller (component 2, Alicat scientific, ALI-MC-2SLPM-D). Downstream to this mass flow controller, a CO_2:humidity meter (component 4, Lutron CO2-9914SD) was installed to measure the concentration of CO_2 and to monitor the humidity at the inlet. Then, a sample column (component 5, a gas-tight glass filter tube (O.D. = 25 mm) connected with a 50-mL Schlenk flask) was loaded with the DAC sorbent **M^RPyr** (0.5 mmol) for capturing CO_2 from the flow of ambient air. After exposure of the DAC sorbent **M^RPyr** in the sample column with the flow of ambient air at room temperature, the flow rate for this downstream flow of air at the outlet was monitored using a mass

flow meter (component 6, Alicat scientific, ALI-M-2SLPM-D), in order to confirm the absence of a significant pressure drop. In addition, the concentration of CO_2 in the downstream outlet airstream was monitored using either a CO_2:humidity meter (LI-850, LI-COR; component 7) or a gas chromatography equipped with an online analysis system (Shimadzu GC-2030 fitted with a Shinwa Micropacked-ST column filled with Shincarbon-ST and a barrier discharge ionization detector (BID); component 7). Prior to loading the DAC sorbent **M^{R}Pyr** into the sample column (component 5), the relative humidity level of the ambient air feed was determined to be 20%-40% RH using the CO_2:humidity meter (component 7), reflecting the natural fluctuations of ambient moisture levels driven by prevailing weather and atmospheric conditions. When using gas chromatography for downstream CO_2 concentration analysis, $He_{(g)}$ was adopted as a carrier gas to conduct the GC analyses. The column temperature was set as 40 ^oC and increased to 100 ^oC in 5 min before the automatic injection of the gaseous sample every 7 min through the whole DAC experiments (360 min). Data collected every 7 min were then used to determine the concentration of $CO_{2(g)}$ at the outlet and to depict the breakthrough curves. After each DAC experiment, the difference between the concentration of $CO_{2(g)}$ at the inlet and that at the outlet during each sampling time was further integrated through the whole DAC experiments (360 min). DAC capacity of the **M^{R}Pyr** sorbent (mmol CO_2) were then calculated through multiplying these integrated data ($[CO_2]$•min) with the flow rate of the ambient air at the inlet (0.2 L/min). Through integrating the breakthrough curves for DAC using **M^{R}Pyr**, the adsorption kinetics were analyzed via fitting the CO_2 uptake profiles with pseudo-first-order, pseudo-second-order, and Elovich kinetic models. These fittings were performed using Origin to determine the adsorption rate constants (k_{ad}) and adsorption half-time ($t_{1/2}$).

To evaluate the DAC activity of **K^{Me}Pyr** at different inlet air flow rate (0.2, 1.0,

2.0, and 3.0 L/min), breakthrough curves were obtained through monitoring the outlet CO_2 concentration every second using a CO_2:humidity meter (LI-850, LI-COR). The resulting breakthrough curves were then integrated to obtain CO_2 uptake profiles, which were fitted to the pseudo-first-order kinetic model to determine the adsorption rate constants (k_{ad}) and adsorption half-time ($t_{1/2}$), following the procedure described above.

After the DAC sorbent **NaPyr** (or **K^{Me}Pyr** and **KPyr**) was saturated with $CO_{2(g)}$ from the ambient air using the DAC system described above, the CO_2-capture adduct was isolated and analyzed using solid-state IR, PXRD, and ^{13}C MAS NMR. After the saturation of **K^{Me}Pyr** with CO_2 through exposure to the ambient air using the DAC system described above, extended exposure to a continuous flow of ambient air for 24 h followed by IR spectroscopic characterization was performed to evaluate the stability of the CO_2-captured adduct.

To systematically evaluate the CO_2-capture performance of the DAC sorbents **M^{R}Pyr** (or **K^{Me}Pyr·H$_2$O**) under air feeds across a wide spectrum of relative humidity levels, as depicted in Scheme S2, the gas-conditioning module (component 3) between the mass flow controller (component 2) and the CO_2:humidity meter (component 4) was configured according to the desired operating humidity. To evaluate the CO_2-capture performance under a low-humidity airstream, a drying column filled with anhydrous $CaSO_{4(s)}$ (component 3) was installed, which provided a low-humidity air feed determined to be 5% RH using the CO_2:humidity meter (component 7, LI-850). On the other hand, to generate humidified airstreams of varying humidity, component 3 was replaced with a water bubbler coupled with a temperature-controlled condenser connected to a water chiller. Prior to loading the DAC sorbent **M^{R}Pyr** into the sample column (component 5), the relative humidity levels of the resulting humidified airstreams were verified to be 30% RH, 60% RH, and 100% RH by setting the chiller

temperature to 5 °C, 15 °C, and 25 °C, respectively.

Oxidative stability of **NaPyr** and CO_2-capture performance of heat:synthetic air-treated **NaPyr** under DAC were investigated following the procedure described below. After 50 mg of **NaPyr** was loaded in a 50-mL Schlenk flask under $N_{2(g)}$, this **NaPyr**-loaded flask was incubated in an oil bath at 175 °C and purged with synthetic air (a mixture gas of 21% $O_{2(g)}$ and 79% $N_{2(g)}$, JIAN FANG GASES CO., LTD.) for 24 h. Then, the CO_2-capture performance of heat:synthetic air-treated **NaPyr** under a continuous flow of ambient air using the DAC system was evaluated following the procedure described above. In addition, the CO_2-capture adduct was isolated and analyzed using solid-state IR.

1.6 *Ex Situ* PXRD Monitoring of the CO_2-capture Mechanism of As-prepared K^{Me}Pyr under a Continuous Flow of Ambient Air. Using the DAC system shown in Scheme S2, 120 mg of **K^{Me}Pyr** was loaded into the sample column (component 5) and exposed to a continuous flow of ambient air (20%-40% RH) at a flow rate of 0.4 L/min. After exposure to ambient air for 1 min, the atmosphere of the sample column was immediately replaced with $N_{2(g)}$, and the sample was collected in an $N_{2(g)}$-filled glove box before being analyzed by PXRD. **K^{Me}Pyr** exposed to ambient air for 5 min, 10 min, 30 min, 60 min, and 120 min, respectively, were prepared and analyzed by PXRD following a similar procedure.

1.7 Preparation of Hydrated K^{Me}Pyr ($K^{Me}Pyr \cdot H_2O$). Hydrated **K^{Me}Pyr** (**$K^{Me}Pyr \cdot H_2O$**) was prepared using the modified DAC system shown in Scheme S2. An air compressor (component 1, REXON, Ol20-9) was used to compress ambient air as the gas source and to generate a flow of ambient air, and a flow of ambient air at 0.2 standard liter per minute (SLPM) was set using a mass flow controller (component 2, Alicat scientific, ALI-MC-2SLPM-D). Downstream to this mass flow controller, a

sample column (component 3, a gas-tight glass filter tube (O.D. = 25 mm) connected with a 50-mL Schlenk flask) was loaded with **K^{Me}Pyr** (2.0 mmol) for capture of CO_2 from ambient air and generation of a flow of CO_2-free air ([CO_2] ≤ 31 ppm, RH% = 10-15%), which was confirmed using the downstream CO_2:humidity meter (component 4, Lutron CO2-9914SD). Then, **K^{Me}Pyr** (0.5 mmol) loaded in the sample column (component 5) was exposed to this flow of CO_2-free air for 5 min (or 25 min) before the hydrated **K^{Me}Pyr** was analyzed using PXRD. Based on the PXRD analyses (Figure 6h), **K^{Me}Pyr·H_2O** was successfully prepared through exposure of **K^{Me}Pyr** to CO_2-free air for 25 min, whereas the as-prepared **K^{Me}Pyr·H_2O** was then used for DAC experiment against low-humidity air.

1.8 Recyclability Test of M^{R}Pyr for DAC under a TVSA Process. **K^{Me}Pyr** (240 mg, 2.0 mmol) was loaded into a sample column (a gas-tight glass filter tube (O.D. = 25 mm) under $N_{2(g)}$. Before the DAC experiment, a blank column (without any sample) was installed onto the DAC system to monitor the background CO_2 concentration and relative humidity in the ambient air. A flow of ambient air at 0.4 standard liter per minute (SLPM) was set using a mass flow controller. After 1 h of stable readings, the sample column loaded with **K^{Me}Pyr** was installed onto the DAC system, where ambient air was passed through this sample column to initiate the DAC by **K^{Me}Pyr** yielding **K^{Me}Carbamate**. After saturation of **K^{Me}Pyr** with CO_2 from air, the sample column containing the resulting **K^{Me}Carbamate** (or the CO_2-saturated powder) was transferred to the glovebox, and the powder was transferred into a 50-mL Schlenk flask. This 50-mL Schlenk flask loaded with air-exposed **K^{Me}Pyr** was then heated at 200 °C under vacuum for 1 h. Then, the regenerated **K^{Me}Pyr** was transferred back to the sample column for the next round of DAC experiment. Following the procedure described above, five consecutive cycles for DAC-regeneration process were performed to

evaluate the recyclability of **K^{Me}Pyr** for DAC under a TVSA process. Recyclability of **NaPyr** for DAC under a TVSA process was evaluated under a similar methodology described above.

During the heat-vacuum treatment of **M^{R}Carbamate**, in addition to the dominant regeneration of **M^{R}Pyr**, mechanism for minor degradation of **M^{R}Carbamate** was explored based on the experimental procedure described below. Using an experimental setup illustrated in Scheme S4, **K^{Me}Carbamate** (250 mg) was loaded in a 50-mL Schlenk flask under $N_{2(g)}$. After the **K^{Me}Carbamate** was incubated in an oil bath at 200 ^{o}C under vacuum for 1 h, the residual powder sample in the 50-mL Schlenk flask was characterized using solid-state IR spectroscopy, which revealed the minor formation of K_2CO_3 according to the IR ν_{CO} absorption peaks at ~1397 cm^{-1}. On the other hand, ^{1}H NMR analysis on the products collected in another tube using cold trap supports the release of H_2O from **K^{Me}Carbamate** and conversion of **K^{Me}Carbamate** into 3-methylpyrazole. Based on a similar methodology, the heat-vacuum treatment of **NaCarbamate** resulted in the minor formation of Na_2CO_3 and pyrazole, in addition to the dominant regeneration of **NaPyr**.

1.9 Stability of M^{R}Pyr (or K^{Me}Carbamate) in H_2O and Reactivity of M^{R}Pyr toward CO_2 in H_2O. To evaluate the stability of **K^{Me}Pyr** in liquid water, 10 mg of **K^{Me}Pyr** was loaded in a 25-mL Schlenk tube under $N_{2(g)}$ followed by addition of 1 mL of H_2O using a gas-tight syringe. After this anaerobic aqueous solution was incubated at room temperature overnight, H_2O was removed under vacuum. The obtained crude solid-state product was then dissolved in 0.5 mL of d_6-DMSO before ^{1}H NMR experiment. For comparison, ^{1}H NMR spectra for as-prepared **K^{Me}Pyr** in d_6-DMSO and D_2O, and 3-methylpyrazole in d_6-DMSO and D_2O were also measured. Moreover, stability of **NaPyr** in liquid water was investigated in a similar manner through comparisons of the

^1H NMR spectra for as-prepared **NaPyr** in d_6-DMSO and D_2O, pyrazole in d_6-DMSO and D_2O, and recovered **NaPyr** (derived from aqueous solution of **NaPyr**) in d_6-DMSO.

To a 50-mL Schlenk flask loaded with a 20-mL aqueous solution of **K^{Me}Pyr** (0.5 g, 4.17 mmol), one equiv. of $CO_{2(g)}$ was introduced for ~15 s at a flow rate of 0.4 L/min. Then, the solvent was removed under vacuum before the crude solid-state product was extracted with 30 mL *n*-hexane. After the *n*-hexane solution was transferred to another 50-mL Schlenk flask and the solvent was removed under vacuum, the obtained product was dissolved in 0.5 mL of d_6-DMSO for ^1H NMR analysis. On the other hand, the above-mentioned crude solid-state product, washed with *n*-hexane, was dried under vacuum before its solid-state IR spectrum was measured. Reaction of **NaPyr** and $CO_{2(g)}$ in a 1:1 stoichiometry in H_2O followed by ^1H NMR and IR characterizations were performed in a similar manner.

To evaluate the stability of **K^{Me}Carbamate** in liquid water, 200 mg of **K^{Me}Carbamate** was loaded in a 50-mL Schlenk flask under $N_{2(g)}$ followed by addition of 2 mL of H_2O using a gas-tight syringe. After this anaerobic aqueous solution was incubated at room temperature overnight, H_2O was removed under vacuum before the obtained crude solid-state product was extracted with 30 mL *n*-hexane. After the *n*-hexane solution was transferred to another 50-mL Schlenk flask and the solvent was removed under vacuum, the obtained product was dissolved in 0.5 mL of d_6-DMSO for ^1H NMR analysis. On the other hand, the above-mentioned crude solid-state product, washed with *n*-hexane and THF, was dried under vacuum before its solid-state IR spectrum was measured.

1.10 Crystallography. Each crystal was mounted on a glass fiber and quickly coated in epoxy resin. Unit-cell parameters were obtained by least-squares refinement. Diffraction measurements for compounds **K^{Me}Pyr**, **NaPyr**, **NaCrownPyr**, and

$K^{Me}Pyr•H_2O$ were carried out on a SMART Apex CCD diffractometer with graphite-monochromated Mo K_α radiation (λ = 0.7107 Å) between 8.802˚ and 142.282˚ for compound **$K^{Me}Pyr$**, between 2.25˚ and 25.10˚ for compound **NaPyr**, between 2.09˚ and 25.08˚ for compound **NaCrownPyr**, and between 10.168˚ and 144.91˚ for compound **$K^{Me}Pyr•H_2O$**. Least-squares refinement of the positional and anisotropic thermal parameters of all non-hydrogen atoms and fixed hydrogen atoms was based on F^2. A SADABS absorption correction was made.[3] The SHELXTL structure refinement program was employed.[4] CCDC 2521718 (**$K^{Me}Pyr$**), 2521719 (**$K^{Me}Pyr•H_2O$**), 2521720 (**NaCrownPyr**), and 2521722 (**NaPyr**) contain the supplementary crystallographic data for these structures. These data are provided free of charge by The Cambridge Crystallographic Data Centre.

1.11 Structural Determination of K^{Me}Carbamate. The datasets were collected at HRPXRD beamline TPS 19A of the National Synchrotron Radiation Research Center (NSRRC) using monochromatic X-rays with a wavelength of 0.77489 Å (16 keV). Data collection was performed with an exposure time of 5 s per frame, repeated 20 times with a 30 s dwell time. Peak identification was executed within a 2θ range of 2.4° to 27.4° using an intensity threshold of 1.5%, yielding a total of 21 identified peaks. Unit cell indexing was subsequently carried out in EXPO2 using the N-TREOR09 algorithm, obtaining a monoclinic unit cell with a = 28.421(15) Å, b = 5.928(2) Å, c = 4.078(2) Å, β = 92.72(5)°, and V = 686.3(5) $Å^3$ (figure of merit M_{20} = 13; zero-point shift = 0.000°). The space group was assigned as $P\ 2_1/a$ at beginning and converted to the standard setting $P\ 2_1/c$. After the cell indexing, an entire initial model is needed, the expected molecular geometry of the monoanionic 3-methyl-pyrazole-1-carboxylate moiety ($^{Me}PyrCO_2$) was optimized at the density functional theory (DFT) level using the ORCA software package (version 6.1.1).[5] The calculations were conducted with the composite

r2SCAN-3c electronic structure method, and the def2-mTZVP/J auxiliary basis set was employed to enable Coulomb fitting under the Resolution of Identity (RI) approximation. The DFT-optimized $^{Me}PyrCO_2$ geometry and potassium (K) atoms were subsequently utilized as initial structural fragments for ab initio powder structure solution via simulated annealing in the EXPO2 program.[6] Due to data statistics, the HRPXRD dataset was restricted to a maximum 2θ angle of 27.4°, whereby a maximum d-spacing resolution of 1.636 Å and a total of 169 reflections were obtained. The initial crystal structural model generated from global optimization is displayed in Figure S28, yielding a cost function value of 17. At this point, the indexed unit cell volume of 686.3 $Å^3$ accommodated four [K][$^{Me}PyrCO_2$] (**K^{Me}Carbamate**) formula motifs (Z = 4), matching space group $P\ 2_1/c$ and without any packing solvent (e.g., H_2O).

This initial crystal **K^{Me}Carbamate** model was imported into the GSAS-II software package,[7] where full-matrix *Rietveld* refinement was carried out against the experimental HRPXRD pattern to determine the final crystal structure. Before the structure refinement, large isotropic displacement parameters (U_{iso}) for the C, N, O, and H atoms were observed during simulated annealing (SA), indicating potential structural disorder within the ligand. During the refinement process, the U_{iso} values of all non-hydrogen ligand atoms (C, N, and O) were constrained to be equivalent, whereas those of the hydrogen atoms were fixed at twice this value. The thermal parameter of the K atom was refined freely. To preserve realistic molecular geometry during refinement, bond distance restraints were established using target values derived directly from the DFT-optimized structure. The bond lengths within the carboxylate group were restrained to 1.226 Å (C1–O1), 1.238 Å (C1–O2), and 1.563 Å (C1–N1). For the heterocyclic ring and its methyl substituent, restraints were assigned to N1–N2 (1.347 Å), N2–C2 (1.338 Å), C2–C3 (1.413 Å), C3–C4 (1.382 Å), C4–N1 (1.355 Å), and C2–

C5 (1.496 Å) with a weighting parameter of 0.033. C–H bond restraints were specified as 1.080 Å for the aromatic hydrogen atoms (C3–H4 and C4–H5) and 1.096 Å for the methyl hydrogen atoms (C5–H1, C5–H2, and C5–H3) with a weighting parameter of 0.0033. Furthermore, planar restraints were imposed on two distinct structural units, the carboxylate group (C1, O1, O2, N1) and the organic framework (C1, N1, N2, C2, C3, C4, C5), that enforced coplanarity across the ligand backbone. Conceptually, the monoanionic $[^{Me}PyrCO_2]^-$ ligand and the potassium cation were treated as two independent structural fragments, allowing controlled translational and rotational motion of the entire ligand relative to the metal cation within the specified restraint tolerances to complete the refinement of fractional coordinates. The fractional coordinates of the K atom were refined freely. Preliminary result of structural refinement with these restraints yielded an unphysical short intermolecular contact of approximately 1.82 Å between H3 and H5 of neighboring ligands upon crystal packing. To eliminate this steric conflict, the methyl group on the ligand was rotated to stagger the hydrogen positions.

Following full Rietveld refinement, the data were refined over a 2θ range up to 64°. The final unit cell parameters were determined to be a = 4.07466(18) Å, b = 5.9243(5) Å, c = 28.3952(28) Å, β = 92.755(6)°, and V = 684.66(8) Å^3 with $P\ 2_1/c$ space group. The detailed crystallographic data and experimental refinement parameters are summarized in Table S1. Microstructure refinement indicates pronounced anisotropy in both crystallite size and microstrain as shown in Figure S29. Analysis of domain size yielded an equatorial dimension of 0.205(19) μm and an axial dimension fixed at 1.00 μm along the [001] unique axis. The corresponding 3D microstrain tensor in Figure S29a displays an oblate distribution along the (100) axis, visually confirming significant strain anisotropy where the equatorial microstrain is

approximately twice as large as the axial strain. As depicted in the Figure S29b of 3D crystallite size surface, this morphology is characterized by a highly elongated ellipsoid along (001), reflecting fine and needle-like crystallites stacked along the *c*-axis that are consistent with preferred 2D sheet-like stacking growth. Furthermore, microstrain ($\Delta d/d$) analysis along the [100] unique axis yielded 0.0095(3) for the axial component and 0.0177(1) for the equatorial component. The experimental, calculated, and difference patterns obtained from the final Rietveld refinement are shown in Figure 5a. The final refined crystal structure and its packing models are presented in Figures 5b-5c. Detailed atomic coordinates, isotropic displacement parameters, bond distances, and bond angles are listed in Tables S2 and S3. CCDC 2586973 (**K^{Me}Carbamate**) contains the supplementary crystallographic data for this structure. This data is provided free of charge from CCDC. These data are provided free of charge by The Cambridge Crystallographic Data Centre.

1.12 Exposure of NaPyr to a Continuous Flow of Ambient/Low-humidity Air Monitored using *In Situ* Diffuse Reflectance Infrared Fourier Transform (DRIFT) Spectroscopy. *In situ* DRIFTS measurements were conducted using a Nicolet iS10 FT-IR spectrometer (Thermo Scientific) equipped with a liquid-nitrogen-cooled mercury-cadmium-telluride (MCT) detector and a Praying Mantis™ diffuse reflectance reaction chamber (Harrick Scientific). The gas delivery manifold was adapted from the DAC system (Scheme S2) by replacing the sample column with the DRIFTS reaction chamber equipped with zinc selenide (ZnSe) windows. To investigate the moisture-gated mechanism, the **NaPyr** sample was exposed to a continuous flow of either ambient air or low-humidity air, whereas the low-humidity air was generated by passing the ambient air stream through a column of anhydrous $CaSO_4$ prior to entering the reaction chamber. Spectra were typically acquired over the 1950-1450 cm^{-1} region.

1.13 Preparation of [18-crown-6-ether-Na][pyrazolate] (NaCrownPyr). NaPyr (0.9 g, 10 mmol) and 18-crown-6-ether (2.773 g, 10.5 mmol) were loaded into a 100-mL Schlenk flask before 50 mL of THF was added under $N_{2(g)}$ at ambient temperature. After this reaction solution was stirred at ambient temperature for 12 h, this reaction solution was concentrated before addition of diethyl ether (30 mL), which led to the precipitation of the white **NaCrownPyr**. Then, the supernatant solution was removed via cannula under positive $N_{2(g)}$, and the insoluble **NaCrownPyr** was dried under vacuum. Crystals suitable for X-ray diffraction were obtained by layering the THF solution of **NaCrownPyr** with hexane at -20 °C for one week.

1.14 Gas Adsorption Isotherm Measurements and Thermodynamic Analysis. Nitrogen (N_2), water (H_2O), and carbon dioxide (CO_2) adsorption isotherms were recorded using Micromeritics ASAP 2020A or 2024 adsorption analyzer. Prior to analysis, all the samples including **NaPyr**, **KPyr**, **K^{Me}Pyr** and **K^{Me}Pyr•H$_2$O** (100 mg), were dried and stored within a glovebox under an $N_{2(g)}$ atmosphere. N_2 adsorption isotherms for **NaPyr**, **KPyr**, **K^{Me}Pyr**, and **K^{Me}Pyr•H$_2$O** were collected at 77 K, whereas H_2O and CO_2 adsorption isotherms for **NaPyr**, **KPyr**, and **K^{Me}Pyr** were collected at 298 K. To obtain the differential enthalpy (ΔH_{ads}) of CO_2 adsorption for **K^{Me}Pyr**, CO_2 adsorption isotherms of **K^{Me}Pyr** were also measured at 273 K and 288 K, respectively.

2. Supplementary Results and Discussion

2.1 Single-crystal X-ray Diffraction Analysis of NaPyr. As shown in Figure 3a, each Na^+ featured a *pseudo*-tetrahedral geometry through coordination with (a) two pyrazolate ligands in a η^1 binding mode ($Na–N_{Pyr}$ bond distance = 2.431(3) Å) and (b) two pyrazolate ligands in a η^2 binding mode ($Na–N_{Pyr}$ bond distances = 2.659(3) Å and 2.508(3) Å). Relying on the coordinating reactivity of adjacent N_2 moiety in pyrazolate, moreover, each pyrazolate served as an organic linker to bridge four Na^+ ions via two η^1 and two η^2 interactions (Figure 3b). These $Na–N_{Pyr}$ interactions in the $[Na(\eta^1\text{-}Pyr)_2(\eta^2\text{-}Pyr)_2]$ and $[Na_4(\mu^4\text{-}Pyr)]$ units resulted in the formation of **NaPyr** as a two-dimensional coordination polymer with a grid-like structure expanding along with the a-c plane (Figure 3c). Furthermore, compact stacking of the two-dimensional and grid-like sheet completed the single-crystal structure of **NaPyr**.

2.2 *In Situ* DRIFTS Monitoring of NaPyr Reactivity toward Ambient versus Low-humidity Air. Among the **M^{R}Pyr** explored in this study, **NaPyr** featured the most distinctive CO_2-capture activity under a continuous flow of low-humidity (5% RH) vs. ambient air (20%-40% RH), namely, absent CO_2-capture activity of **NaPyr** under a continuous flow of low-humidity air. Therefore, mechanism for moisture-gated capture of CO_2 directly from ambient air using **NaPyr** was further investigated using *in situ* diffuse reflectance infrared Fourier transform spectroscopy (DRIFTS). As shown in Figure S22a, time-dependent increase of IR ν_{CO} stretching peak at 1709 cm^{-1} suggested conversion of **NaPyr** into **NaCarbamate** under a continuous flow of ambient air, which was in contrast to the absent formation of **NaCarbamate** under low-humidity air (Figure S22b). Moreover, the CO_2-capture adduct isolated after DAC using **NaPyr** displayed (a) an IR ν_{CO} absorption peak at 1716 cm^{-1}, (b) a distinct PXRD pattern, and (c) characteristic ^{13}C MAS NMR signals (Figure S8). Notably, these spectroscopic

features of the **NaCarbamate** derived from exposure of **NaPyr** to a continuous flow of ambient air are consistent with those prepared through reactions of **NaPyr** with dry/humid $CO_{2(g)}$, and are distinctive from those of $NaHCO_3$ and Na_2CO_3 (Figures S3 and S8).

2.3 Reactivity of NaPyr and NaCrownPyr toward Low-humidity Air, Ambient Air, and $CO_{2(g)}$. In the attempt to unravel the mechanism for moisture-gated capture of CO_2 from ambient air by **M^RPyr**, reaction of **NaPyr** with low-humidity air, ambient air, and $CO_{2(g)}$, respectively, in THF were further investigated. When **NaPyr** was dissolved in THF, injection of low-humidity air resulted in the precipitation of **NaCarbamate** as a white solid (Scheme S1c), which exhibited solid-state IR ν_{CO} stretching frequency at 1716 cm^{-1} (Figure S23). This formation of **NaCarbamate** was consistent with reactions of solid-state **NaPyr** with ambient air (or $CO_{2(g)}$, Scheme 1b, 1e, and S1a).

Through reaction of pyrazole and Na in the presence of 18-crown-6-ether, a molecular complex ([(Na-18-crown-6-ether)(Na)][Pyr]$_2$)$_2$ (**NaCrownPyr**) was successfully prepared and characterized using single-crystal X-ray diffraction and PXRD (Figure S24). Upon treatment of low-humidity air/ambient air/$CO_{2(g)}$ to the solid-state **NaCrownPyr**, formation of distinctive IR ν_{CO} stretching peak at 1714 cm^{-1} indicated the capture of CO_2 yielding the proposed [Na-18-crown-6-ether][Carbamate] (**NaCrownCarbamate**, Figure S25 and Scheme S1d). Based on the results discussed above, effective CO_2-capture reactivity was observed upon reactions of (a) dissolved **NaPyr** (in THF) with low-humidity air/ambient air/$CO_{2(g)}$, (b) solid-state **NaCrownPyr** with low-humidity air/ambient air/$CO_{2(g)}$, and (c) solid-state **NaPyr** with ambient air/$CO_{2(g)}$ (Scheme S1). In contrast, solid-state **NaPyr** displayed absent CO_2-capture reactivity toward low-humidity air. Presumably, (a) dissolution of **NaPyr** in THF and (b) inclusion of 18-crown-6-ether in **NaCrownPyr** may promote the chelation

of the countercation Na^+ within the solid-state **NaPyr** coordination polymer by THF and 18-crown-6-ether, respectively. Moreover, this chelation of the countercation Na^+ triggered the conversion of solid-state **NaPyr** coordination polymer into molecular complexes and recovered the nucleophilic reactivity of dissociated pyrazolate for direct capture of low concentration CO_2 even from low-humidity air.

2.4 Correlating N_2 and CO_2 Adsorption Isotherms and H_2O Adsorption-Desorption Isotherms with the Moisture-Gated DAC Mechanism of M^RPyr. As illustrated in Figures S26a-S26c, the as-prepared **NaPyr**, **KPyr**, **K^{Me}Pyr**, and hydrated **K^{Me}Pyr•H_2O** exhibit negligible uptake in the N_2 adsorption isotherms, which support the non-porous nature of these coordination polymers. Single-component H_2O adsorption and desorption isotherms for **NaPyr**, **KPyr**, and **K^{Me}Pyr** collected at 298 K are depicted in Figures S26d-S26f. Despite the subtle adsorption of water by **K^{Me}Pyr** at 1%-5% RH, all the investigated **M^RPyr** coordination polymers display a step-like gate-opening behavior at thresholds of approximately 7% for **K^{Me}Pyr**, 13% for **NaPyr**, and 22% for **KPyr**, respectively. These step-like gate-opening behaviors of **M^RPyr** are consistent with the moisture-gated **M^RPyr → M^RPyr•H_2O** transformation mechanism. Conversely, desorption of H_2O from the hydrated **M^RPyr**, initiated by decreasing the RH from 92%, proceeded until it stalled at approximately 10% (**K^{Me}Pyr**), 14% (**NaPyr**), and 20% (**KPyr**), respectively, closely matching the RH thresholds at which the corresponding gate-opening transitions were observed during adsorption. Notably, this stalled desorption near the same RH threshold at which the moisture-gated **M^RPyr → M^RPyr•H_2O** transformation occurs may implicate that conversion of **M^RPyr•H_2O** back to **M^RPyr** is hindered under a static and single-component H_2O atmosphere at 298 K. Nevertheless, despite this hindered dehydration under ambient and single-component humidity conditions, of interest, trace CO_2 in air is sufficient to trigger the

release of H_2O from **K^{Me}Pyr•H_2O**, yielding **K^{Me}Carbamate** as the ultimate CO_2-capture product. Taken together, these single-component H_2O adsorption and desorption experiments provide additional evidence for the sequential moisture-gated mechanism "**K^{Me}Pyr → K^{Me}Pyr•H_2O → K^{Me}Carbamate**" discussed in the "**Mechanistic Investigation on Moisture-gated Capture of CO_2 Directly from Air using M^RPyr Coordination Polymers**" section of the main text.

Building on these findings, the correlation between water-uptake behavior and DAC reactivity was also discussed. As shown in Figures 7a-7e, the **M^RPyr** coordination polymers feature negligible DAC reactivity under a low-humidity air stream (5% RH), yet undergo moisture-gated activation for the capture of CO_2 from air when the relative humidity is elevated higher than 20% RH. Taken together, this correlation between the water-uptake property and moisture-gated DAC activity of **M^RPyr** coordination polymers supports that gaseous H_2O molecules from atmospheric moisture function as an allosteric switch, not as a competitive inhibitor, to gate the structural rearrangement of **M^RPyr** coordination polymers and to initiate the capture of CO_2 directly from air.

As shown in Figure S27a, the gravimetric CO_2 adsorption profiles of **K^{Me}Pyr** collected at 273 K, 288 K, and 298 K reveal a steep uptake in the low-pressure region, yielding maximum CO_2-capture capacities of 8.29 mmol/g at 273 K, 6.86 mmol/g at 288 K, and 5.92 mmol/g at 298 K under 1 atm of CO_2. These isotherms confirm that the anionic pyrazolate motifs function as nucleophilic active sites for the chemisorption of CO_2 despite the initially non-porous nature of the **M^RPyr** coordination polymer. After fitting each isotherm to the Langmuir equation,[8, 9] the differential enthalpy (ΔH_{ads}) of CO_2 adsorption for **K^{Me}Pyr** was determined using the Clausius-Clapeyron equation:

$$[(\ln P)_q = \left(\frac{\Delta H_{ads}}{R}\right)\left(\frac{1}{T}\right) + \frac{\Delta S_{ads}}{R}\,]$$

where P is the equilibrium pressure, T is the absolute temperature, and R is the universal

gas constant. By plotting ln P versus 1/T at a fixed CO_2 uptake of 1 mmol/g, the ΔH_{ads} of CO_2 adsorption for **K^{Me}Pyr** was calculated to be -78.9 kJ/mol-sorbent (Figure S27b and Table 1).

The gravimetric CO_2 adsorption profiles of **NaPyr** and **KPyr** collected at 298 K were depicted in Figures S27c-S27d. In contrast to **K^{Me}Pyr**, **KPyr** displays negligible CO_2 uptake across the entire pressure range from low- to high-pressure regions under dry and single-component conditions, whereas **NaPyr** exhibits negligible CO_2 uptake in the low-pressure domain followed by a step-like gate-opening behavior at elevated CO_2 partial pressures. Of interest, these distinct single-component CO_2 adsorption behaviors observed among **K^{Me}Pyr**, **KPyr**, and **NaPyr** may mirror their respective H_2O adsorption isotherms (Figures S26d-S26f). Namely, despite the non-porous nature of the **M^RPyr** coordination polymer, **K^{Me}Pyr** features continuous and smooth CO_2 uptake profiles across variable pressures, whereas **NaPyr** demonstrates a characteristic step-like gate-opening phenomenon at higher partial pressures. On the other hand, regarding the efficient DAC activities of **NaPyr** and **KPyr** under ambient air (Figures 7a and 7c) and their water-uptake characteristics (Figures S26d-S26e), these single-component CO_2 adsorption isotherms unravel the indispensable role of atmospheric moisture in facilitating the moisture-gated activation of CO_2 capture at trace CO_2 concentration.

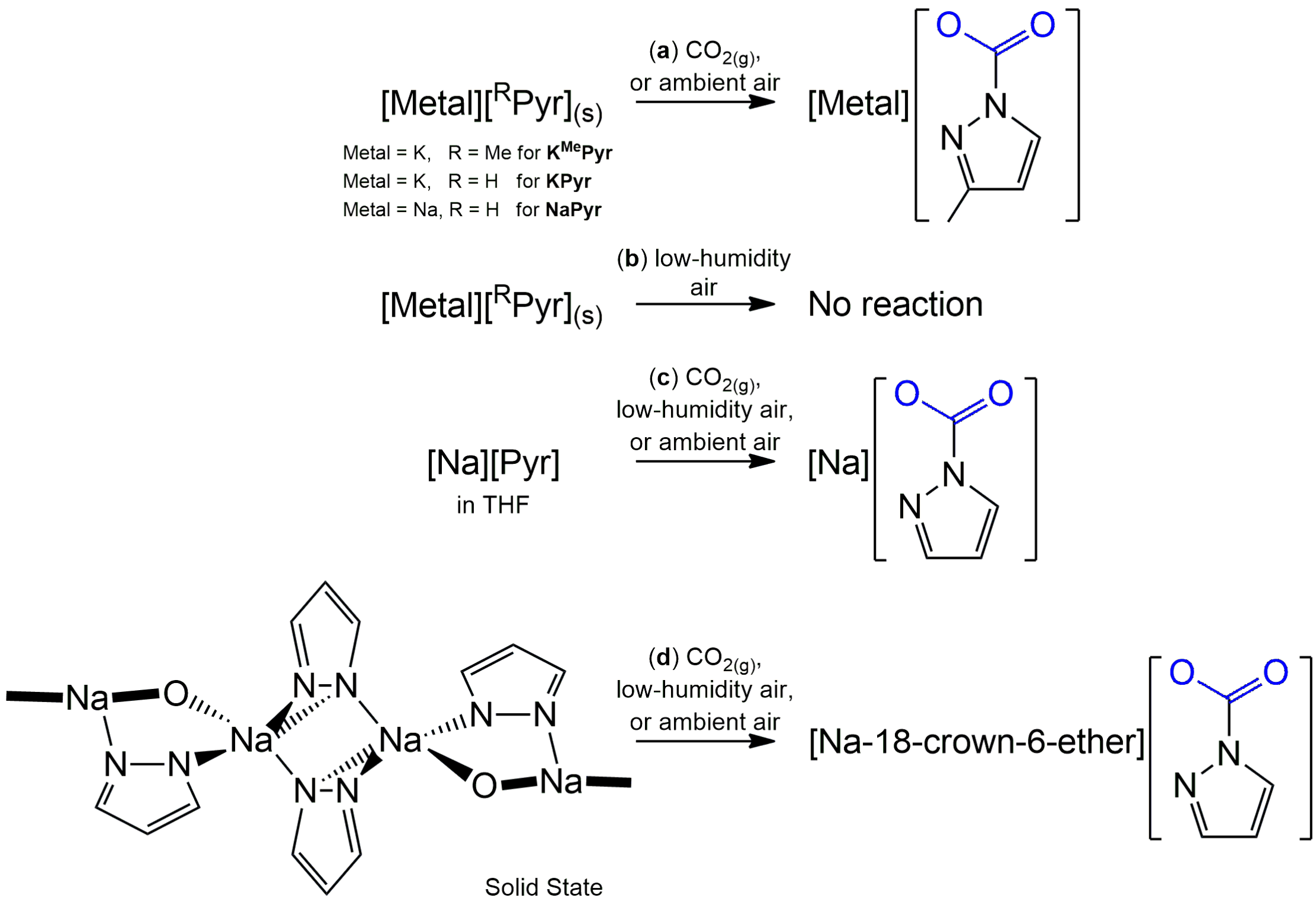

Scheme S1. Comparisons of the reactivities of **M^{R}Pyr** with CO_2 from different sources under alternative conditions.

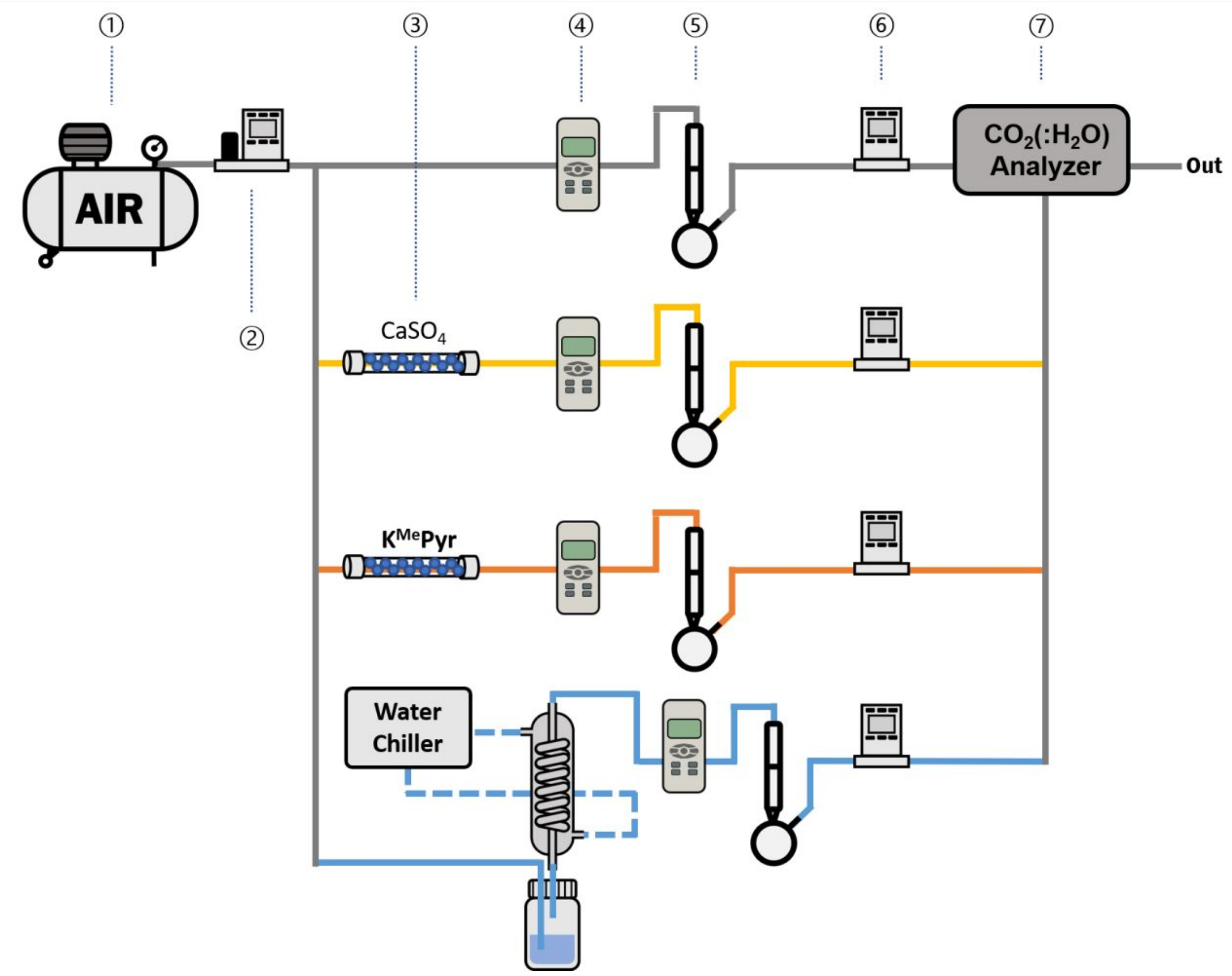

Scheme S2. Schematic illustration of the experimental setup of a DAC system for evaluating the CO_2-capture capacity and mechanism of **M^{R}Pyr** under a continuous flow of ambient (gray)/low-humidity (gold)/humidified (blue) air or preparing hydrated **K^{Me}Pyr** (orange). Component 1: air compressor; component 2: mass flow controller; component 3: column filled with anhydrous $CaSO_{4(s)}$ for low-humidity air (yellow), or column filled with **K^{Me}Pyr** for preparing hydrated **K^{Me}Pyr**, or bubbler with condenser at specified temperature for humidified air; component 4: CO_2 and humidity meter; component 5: sample column for loading of **M^{R}Pyr** (or hydrated **K^{Me}Pyr**); component 6: mass flow meter; and component 7: gas chromatography equipped with an online analysis system or LI-850 (LI-COR). For component 3, the column filled with anhydrous $CaSO_{4(s)}$ was included during the evaluation of the DAC performance of **M^{R}Pyr** under low-humidity air, the column filled with **K^{Me}Pyr** was included during the preparation of the hydrated **K^{Me}Pyr**, and the bubbler with condenser at specified temperature was included during the evaluation of the DAC performance of **M^{R}Pyr** under humidified air.

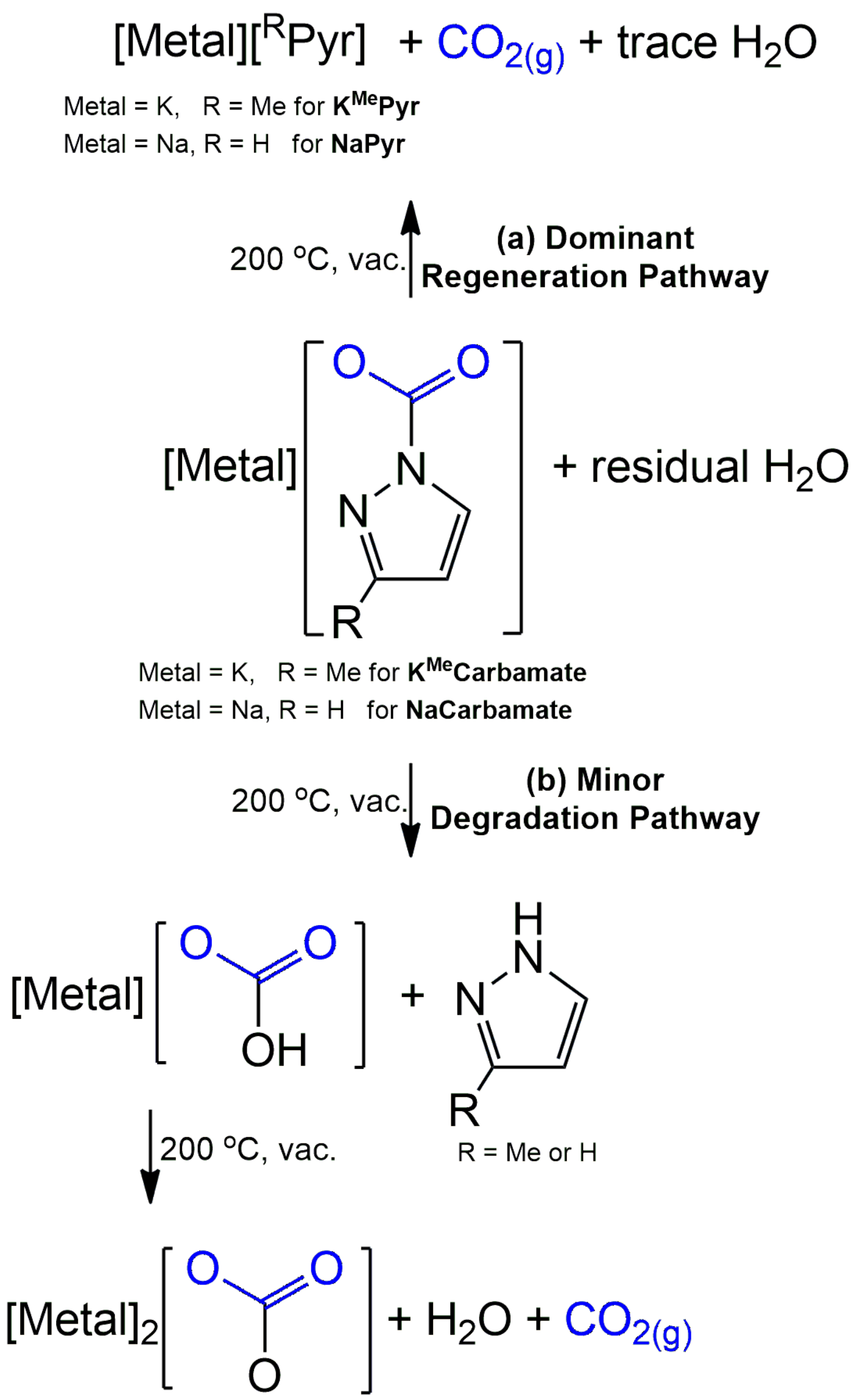

Scheme S3. Proposed mechanisms for heat:vacuum induced (**a**) dominant regeneration of **M^{R}Pyr** from **M^{R}Carbamate** and (**b**) minor degradation of **M^{R}Carbamate** into (3-methyl)pyrazole and M_2CO_3 (M = K or Na, R = Me or H).

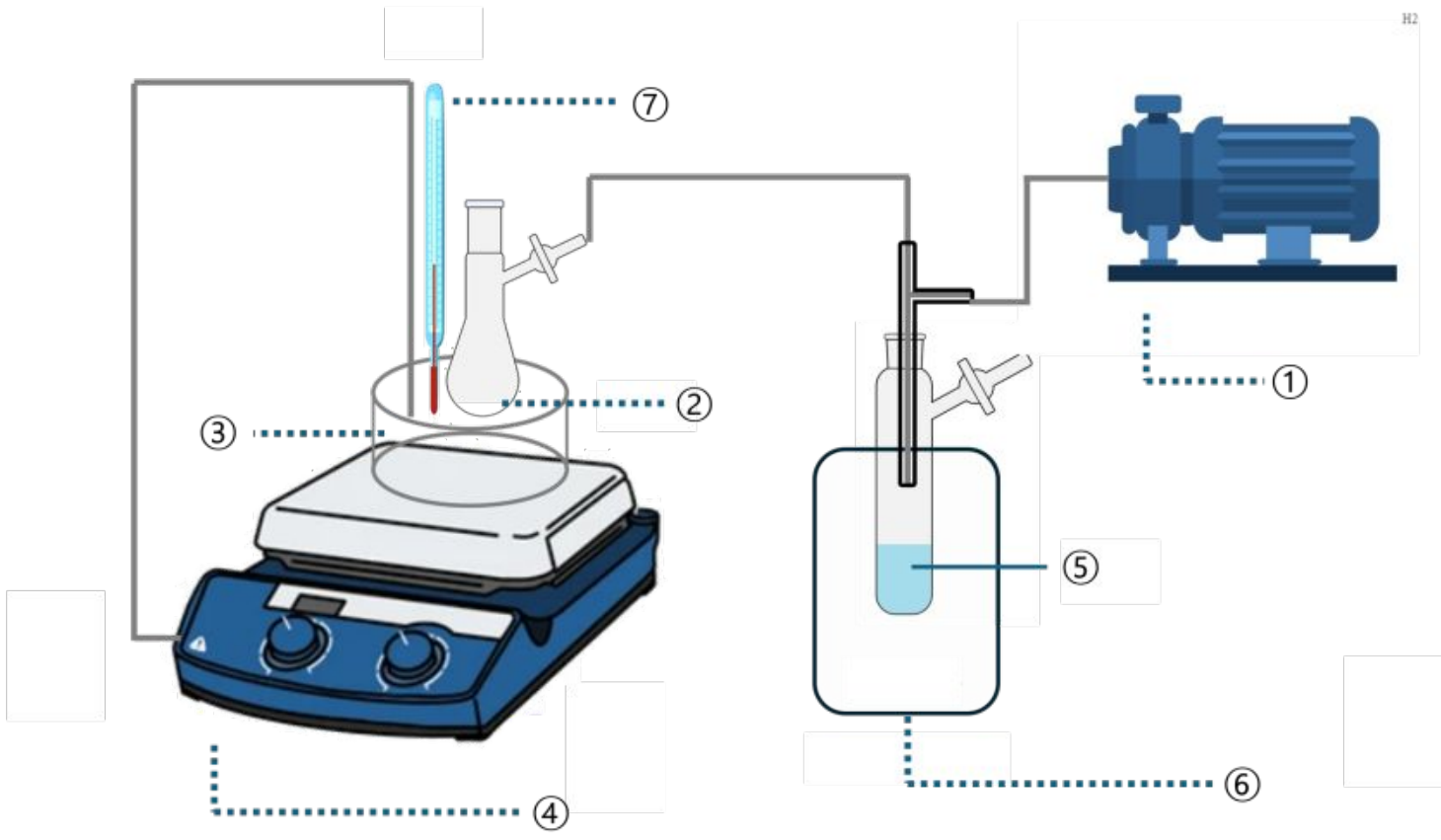

Scheme S4. Schematic illustration of the experimental setup for heat-vacuum treatment of DAC-derived **MR Carbamate**, regeneration of **MR Pyr**, and isolation of evaporated products (i.e. H_2O and (3-methyl)pyrazole). Component 1: vacuum pump, component 2: flask loaded with DAC-derived **MR Carbamate**, component 3: oil bath, component 4: hot plate, component 5: isolation of evaporated products, component 6: Dewar with $N_{2(l)}$ bath, and component 7: thermometer

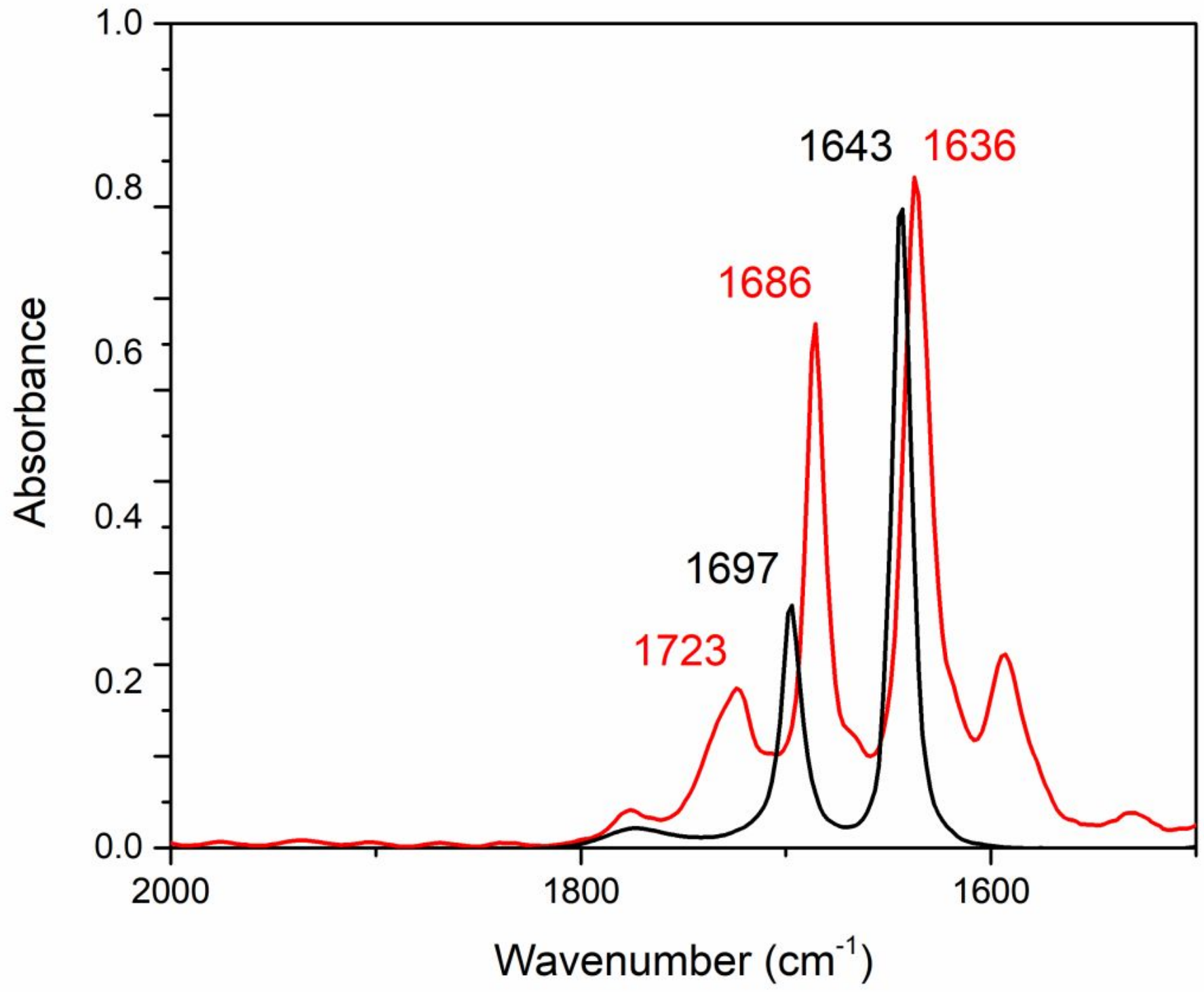

Figure S1. IR spectra for complex [$(NO)_2Fe$(TMEDA)] before (black) and after reaction with **K^{Me}Carbamate** and 18-crown-6-ether in THF (red), which indicated the formation of complex [K-18-crown-6-ether][$(NO)_2Fe$(^{Me}PyrCO_2)] (**Fe^{Me}Carbamate**, ^{Me}PyrCO_2 = 3-methylcarbamate).[10]

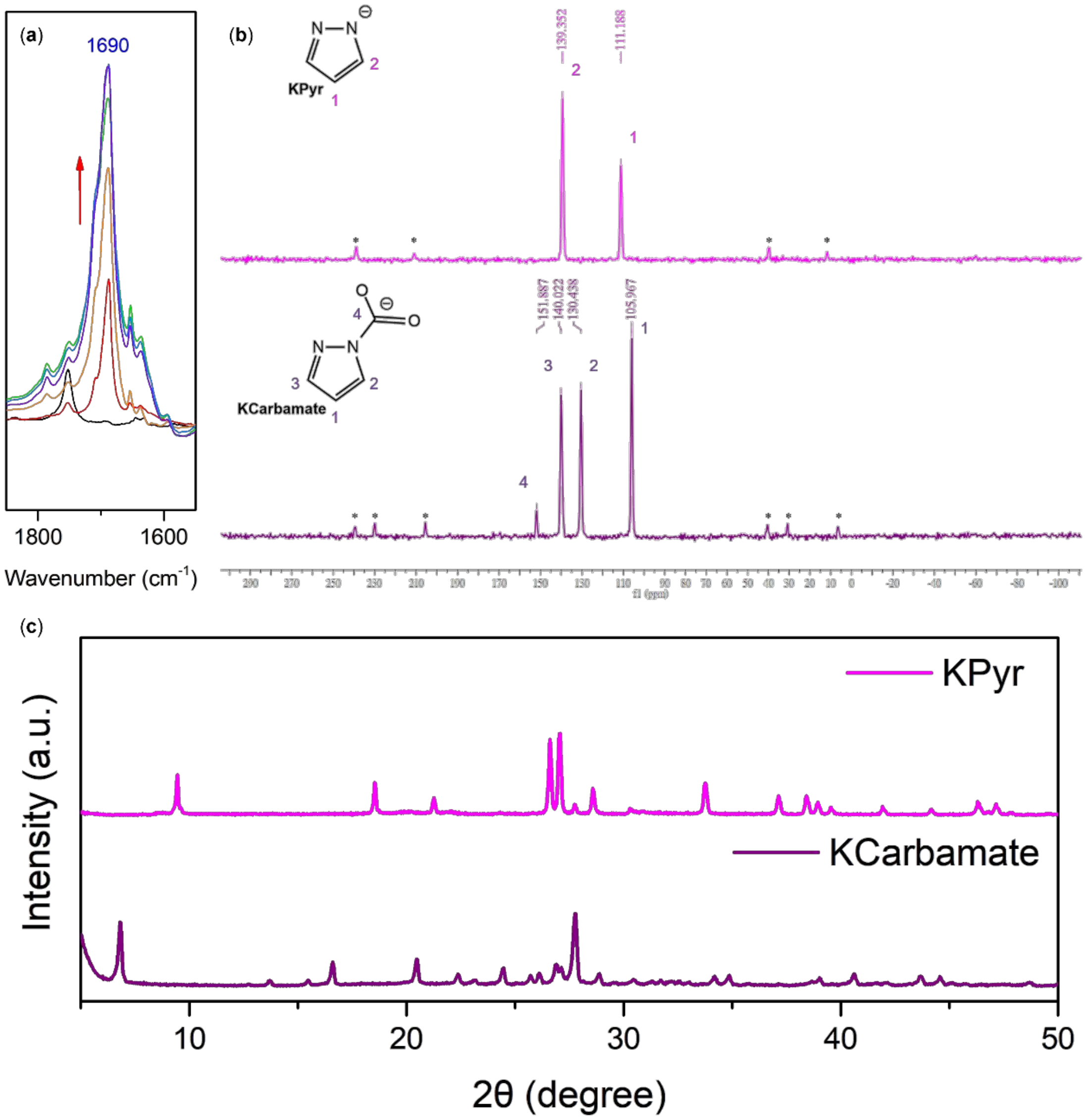

Figure S2. (**a**) Solid-state IR spectra for **KPyr** before (black) and after its reaction with 0.25 (red), 0.5 (orange), 0.75 (green), 1.0 (blue), and 1.25 (purple) equiv. of $CO_{2(g)}$. (**b**) ^{13}C MAS NMR spectra and (**c**) PXRD patterns for **KPyr** before (magenta) and after reaction with stoichiometric $CO_{2(g)}$ (purple). *: spinning side bands.

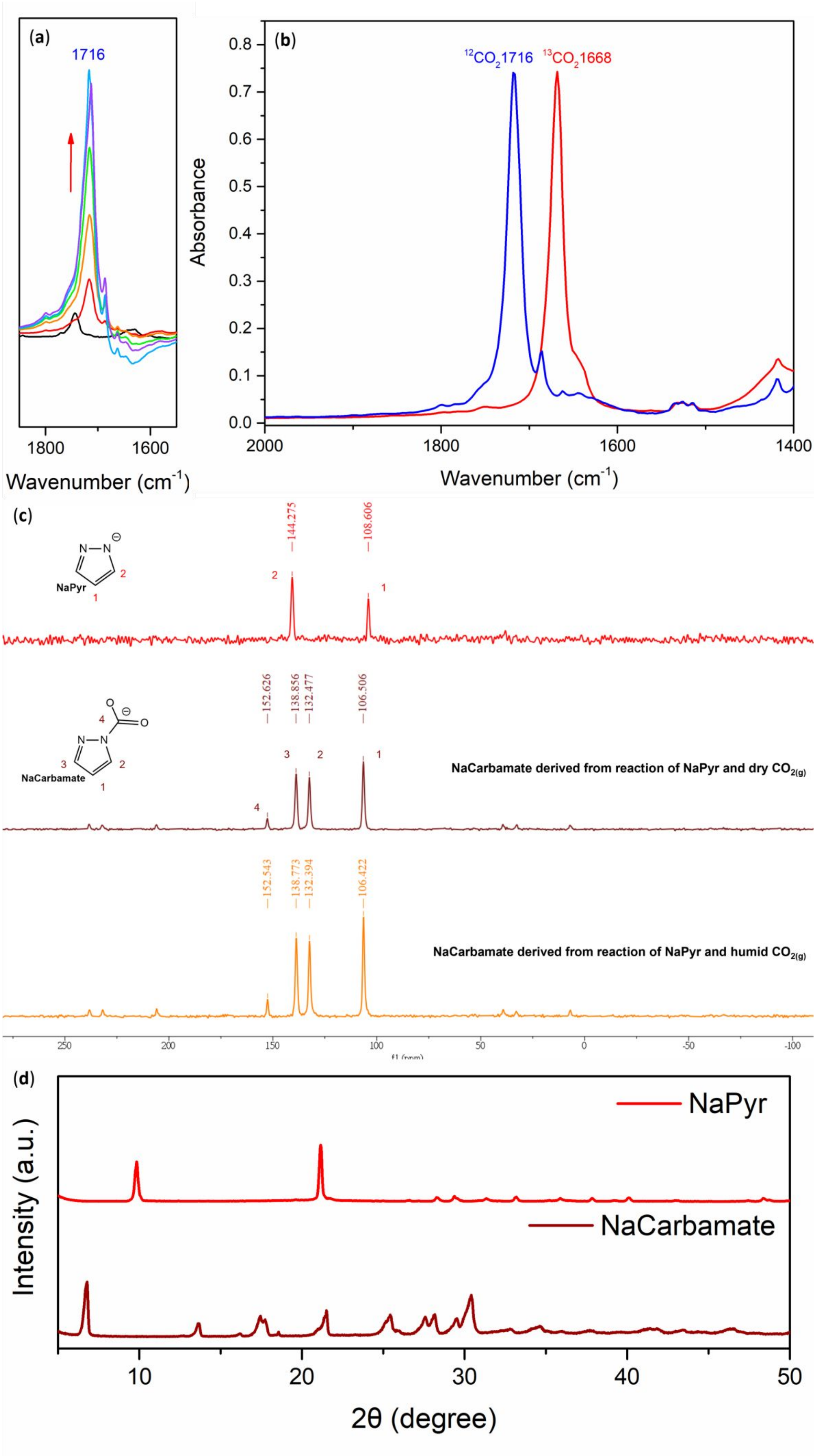
(a)
1716
Wavenumber (cm-1)
(b)
12CO2 1716
13CO2 1668
Absorbance
Wavenumber (cm-1)
(c)
144.275
108.606
NaPyr
152.626
138.856
132.477
106.506
NaCarbamate
NaCarbamate derived from reaction of NaPyr and dry CO2(g)
152.543
138.773
132.394
106.422
NaCarbamate derived from reaction of NaPyr and humid CO2(g)
f1 (ppm)
(d)
NaPyr
NaCarbamate
Intensity (a.u.)
2θ (degree)

Figure S3. (**a**) Solid-state IR spectra for **NaPyr** before (black) and after its reaction with 0.25 (red), 0.5 (orange), 0.75 (green), 1.0 (blue), and 1.25 (purple) equiv. of $CO_{2(g)}$. (**b**) Solid-state IR spectra for **NaPyr** treated with 1.0 equiv. of $CO_{2(g)}$ (blue) and $^{13}CO_{2(g)}$ (red). (**c**) ^{13}C MAS NMR spectra and (**d**) PXRD patterns for **NaPyr** before (red) and after reaction with stoichiometric and dry $CO_{2(g)}$ (dark red). *: spinning side bands. ^{13}C MAS NMR spectrum for **NaCarbamate** derived from reaction of **NaPyr** and humid $CO_{2(g)}$ was depicted in orange, which is comparable to that for **NaCarbamate** derived from reaction of **NaPyr** and dry $CO_{2(g)}$ (dark red).

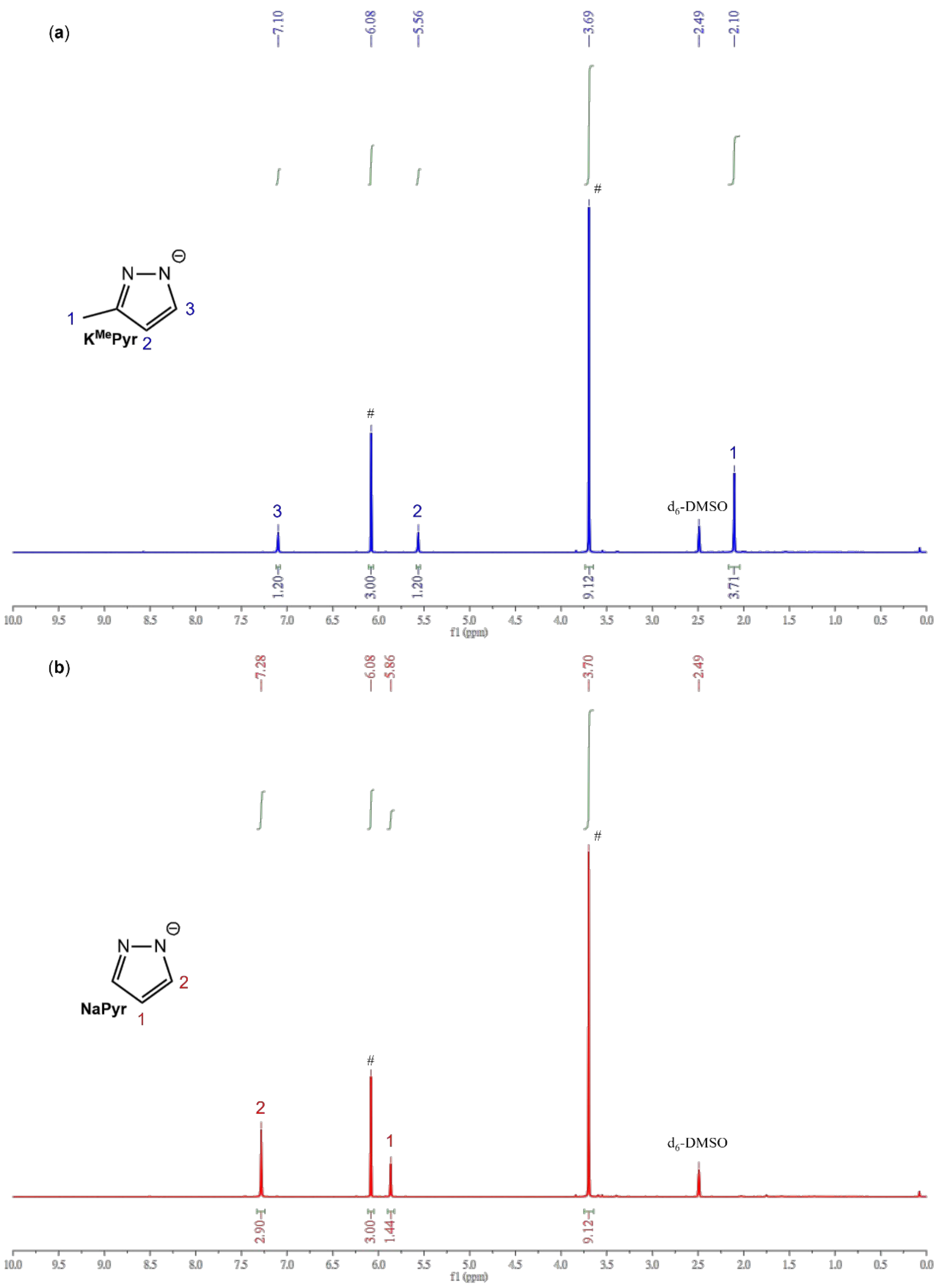

Figure S4. ^{1}H NMR spectra of regenerated (**a**) **K^{Me}Pyr** and (**b**) **NaPyr** in d_6-DMSO derived from incubation of **K^{Me}Carbamate** and **NaCarbamate** under vacuum at 175 °C for 0.5 h. Trimethoxybenzene was used as an internal standard (marked with #).

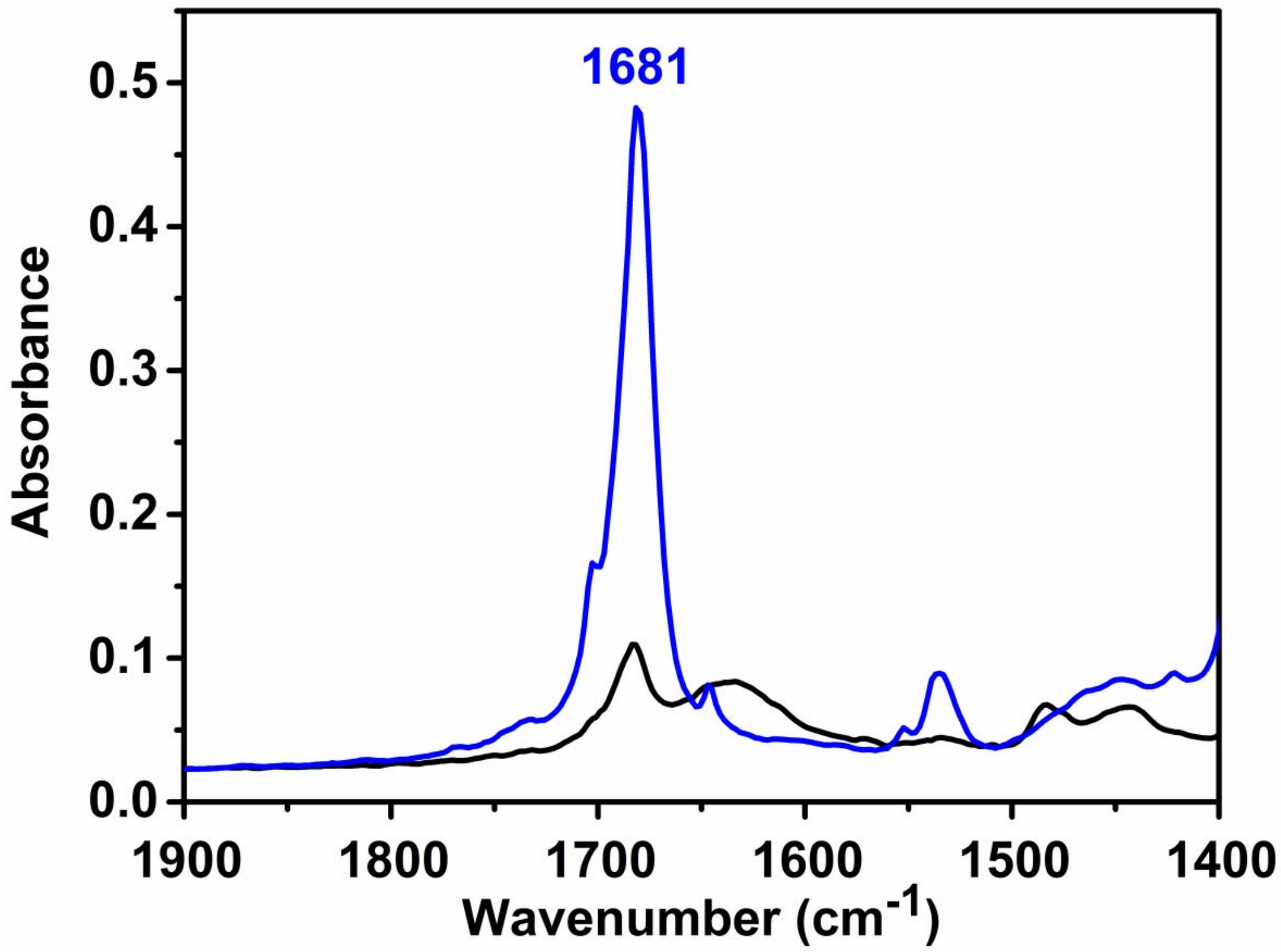

Figure S5. Solid-state IR spectra for **K^{Me}Pyr** treated with low-humidity air (black) and ambient air (blue).

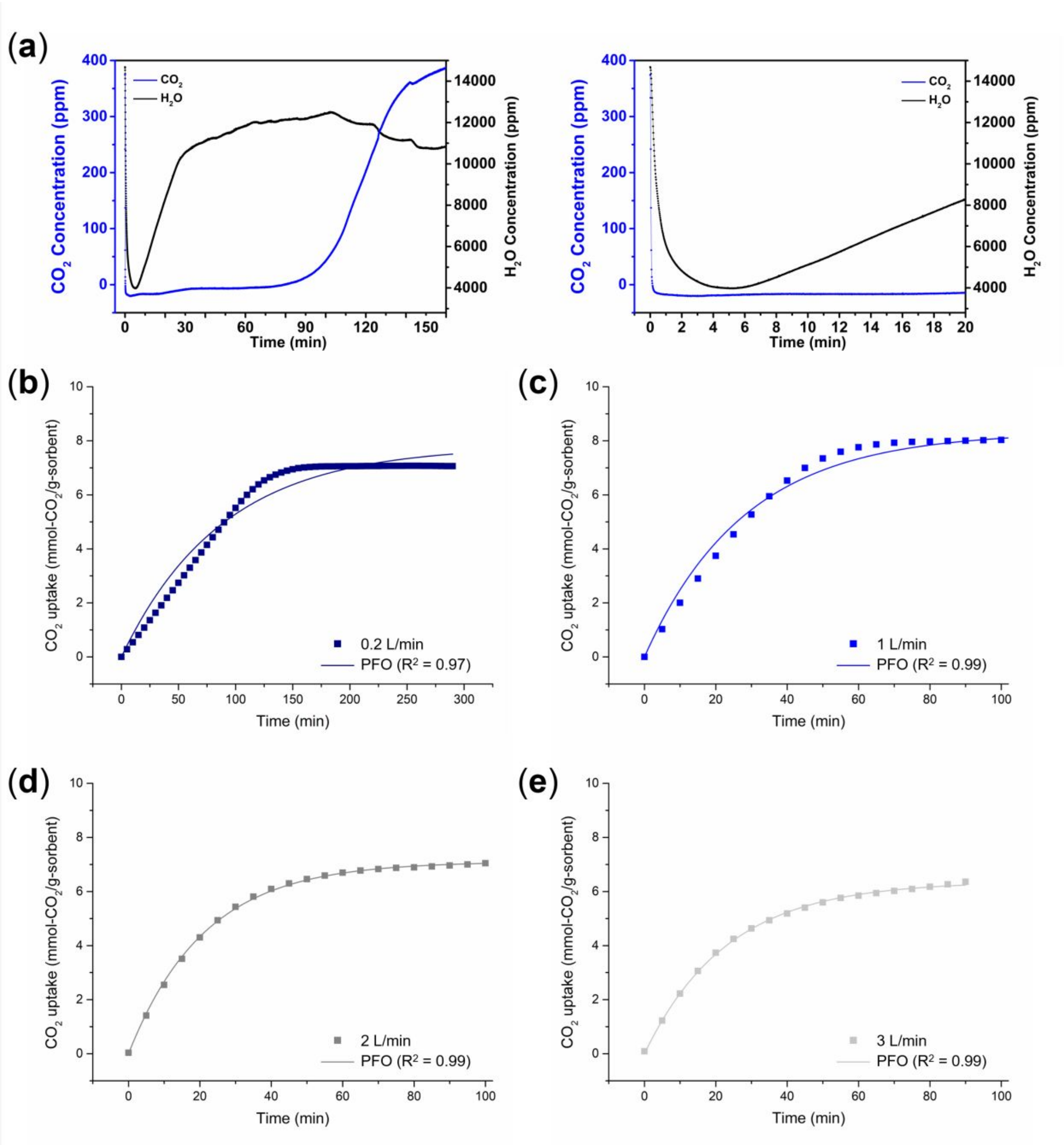

Figure S6. (**a**) Representative breakthrough curves for direct air capture of CO_2 (blue) and H_2O (black) by **K^{Me}Pyr** under a continuous flow of ambient air (flow rate = 0.2 L/min), monitored using a CO_2:humidity meter (LI-850, LI-COR). The breakthrough curves are shown over the complete monitoring period (left) and magnified over the initial 20 min (right). (**b-e**) Effect of inlet air flow rate on the time-dependent CO_2 uptake profiles of **K^{Me}Pyr** under a continuous flow of ambient air (20-40% RH) at (**b**) 0.2, (**c**) 1.0, (**d**) 2.0, and (**e**) 3.0 L/min, derived by integrating the breakthrough curves obtained using a CO_2:humidity meter (LI-850, LI-COR) to monitor the outlet CO_2 concentration. Each profile was fitted to the pseudo-first-order (PFO) kinetic model to derive the CO_2 adsorption half-time at the corresponding flow rate.

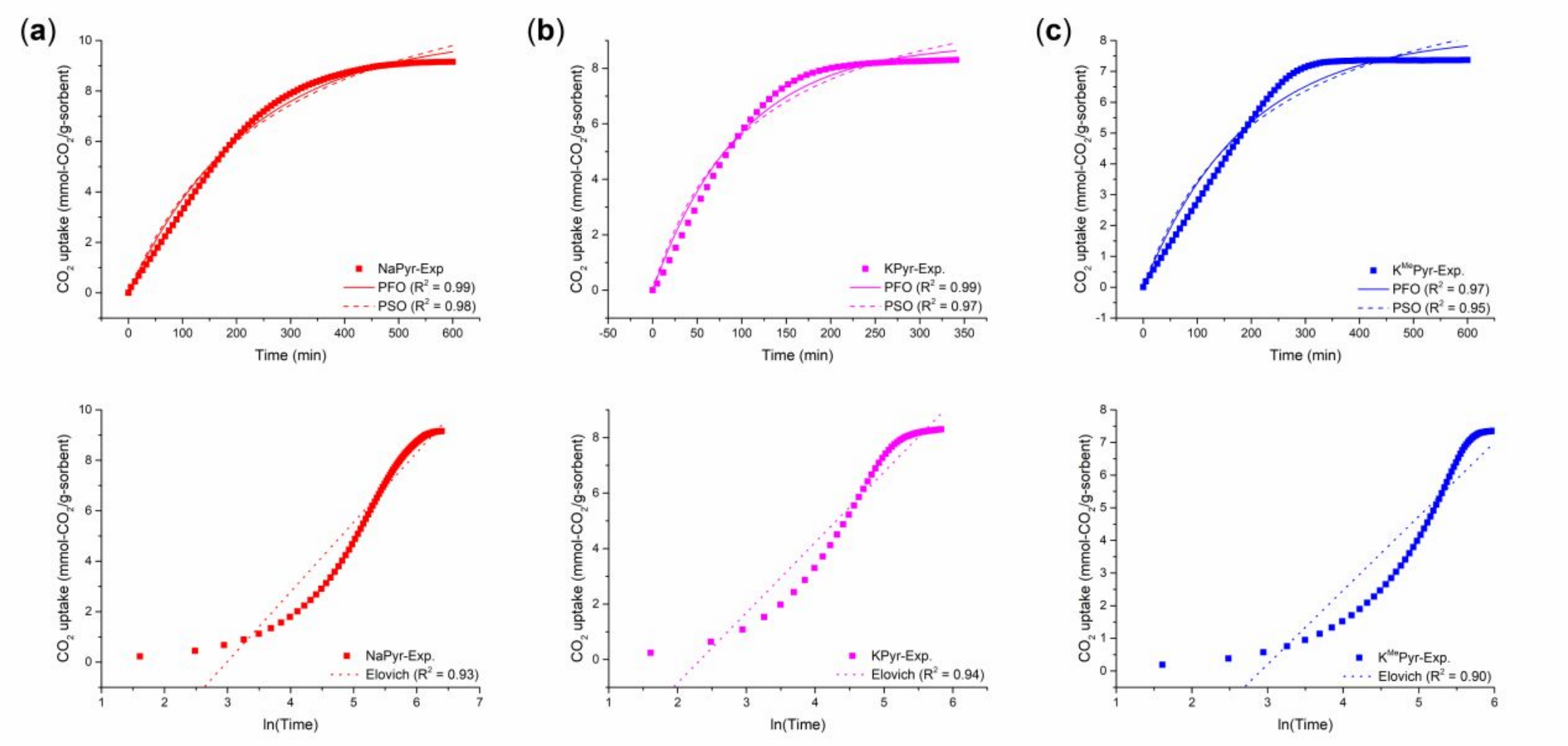

Figure S7. Time-dependent CO_2 uptake profiles of (**a**) **NaPyr** (red square), (**b**) **KPyr** (magenta square), and (**c**) **K^{Me}Pyr** (blue square) under a continuous flow of ambient air at 20%-40% RH (flow rate = 0.2 L/min), derived by integrating the breakthrough curves in Figures 7a-7c, in which the outlet CO_2 concentration was monitored using GC. These experimental CO_2 adsorption profiles were fitted to pseudo-first-order (PFO, solid lines) and pseudo-second-order (PSO, dash lines) kinetic models (top), and re-plotted against ln(Time) to evaluate the fit to the Elovich model (dotted lines, bottom).

(a)

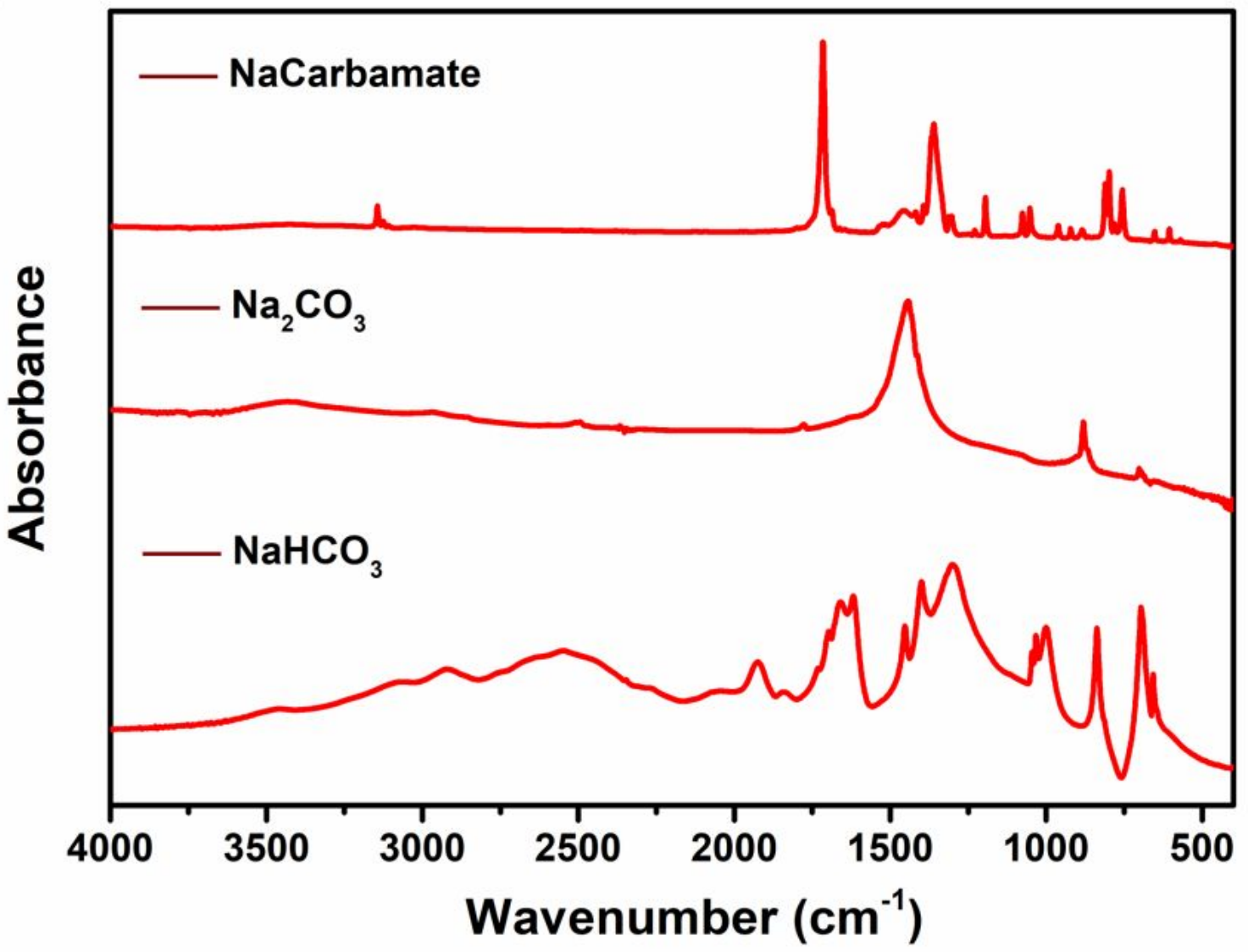
NaCarbamate
Na_2CO_3
$NaHCO_3$
Absorbance
4000
3500
3000
2500
2000
1500
1000
500
Wavenumber (cm^{-1})

(b)

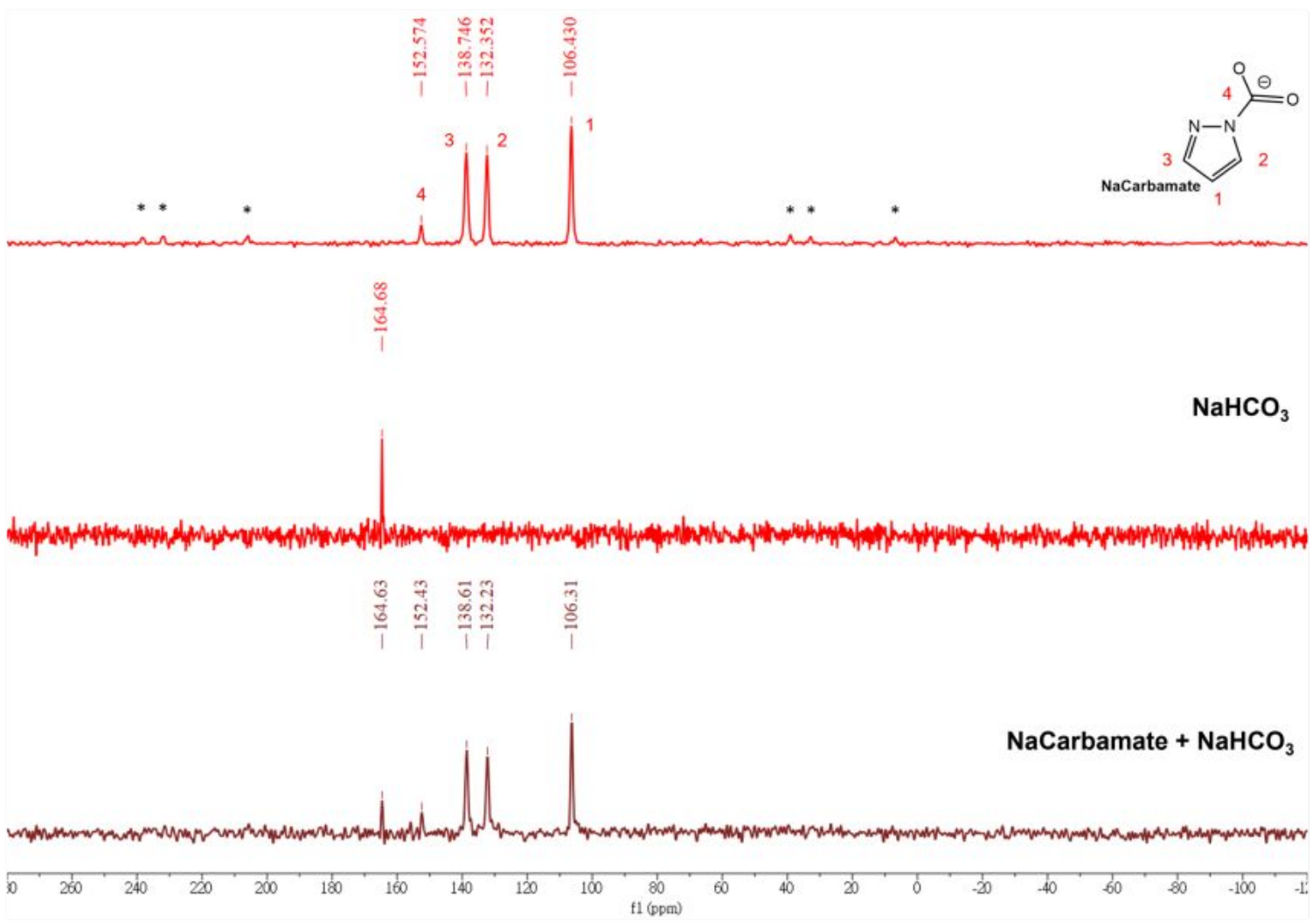
152.574
138.746
132.352
106.430
NaCarbamate
164.68
$NaHCO_3$
164.63
152.43
138.61
132.23
106.31
NaCarbamate + $NaHCO_3$
f1 (ppm)

(c)

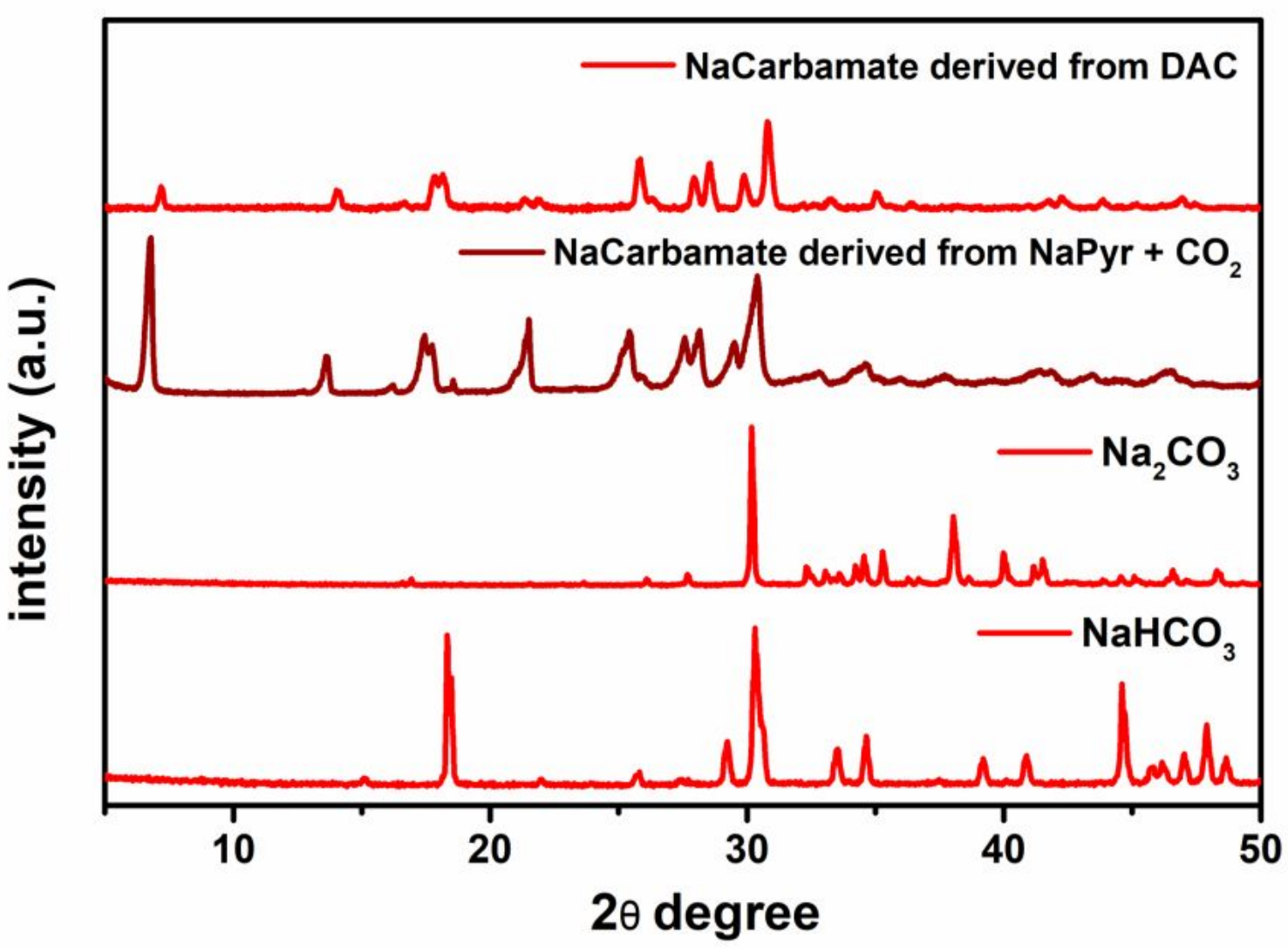
NaCarbamate derived from DAC
NaCarbamate derived from NaPyr + CO_2
Na_2CO_3
$NaHCO_3$
intensity (a.u.)
10
20
30
40
50
2θ degree

Figure S8. (**a**) Solid-state IR, (**b**) ^{13}C MAS NMR, and (**c**) PXRD spectra of Na_2CO_3, $NaHCO_3$, and CO_2-capture adduct derived from treatment of **NaPyr** with a continuous flow of ambient air (flow rate = 200 mL/min) for 6 h. PXRD spectrum of **NaCarbamate** derived from reaction of **NaPyr** and $CO_{2(g)}$ was depicted in maroon, whereas ^{13}C MAS NMR for a mixture of **NaCarbamate** and $NaHCO_3$ was depicted in maroon.

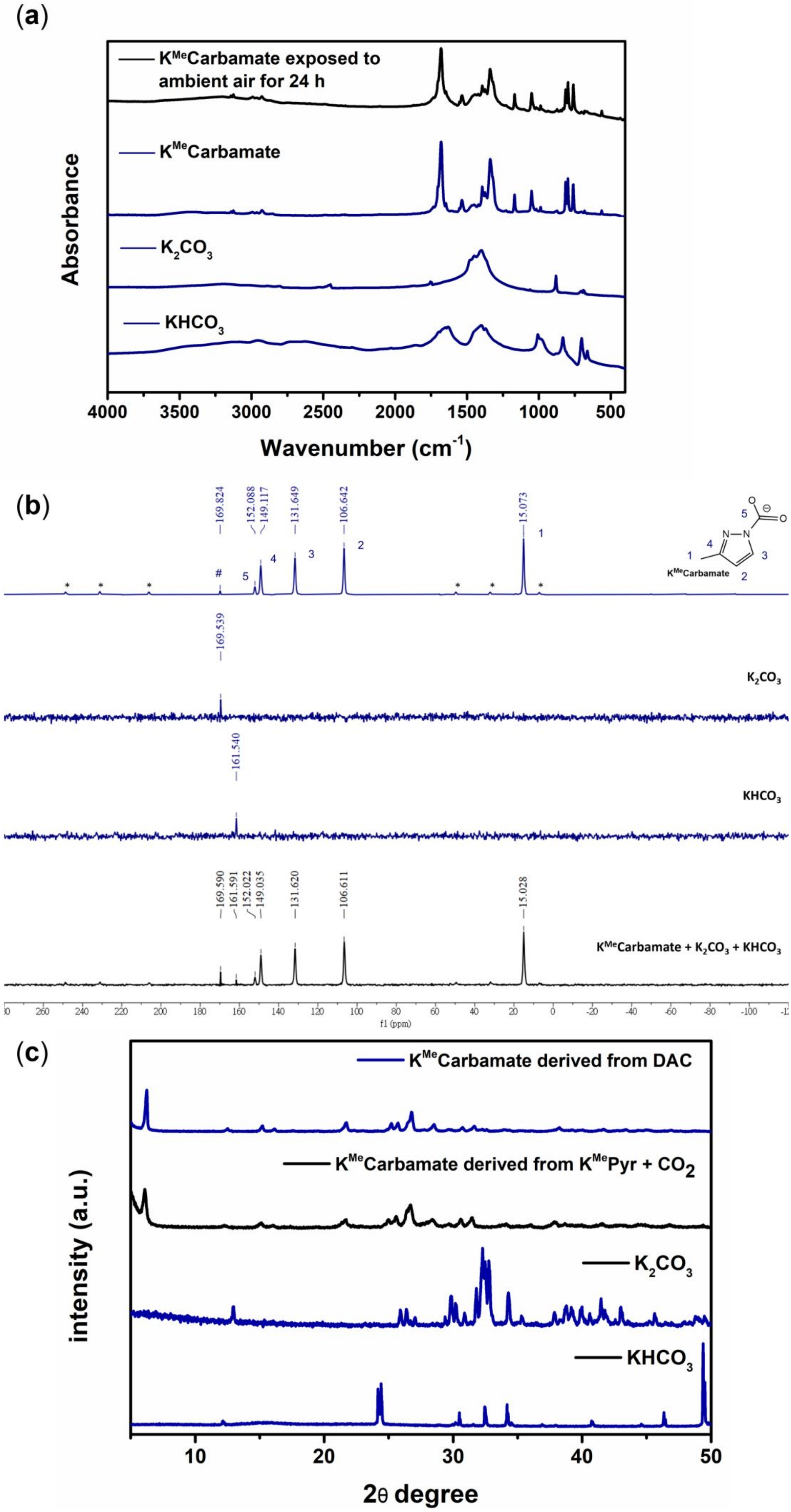

(a)
K^MeCarbamate exposed to ambient air for 24 h
K^MeCarbamate
K2CO3
KHCO3
Absorbance
4000
3500
3000
2500
2000
1500
1000
500
Wavenumber (cm-1)
(b)
169.824
152.088
149.117
131.649
106.642
15.073
1
2
3
4
5
#
K^MeCarbamate
169.539
K2CO3
161.540
KHCO3
169.590
161.591
152.022
149.035
131.620
106.611
15.028
K^MeCarbamate + K2CO3 + KHCO3
f1 (ppm)
(c)
K^MeCarbamate derived from DAC
K^MeCarbamate derived from K^MePyr + CO2
K2CO3
KHCO3
intensity (a.u.)
10
20
30
40
50
2θ degree

Figure S9. (**a**) Solid-state IR, (**b**) ^{13}C MAS NMR, and (**c**) PXRD spectra of K_2CO_3, $KHCO_3$, and CO_2-capture adduct derived from treatment of **K^{Me}Pyr** with a continuous flow of ambient air (flow rate = 200 mL/min) for 6 h. $^{\#}$: K_2CO_3. PXRD spectrum of **K^{Me}Carbamate** derived from reaction of **K^{Me}Pyr** and $CO_{2(g)}$ was depicted in black, whereas ^{13}C MAS NMR for a mixture of **K^{Me}Carbamate**, K_2CO_3 and $KHCO_3$ was depicted in black. Solid-state IR spectrum for treatment of **K^{Me}Pyr** with a continuous flow of ambient air (flow rate = 200 mL/min) for 24 h was depicted as black line in (**a**).

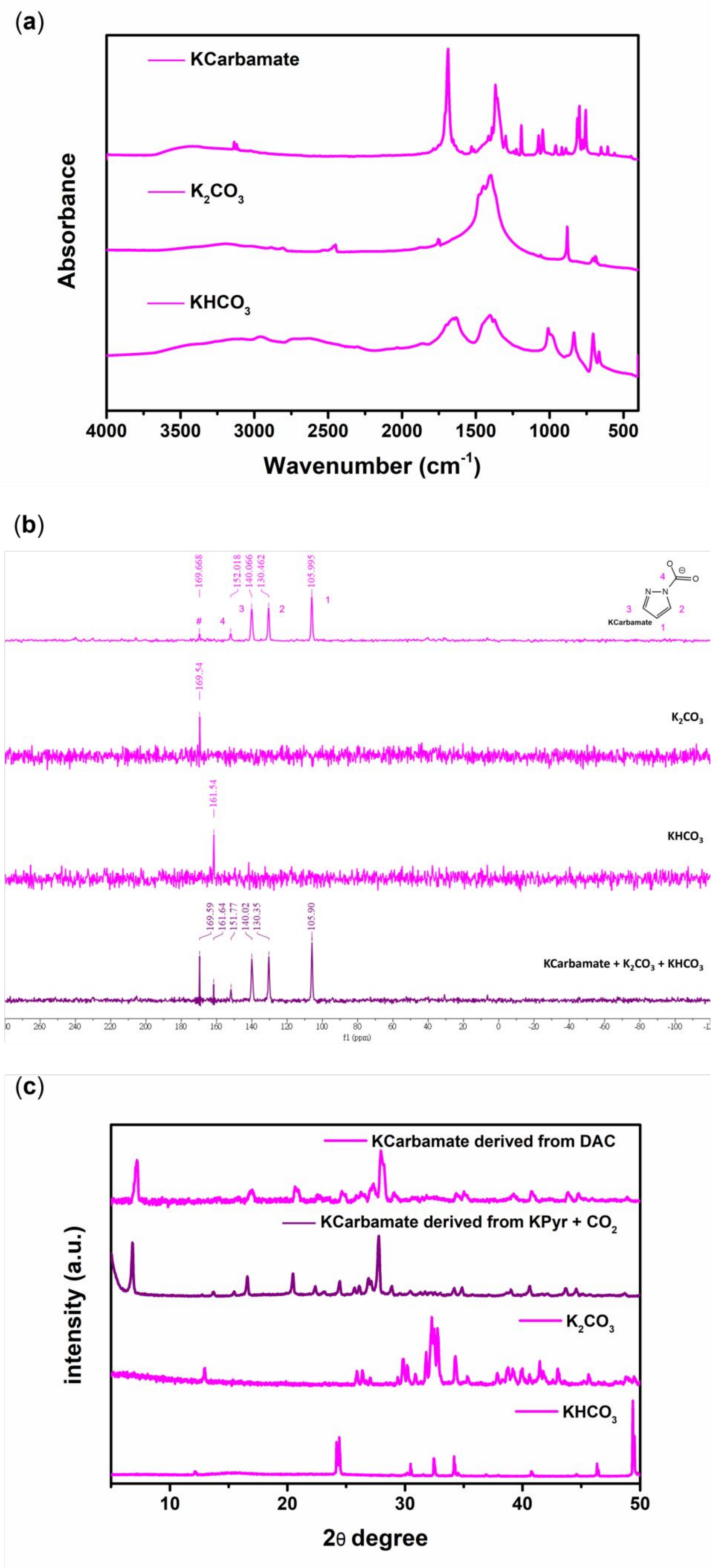

(a)
KCarbamate
K_2CO_3
$KHCO_3$
Absorbance
4000
3500
3000
2500
2000
1500
1000
500
Wavenumber (cm^{-1})
(b)
169.668
152.018
140.066
130.462
105.995
#
4
3
2
1
KCarbamate
169.54
K_2CO_3
161.54
$KHCO_3$
169.59
161.64
151.77
140.02
130.35
105.90
KCarbamate + K_2CO_3 + $KHCO_3$
f1 (ppm)
(c)
KCarbamate derived from DAC
KCarbamate derived from KPyr + CO_2
K_2CO_3
$KHCO_3$
intensity (a.u.)
10
20
30
40
50
2θ degree

Figure S10. (**a**) Solid-state IR, (**b**) ^{13}C MAS NMR, and (**c**) PXRD spectra of K_2CO_3, $KHCO_3$, and CO_2-capture adduct derived from treatment of **KPyr** with a continuous flow of ambient air (flow rate = 200 mL/min) for 6 h. $^{\#}$: K_2CO_3. PXRD spectrum of **KCarbamate** derived from reaction of **KPyr** and $CO_{2(g)}$ was depicted in purple, whereas ^{13}C MAS NMR for mixture of **KCarbamate**, K_2CO_3 and $KHCO_3$ was depicted in purple.

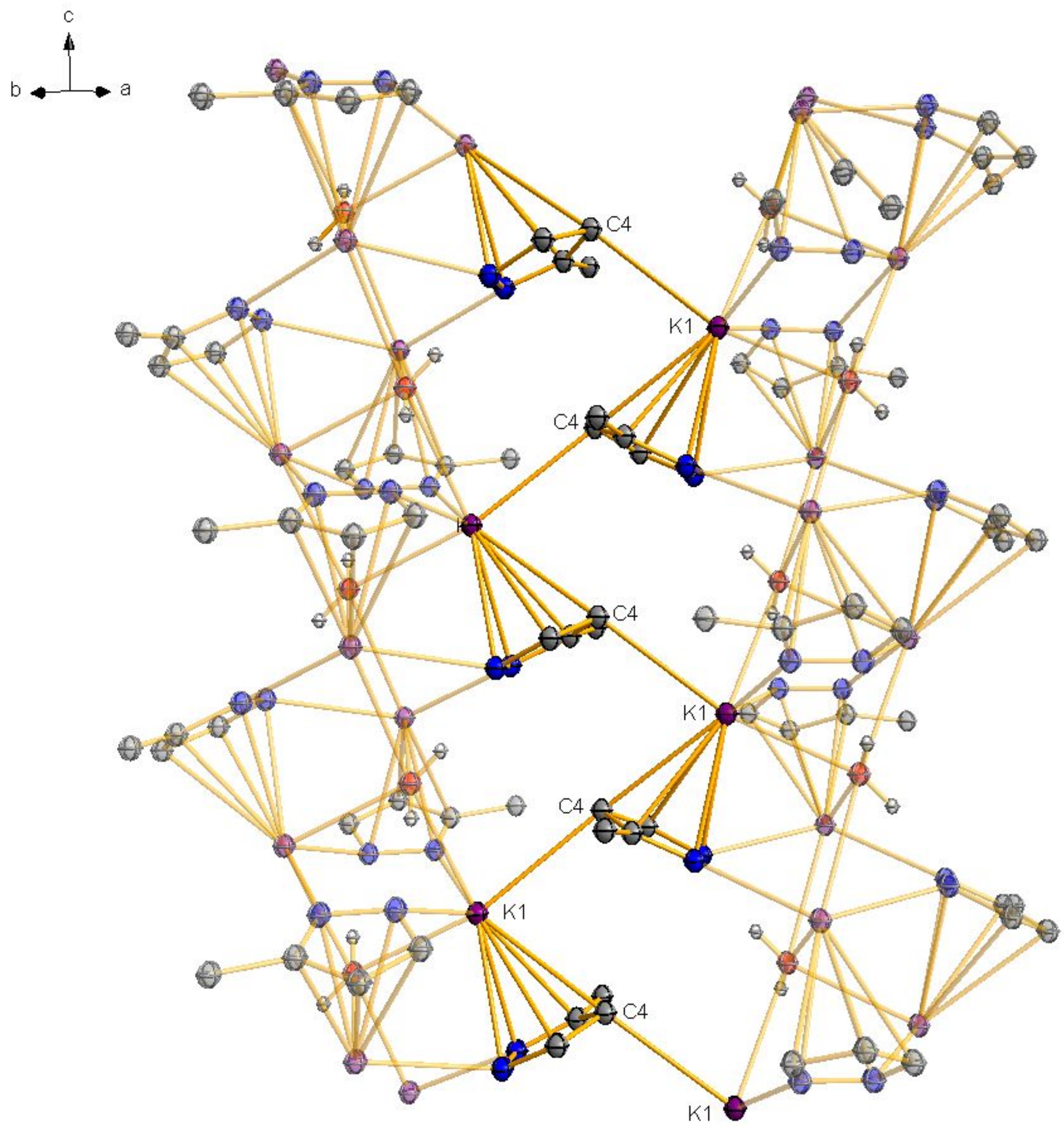

Figure S11. The η^1 interaction between two single chains in $\mathbf{K^{Me}Pyr{\bullet}H_2O}$.

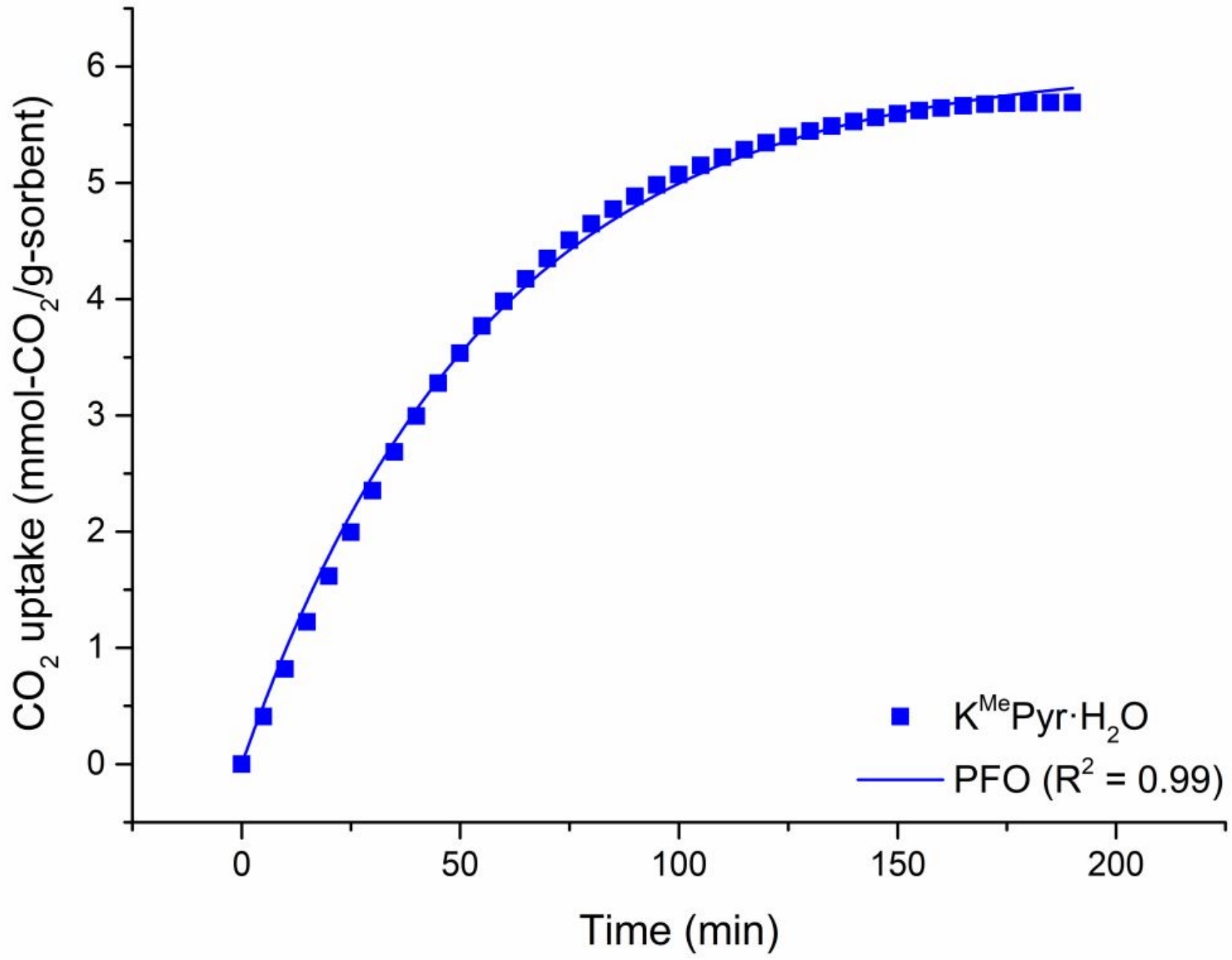

Figure S12. Time-dependent CO_2 uptake profiles of **K^{Me}Pyr•H_2O** upon exposure to a continuous flow of low-humidity air (flow rate = 0.2 L/min), derived by integrating the breakthrough curve (Figure 8c) obtained using a CO_2:humidity meter (LI-850, LI-COR) to monitor the outlet CO_2 concentration. The experimental CO_2 adsorption profile was fitted to the pseudo-first-order (PFO) kinetic model to derive the CO_2 adsorption half-time.

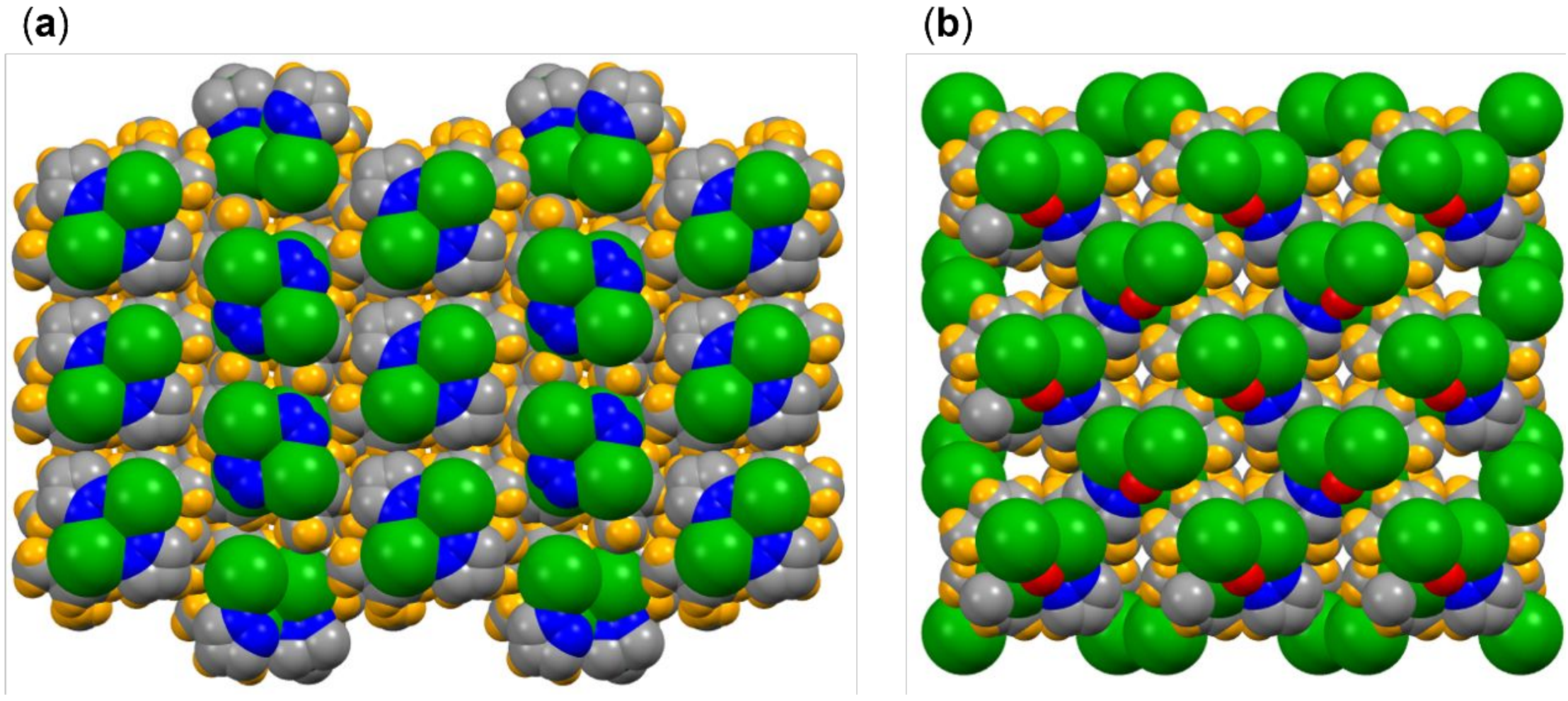

Figure S13. Space-filling models of (**a**) $\mathbf{K^{Me}Pyr}$ and (**b**) $\mathbf{K^{Me}Pyr{\bullet}H_2O}$ with all atoms in their appropriate van der Waals radii. Atom colors: C = gray, H = amber, N = blue, O = red, K = green.

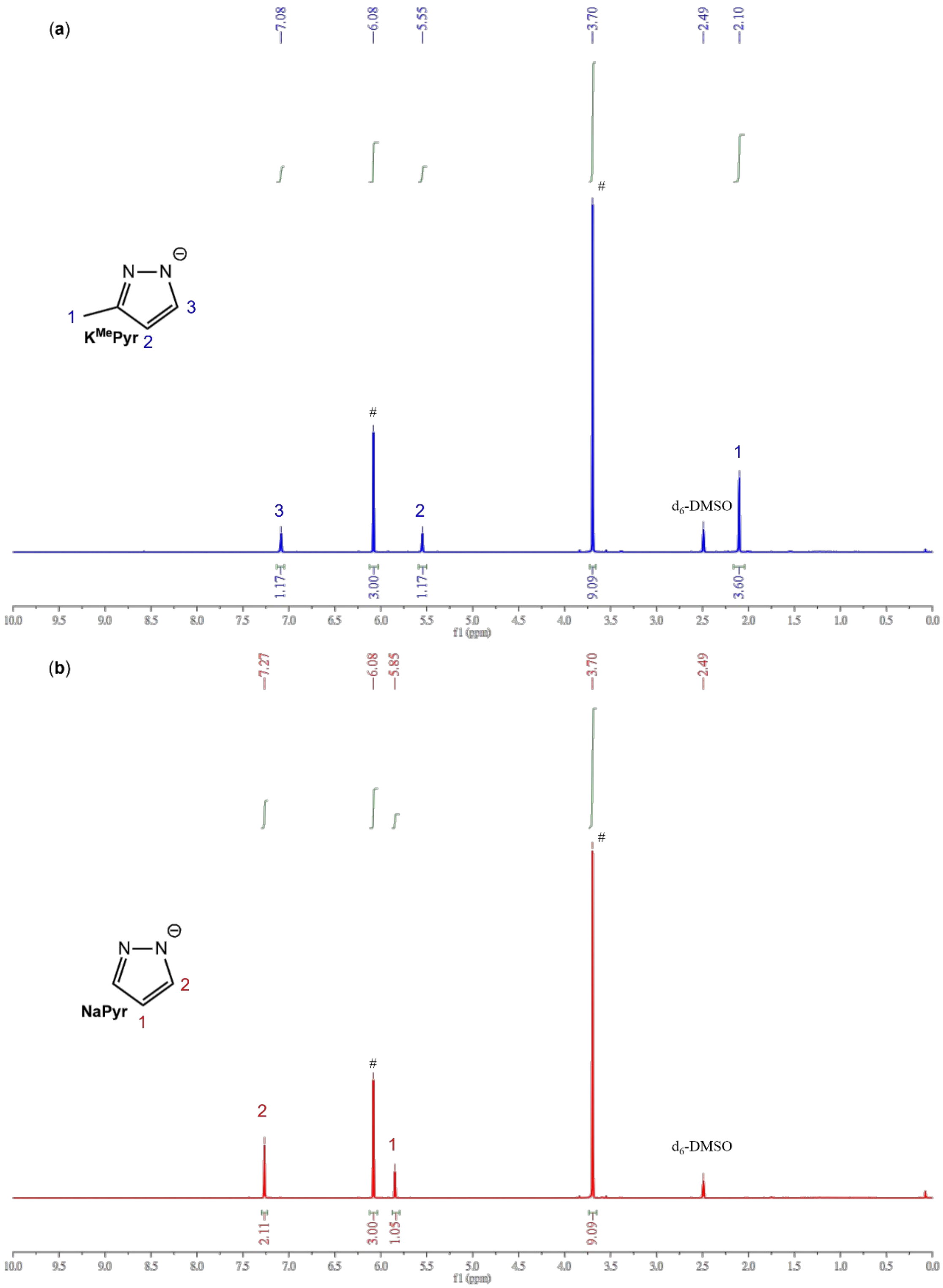

Figure S14. ^{1}H NMR spectra of regenerated (**a**) **K^{Me}Pyr** and (**b**) **NaPyr** in d$_6$-DMSO derived from incubation of **K^{Me}Carbamate** and **NaCarbamate** under vacuum at 200 °C for 1 h. Trimethoxybenzene was used as an internal standard (marked with #).

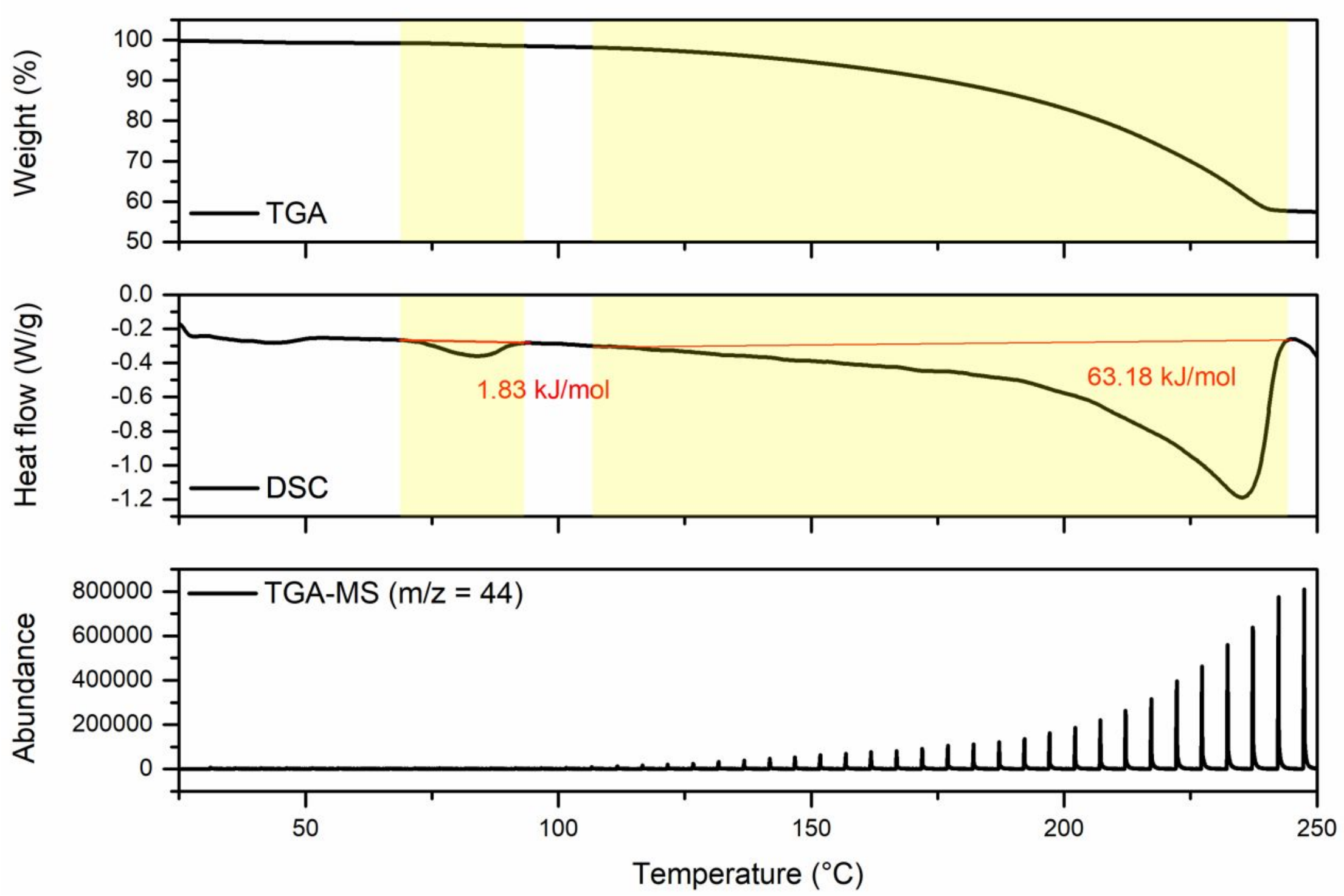

Figure S15. TGA (top), DSC (middle), and TGA-MS (for CO_2 at m/z = 44, bottom) analysis of **KCarbamate** derived from DAC using **KPyr**.

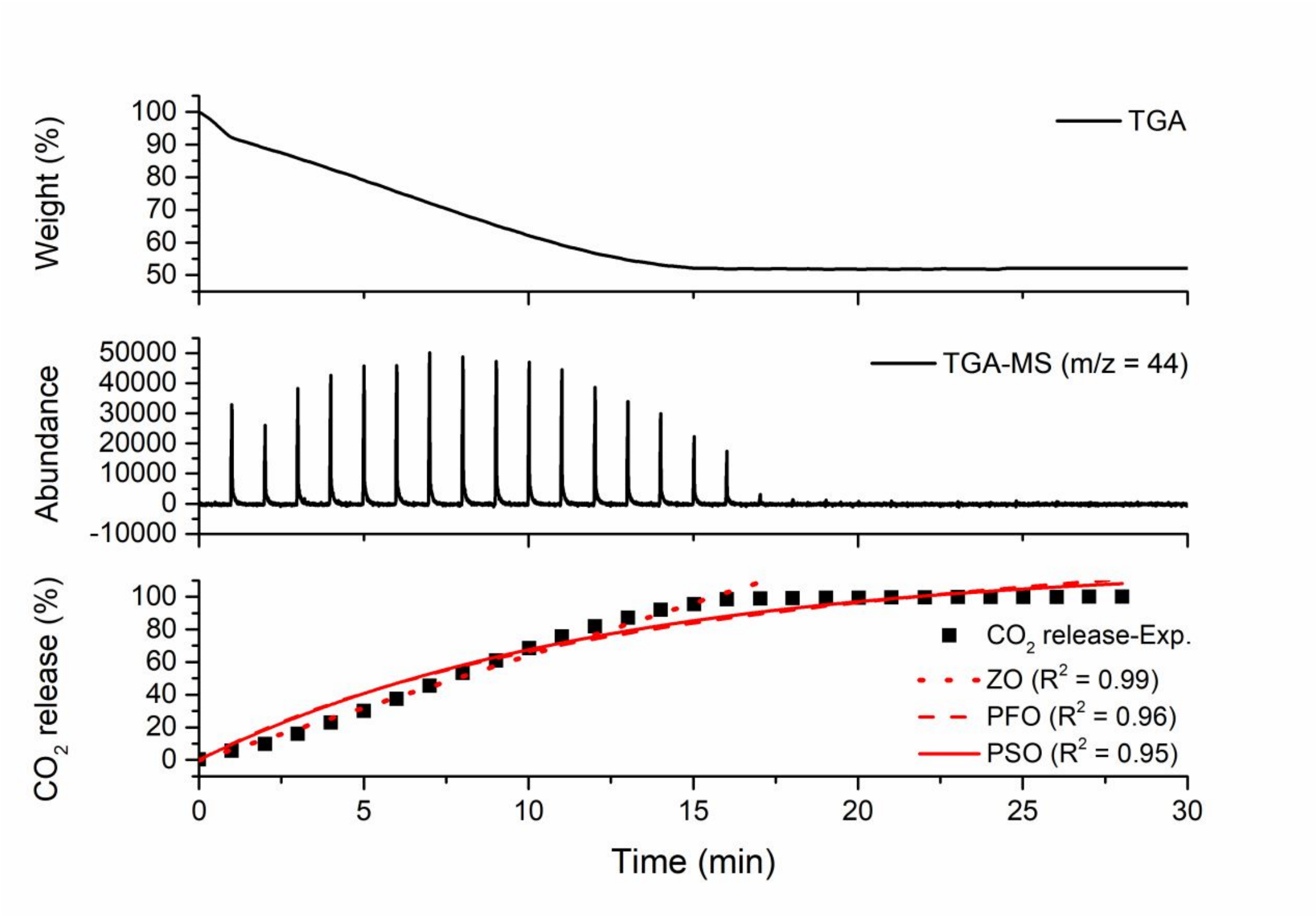

Figure S16. TGA curve (top), TGA-MS analysis (for CO_2 at m/z = 44, middle), and CO_2 release profile (bottom, black square) of **K^Me^Carbamate (**derived from DAC using **K^Me^Pyr**) under a continuous flow of $N_{2(g)}$ at 200 ^oC. The experimental CO_2 release profile was fitted using zero-order (ZO, dot lines), pseudo-first-order (PFO, solid lines), and pseudo-second-order (PSO, dash lines) models. These fittings were performed using Origin to determine the desorption half-time ($t_{1/2}$).

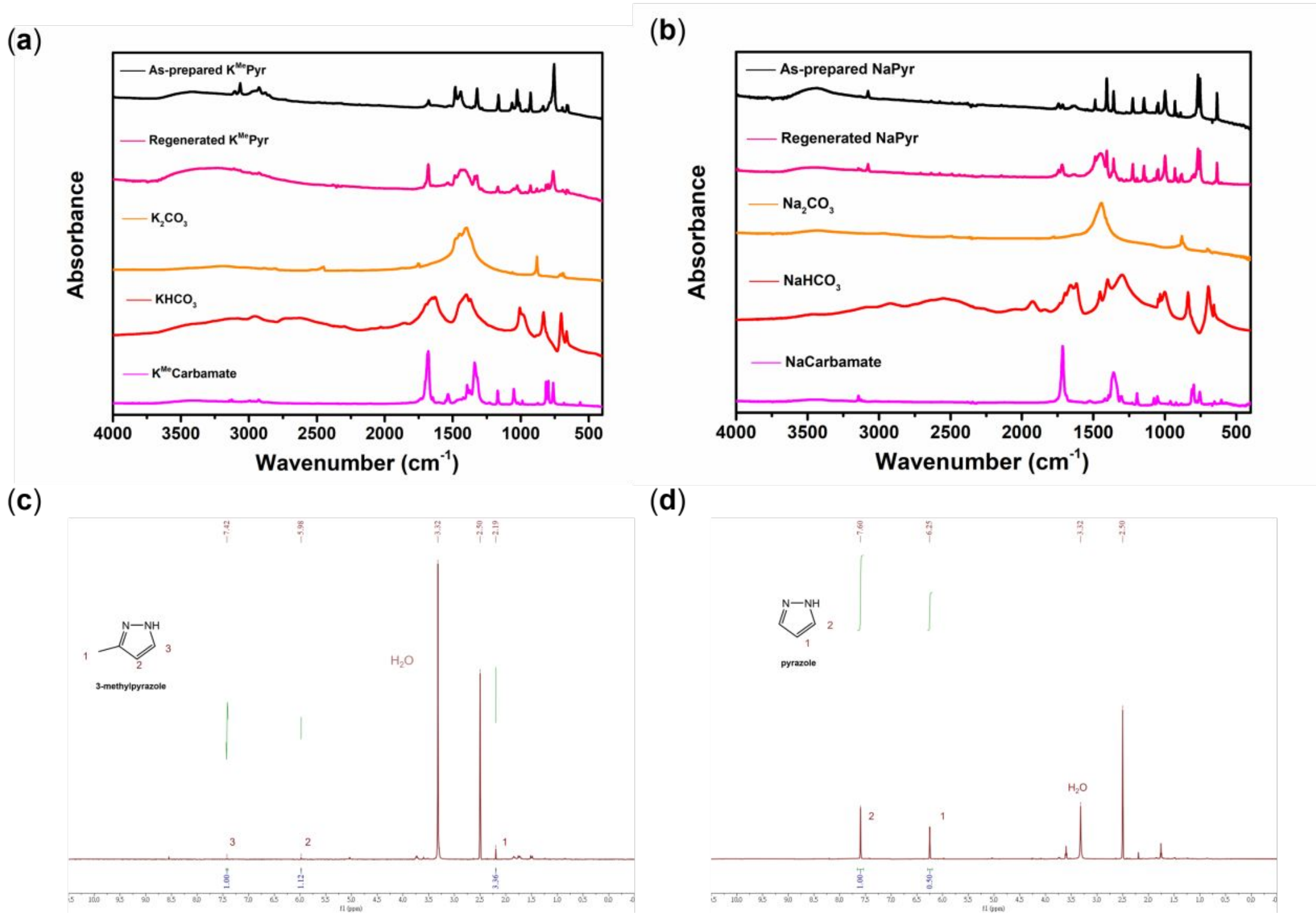

Figure S17. (**a**-**b**) Solid-state IR spectra for (**a**) **K^MeCarbamate** (magenta), $KHCO_3$ (red), K_2CO_3 (orange), as-prepared **K^MePyr** (black), and regenerated **K^MePyr** (pink) obtained from incubation of **K^MeCarbamate** under vacuum at 200 °C for 1 h; and (**b**) **NaCarbamate** (magenta), $NaHCO_3$ (red), Na_2CO_3 (orange), as-prepared **NaPyr** (black), and regenerated **NaPyr** (pink) obtained from incubation of **NaCarbamate** under vacuum at 200 °C for 1 h. (**c**-**d**) During the incubation of **K^MeCarbamate**/**NaCarbamate** under vacuum at 200 °C for 1 h, the evaporated (3-methyl)pyrazole and H_2O were collected using a cold trap incubated in $N_{2(l)}$. ^1H NMR spectra for (**c**) 3-methylpyrazole and H_2O in d_6-DMSO and (**d**) pyrazole and H_2O in d_6-DMSO collected during heat-induced regeneration of **K^MeCarbamate**/**NaCarbamate**.

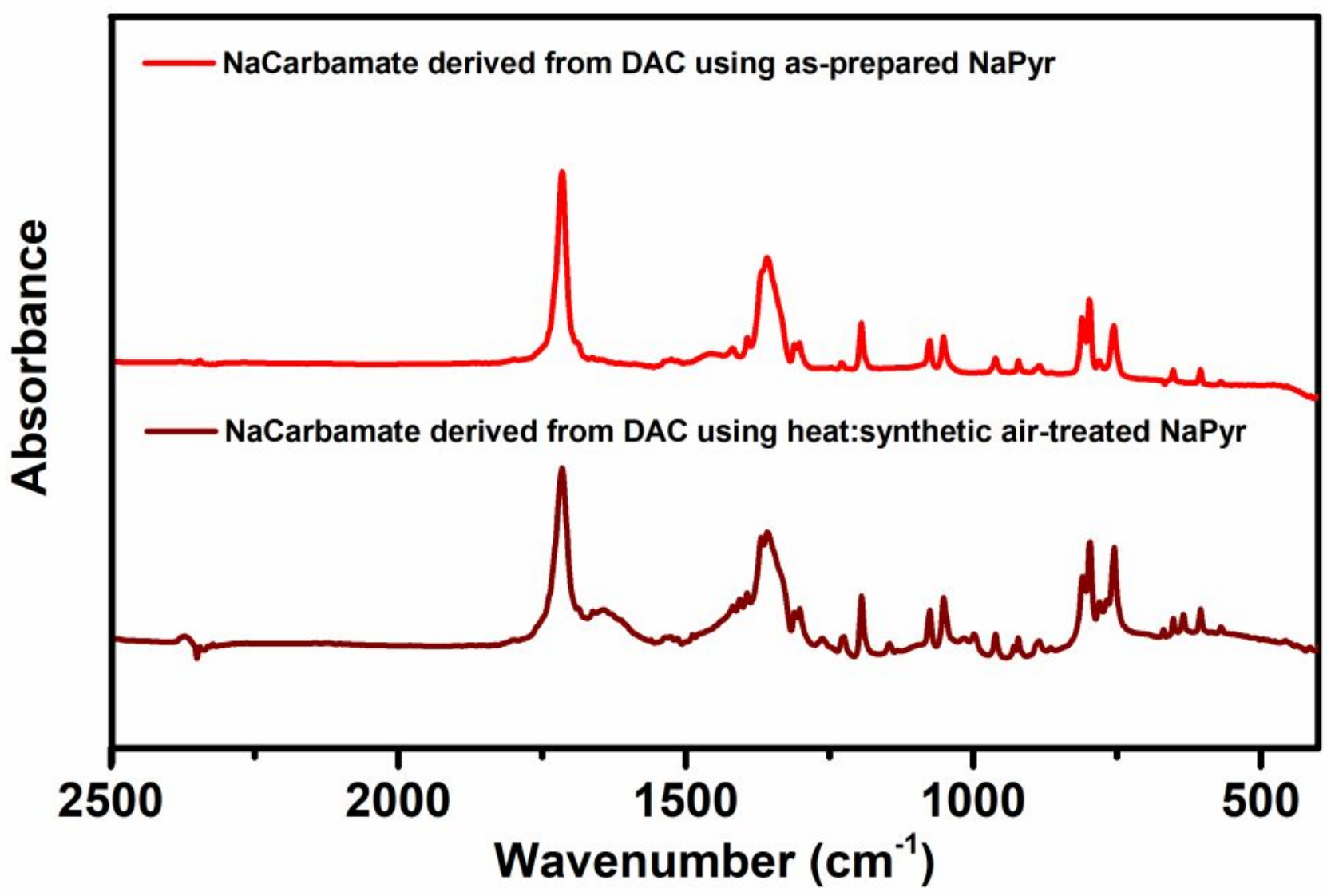

Figure S18. Solid-state IR spectra for the CO_2-capture adduct derived from treatment of as-prepared **NaPyr** (red) and heat:synthetic air-treated **NaPyr** (dark red) with a continuous flow of ambient air (flow rate = 200 mL/min).

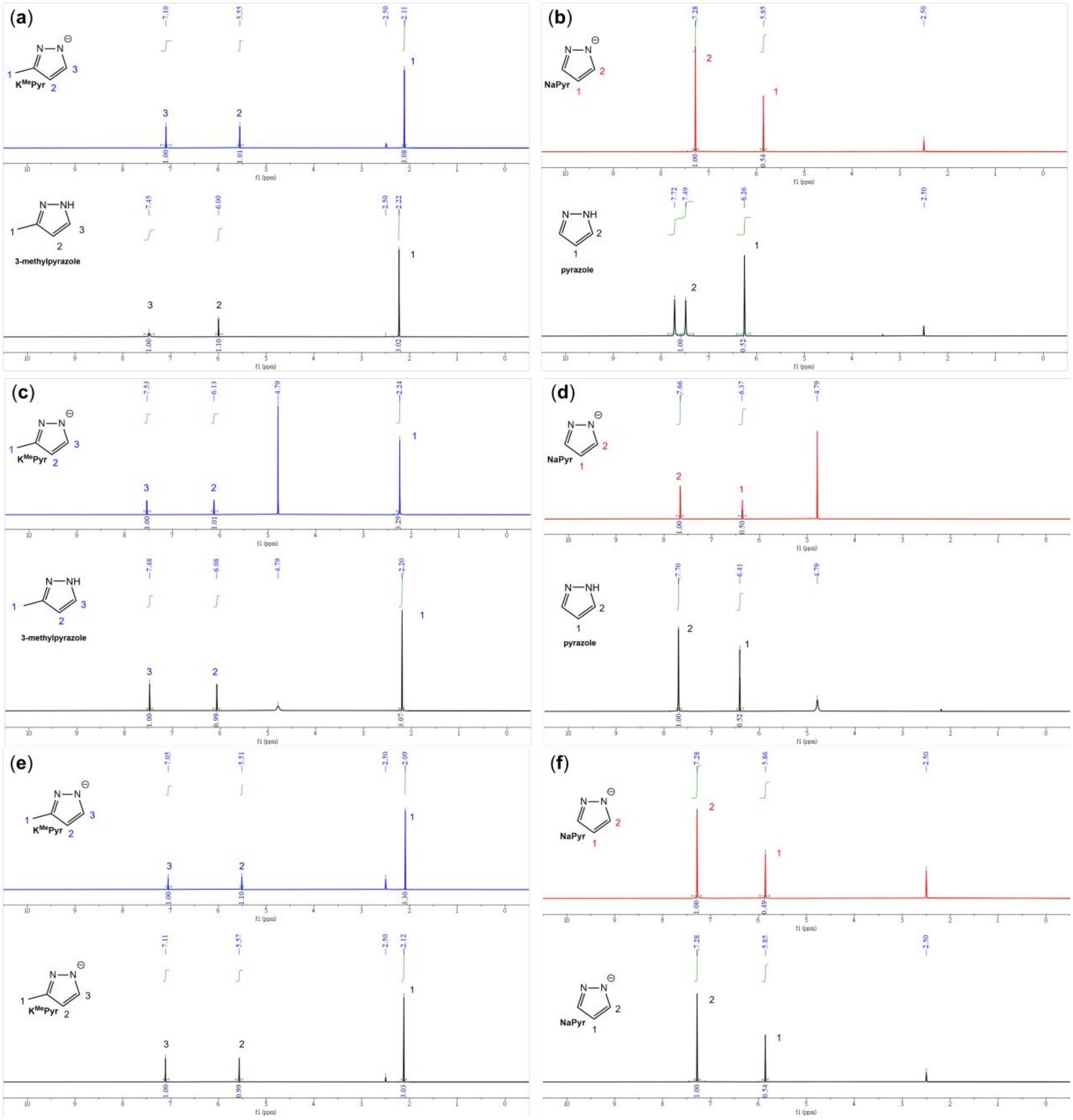

Figure S19. (**a-b**) ^{1}H NMR spectra of (**a**) **K^{Me}Pyr** (blue) and 3-methylpyrazole (black) in d$_6$-DMSO, and (**b**) **NaPyr** (red) and pyrazole (black) in d$_6$-DMSO. (**c-d**) ^{1}H NMR spectra of (**c**) **K^{Me}Pyr** (blue) and 3-methylpyrazole (black) in D_2O, and (**d**) **NaPyr** (red) and pyrazole (black) in D_2O. (**e-f**) ^{1}H NMR spectra of (**e**) recovered **K^{Me}Pyr** (blue) and (**f**) recovered **NaPyr** (red) in d$_6$-DMSO. The recovered **K^{Me}Pyr** and **NaPyr** were obtained after removal of D_2O from the aqueous solution of **K^{Me}Pyr** and **NaPyr**, respectively. ^{1}H NMR spectra for as-prepared **K^{Me}Pyr** and **NaPyr** in d$_6$-DMSO were depicted in black.

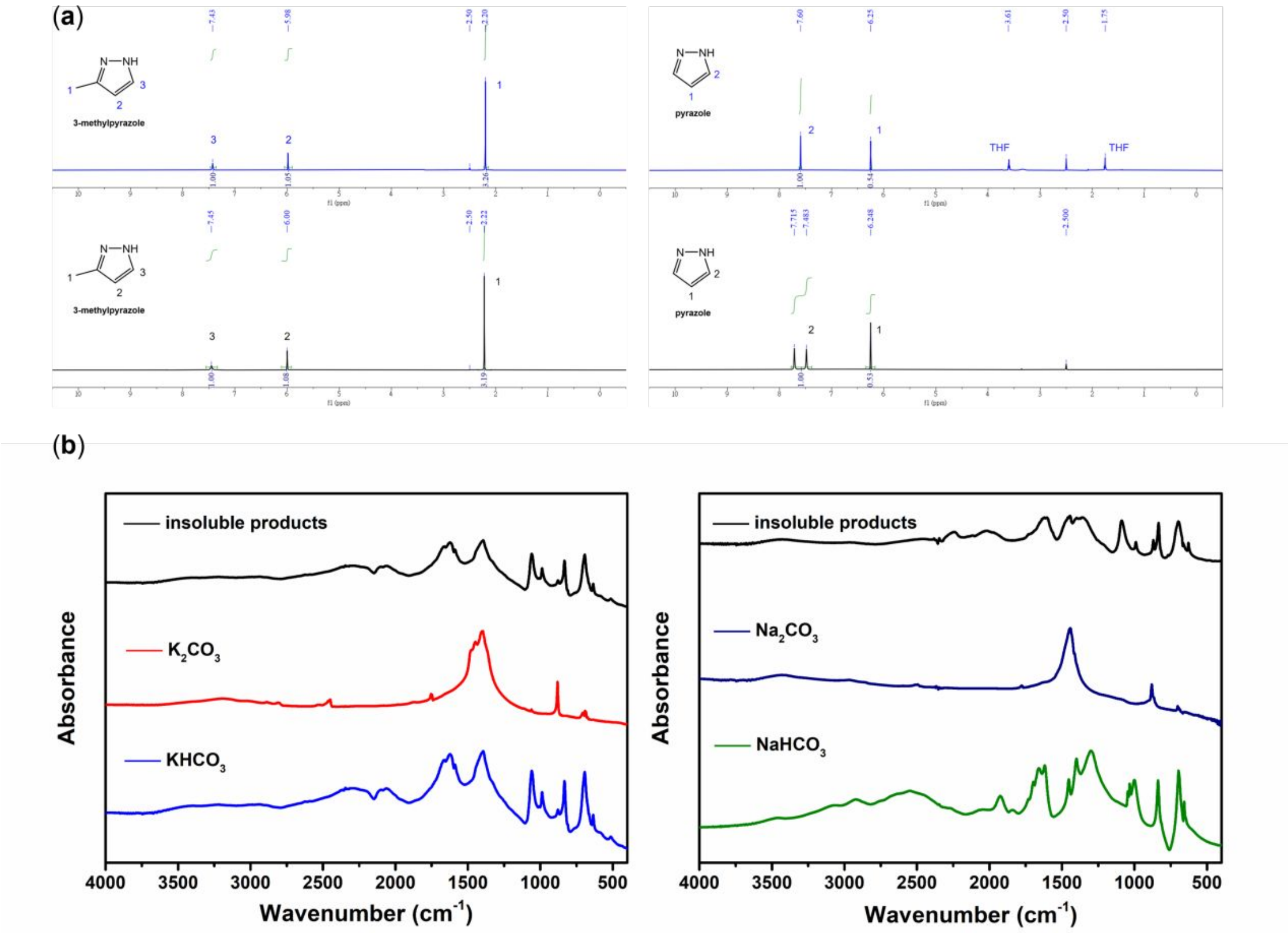

Figure S20. Characterizations of the products obtained from reaction of M^RPyr and one equiv. of CO_2 in H_2O. After removal of H_2O under vacuum, the obtained crude products were extracted with *n*-hexane when using **$K^{Me}Pyr$** and with THF when using **NaPyr**. (**a**) ^1H NMR spectra for the extracted products in d_6-DMSO (blue). ^1H NMR spectra for 3-methylpyrazole and pyrazole in d_6-DMSO were depicted in black. After the obtained crude products were washed with *n*-hexane and THF, the residual insoluble products were characterized using solid-state IR spectroscopy. (**b**) Solid-state IR spectra for the insoluble products (black), $KHCO_3$ (blue), K_2CO_3 (red), $NaHCO_3$ (olive), Na_2CO_3 (navy).

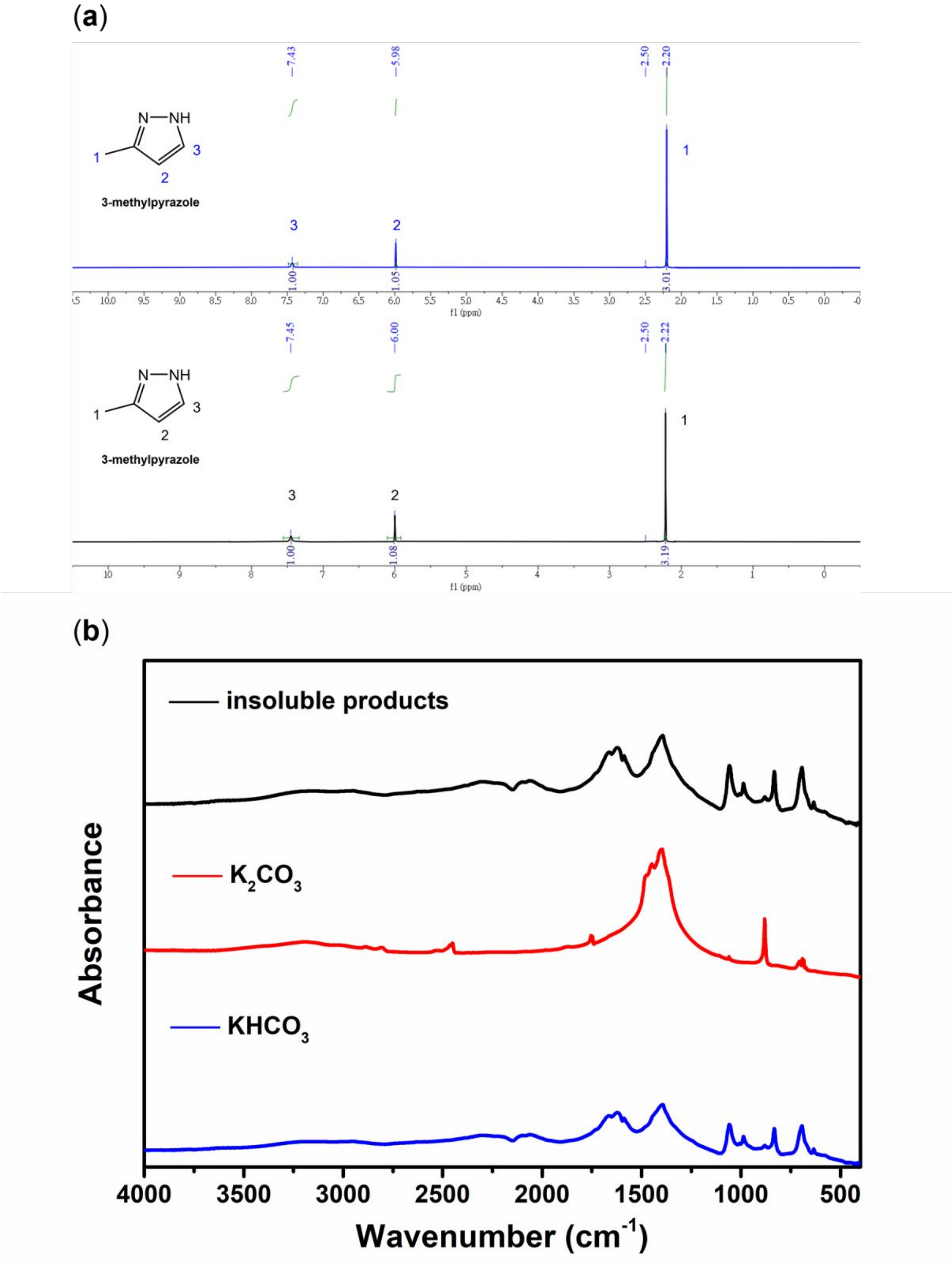

Figure S21. Characterizations of the products obtained from incubation of K^{Me}Carbamate in H_2O. After removal of H_2O from the aqueous solution of **K^{Me}Carbamate** under vacuum, the obtained crude products were extracted with *n*-hexane. (**a**) ^{1}H NMR spectra for the extracted product (blue) and 3-methylpyrazole (black) in d_6-DMSO. After the obtained crude products were washed with *n*-hexane and THF, the residual insoluble products were characterized using solid-state IR spectroscopy. (**b**) Solid-state IR spectra for the insoluble products (black), $KHCO_3$ (blue), K_2CO_3 (red).

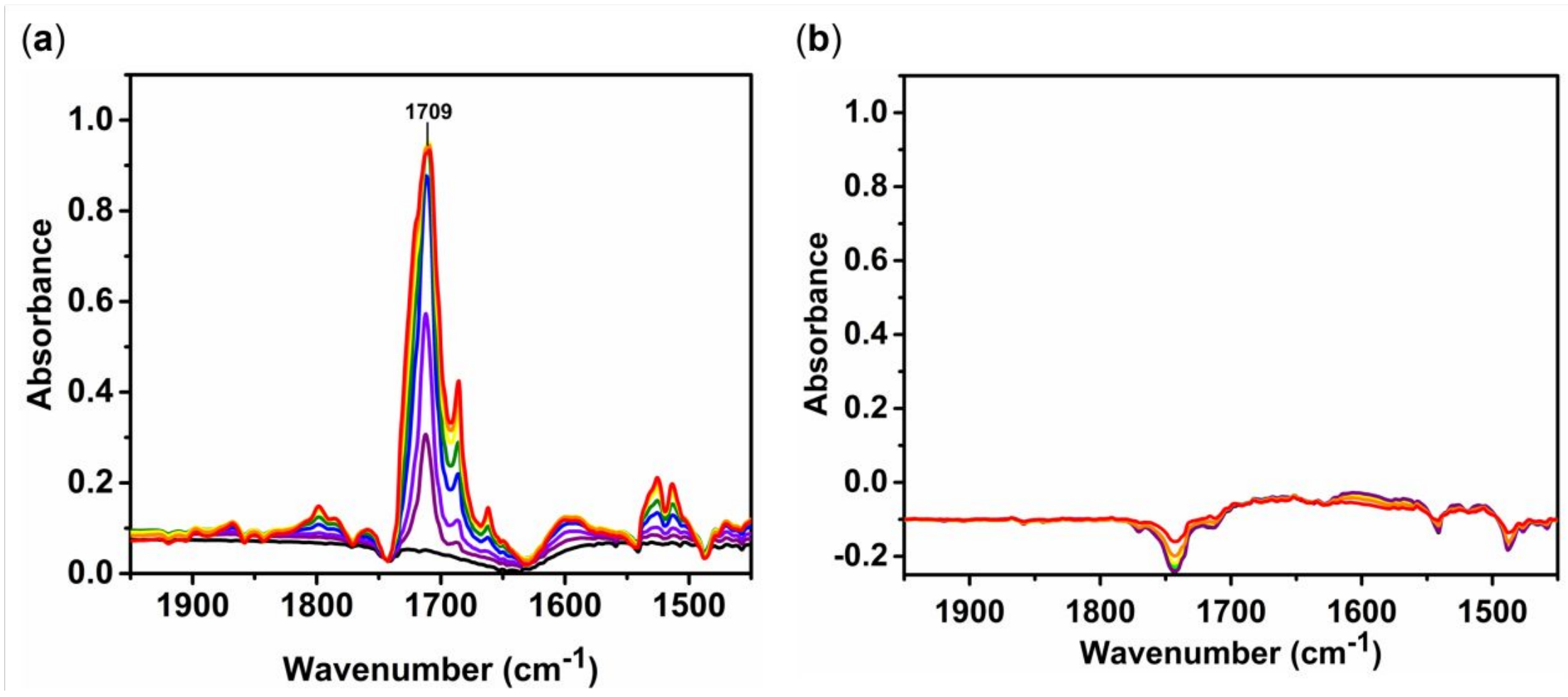

Figure S22. Solid-state IR spectra for **NaPyr** under a continuous flow of (**a**) ambient air and (**b**) low-humidity air, respectively, monitored by DRIFTS. These solid-state IR spectra were measured every minute.

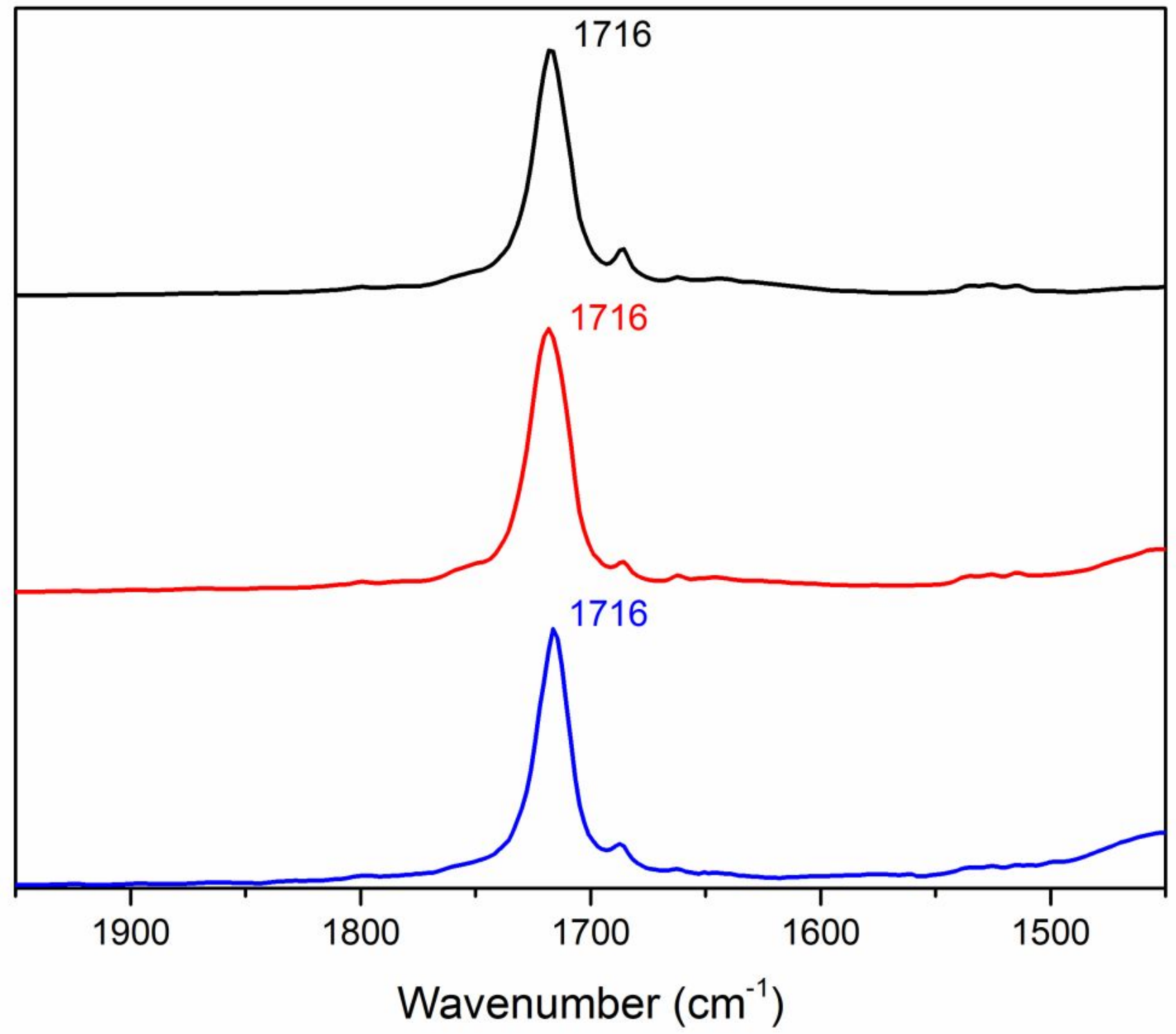

Figure S23. Solid-state IR spectra for the precipitated products derived from reaction of **NaPyr** with $CO_{2(g)}$ (black), ambient air (red), and low-humidity air (blue) in THF.

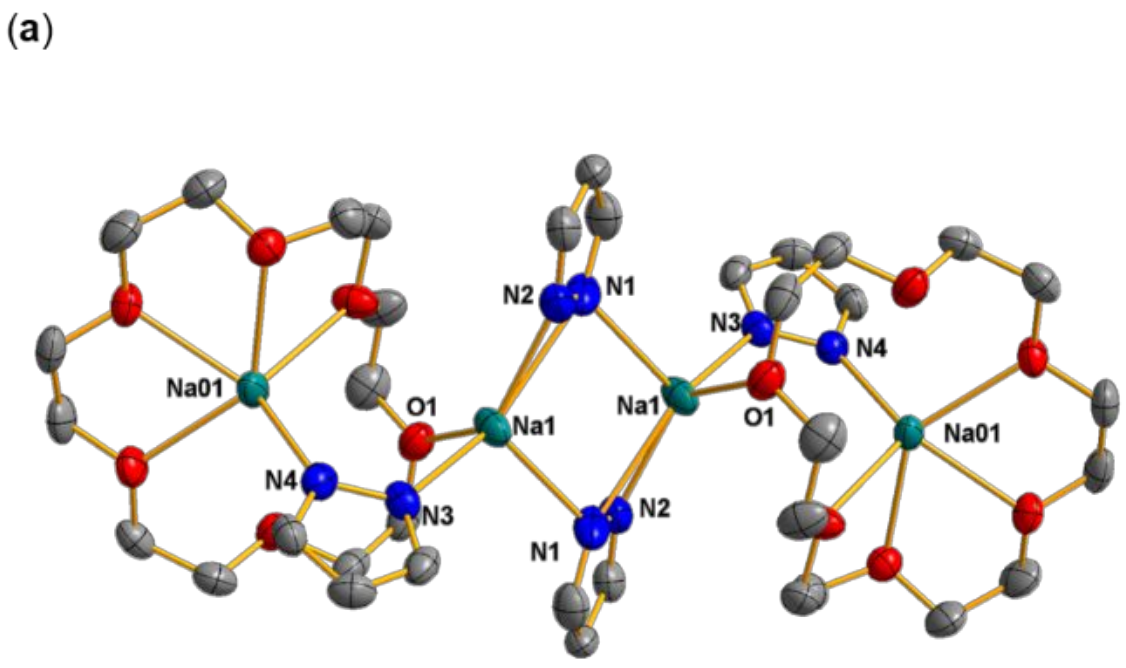

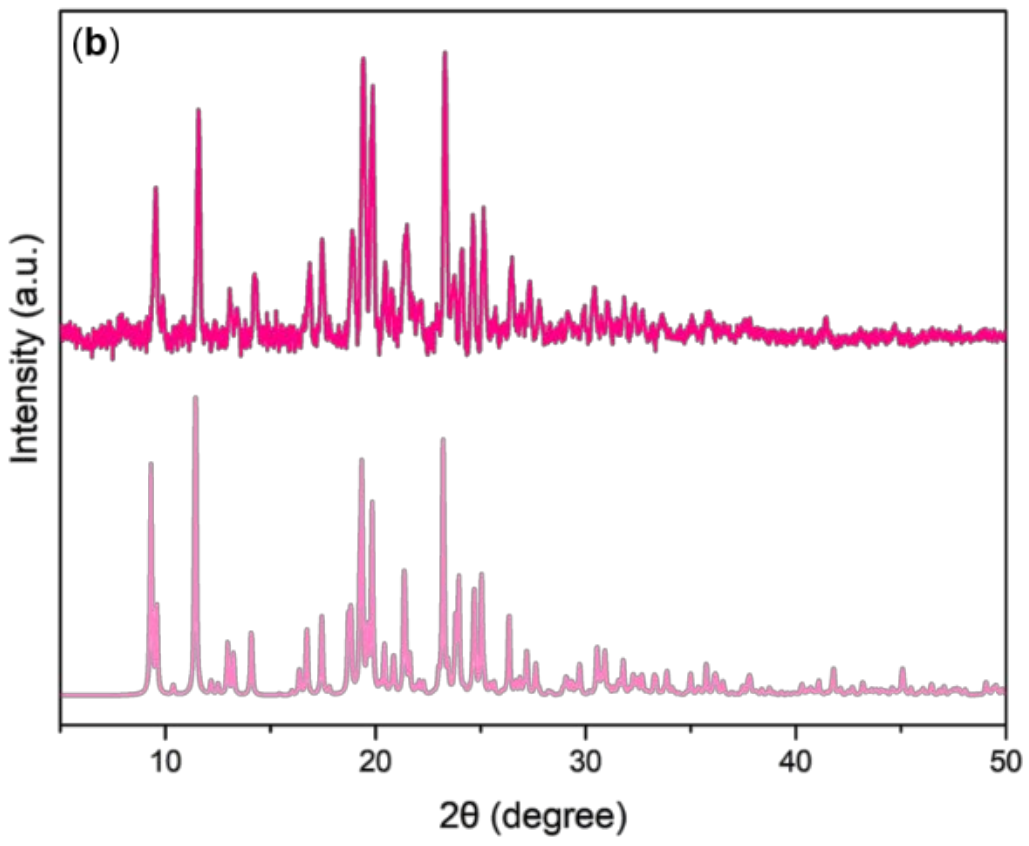

Figure S24. (**a**) ORTEP drawing and labeling scheme of molecular complex ([Na-18-crown-6-ether][Pyr])$_2$ (**NaCrownPyr**) with thermal ellipsoids drawn at 50% probability. (**b**) PXRD pattern of **NaCrownPyr** (pink). Calculated PXRD spectrum of single-crystal **NaCrownPyr** was depicted in light pink.

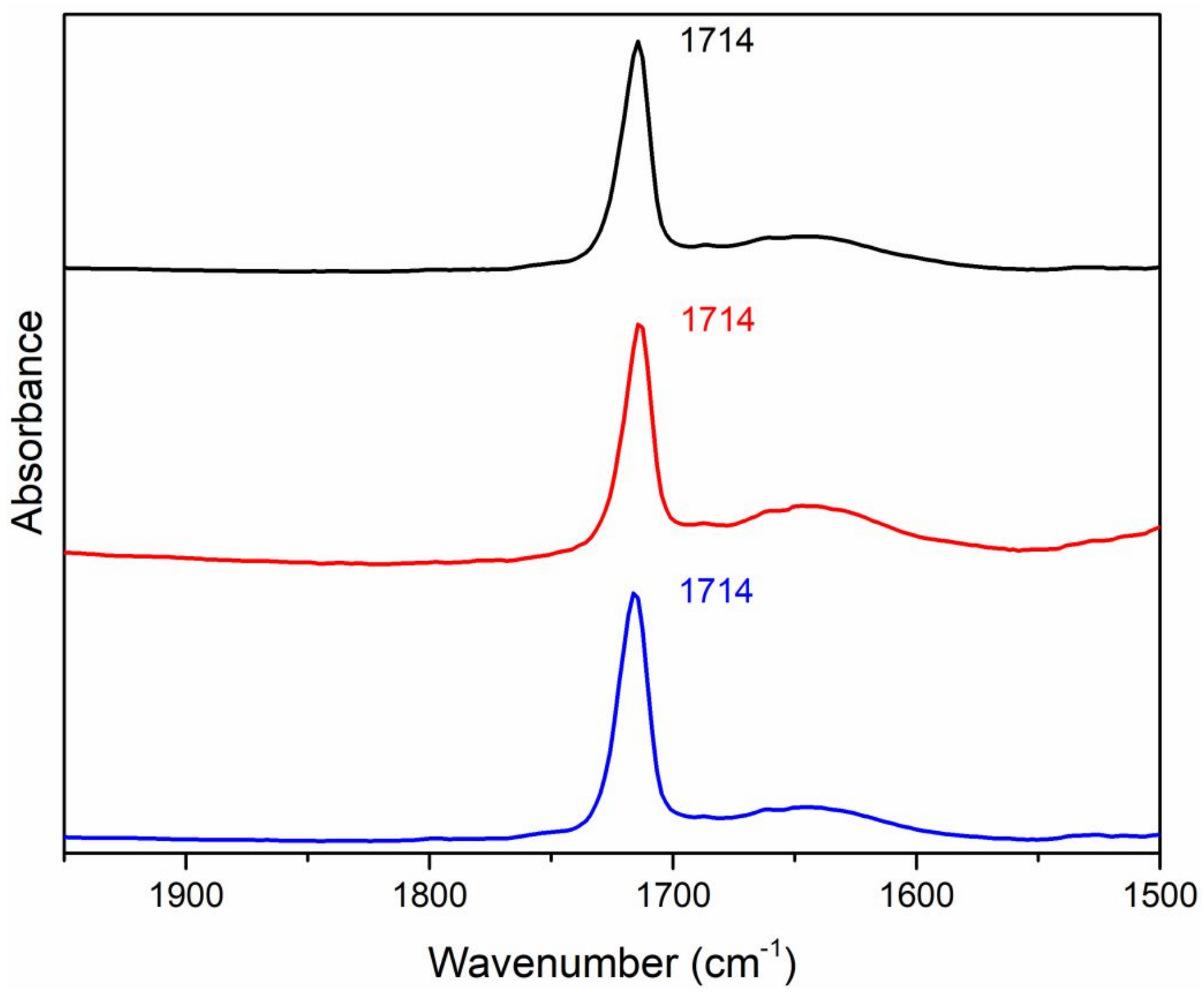

Figure S25. Solid-state IR spectra for reaction of solid-state **NaCrownPyr** with $CO_{2(g)}$ (black), ambient air (red), and low-humidity air (blue).

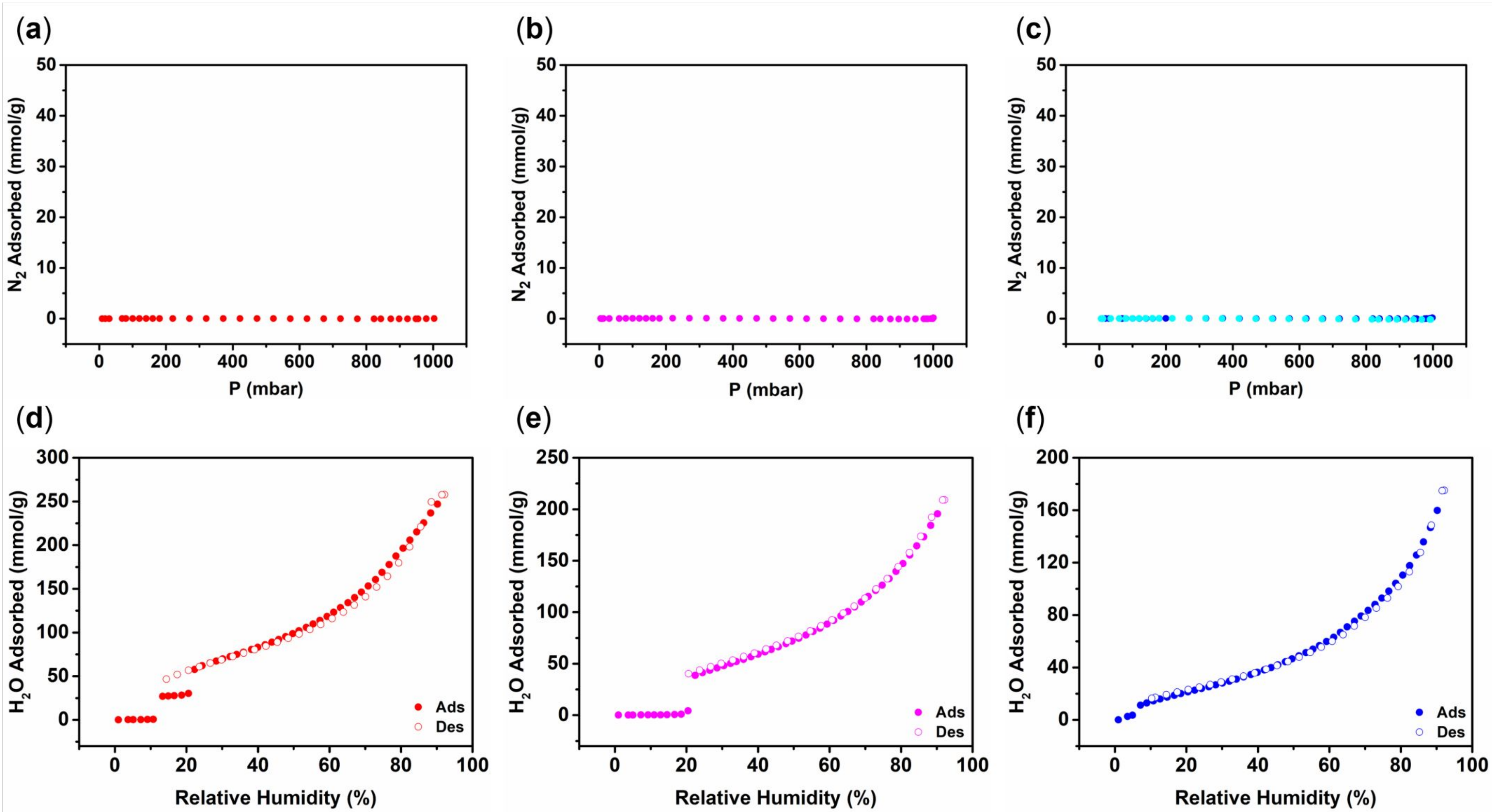

Figure S26. (**a**-**c**) N_2 adsorption isotherms of (**a**) **NaPyr** (red), (**b**) **KPyr** (magenta), and (**c**) **K^{Me}Pyr** (blue) and **K^{Me}Pyr•H_2O** (cyan) at 77 K. (**d**-**f**) H_2O adsorption (solid circles) and desorption (open circles) isotherms of (**d**) **NaPyr** (red), (**e**) **KPyr** (magenta), and (**f**) **K^{Me}Pyr** (blue) at 298 K.

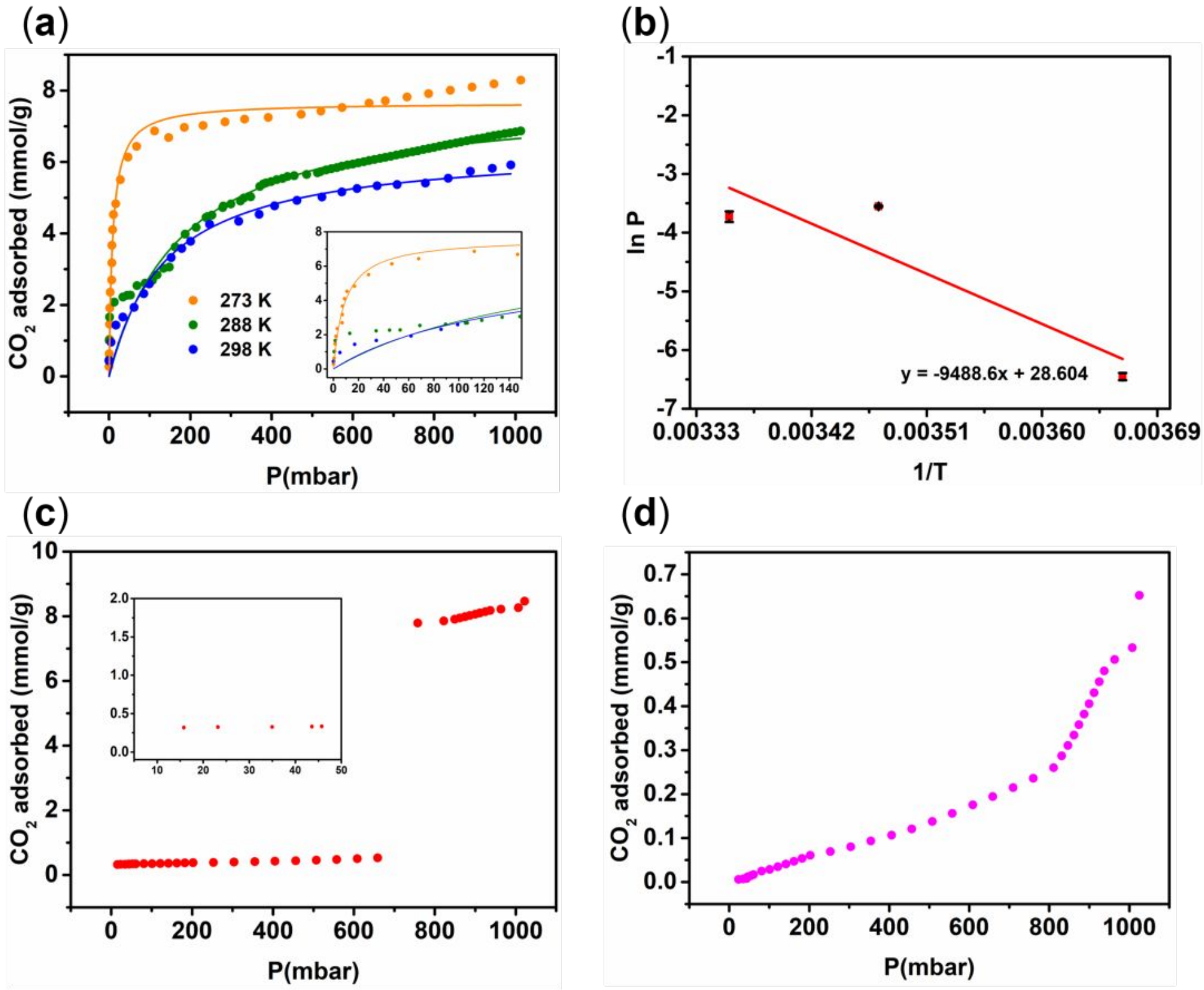

Figure S27. (**a**) CO_2 adsorption isotherms of **K^{Me}Pyr** at 298 K (blue), 288 K (olive), and 273 K (orange), which were fitted with Langmuir equation (solid lines). An enlarged view of the isotherms and Langmuir fits in the low-pressure region is provided in the inset. (**b**) Isosteric ln P against 1/T plot derived from CO_2 adsorption isotherms of **K^{Me}Pyr** at 298 K, 288 K, and 273 K. ΔH_{ads} and ΔS_{ads} were determined using the Clausius-Clapeyron equation. (**c-d**) CO_2 adsorption isotherms of (**c**) **NaPyr** and (**d**) **KPyr** at 298 K. An enlarged view of the isotherm for **NaPyr** in the low-pressure region is provided in the inset.

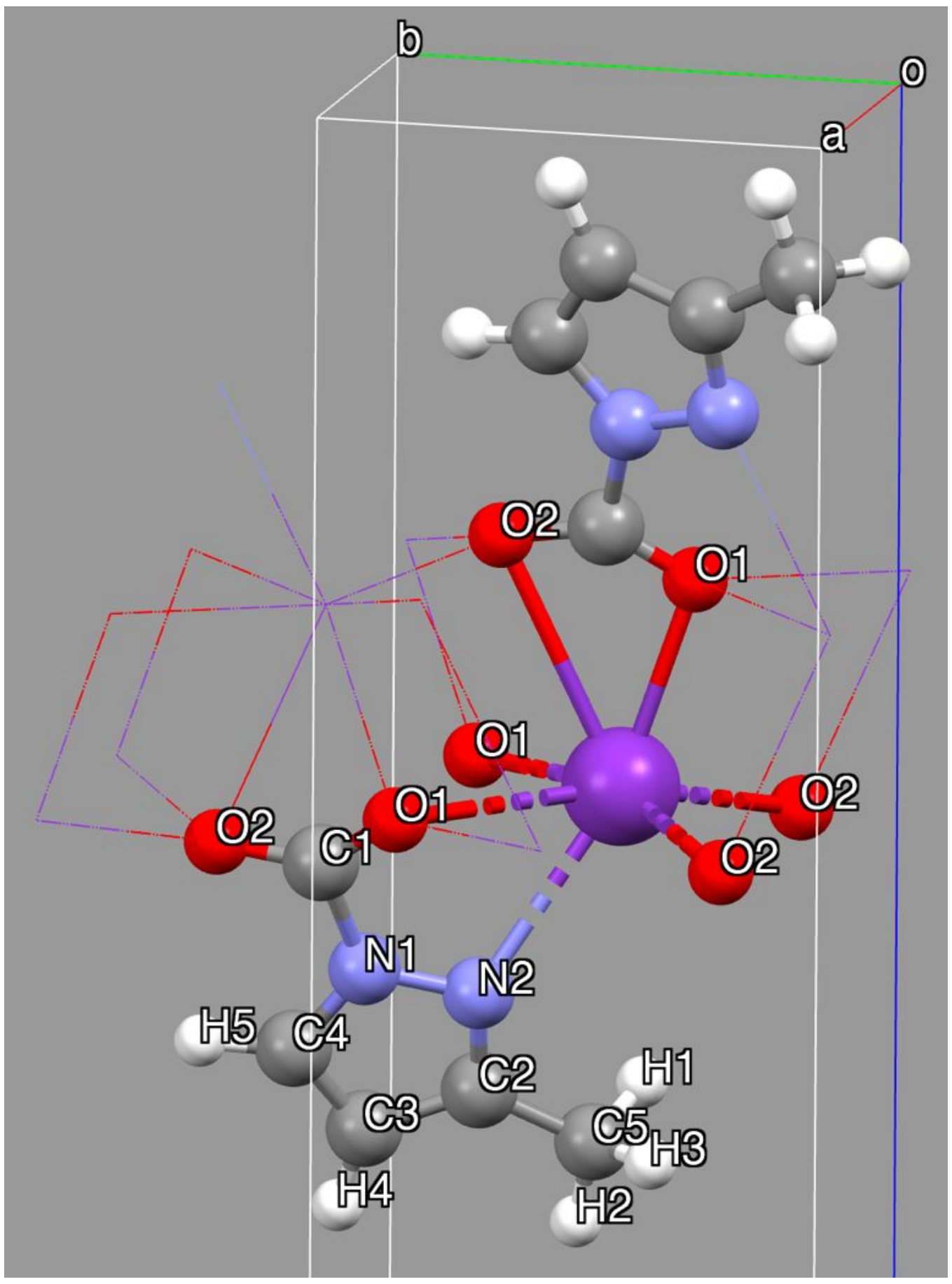

Figure S28. The initial [K][^{Me}PyrCO$_2$] (**K^{Me}Carbamate**) model was generated by the SA method for Rietveld structure refinement.

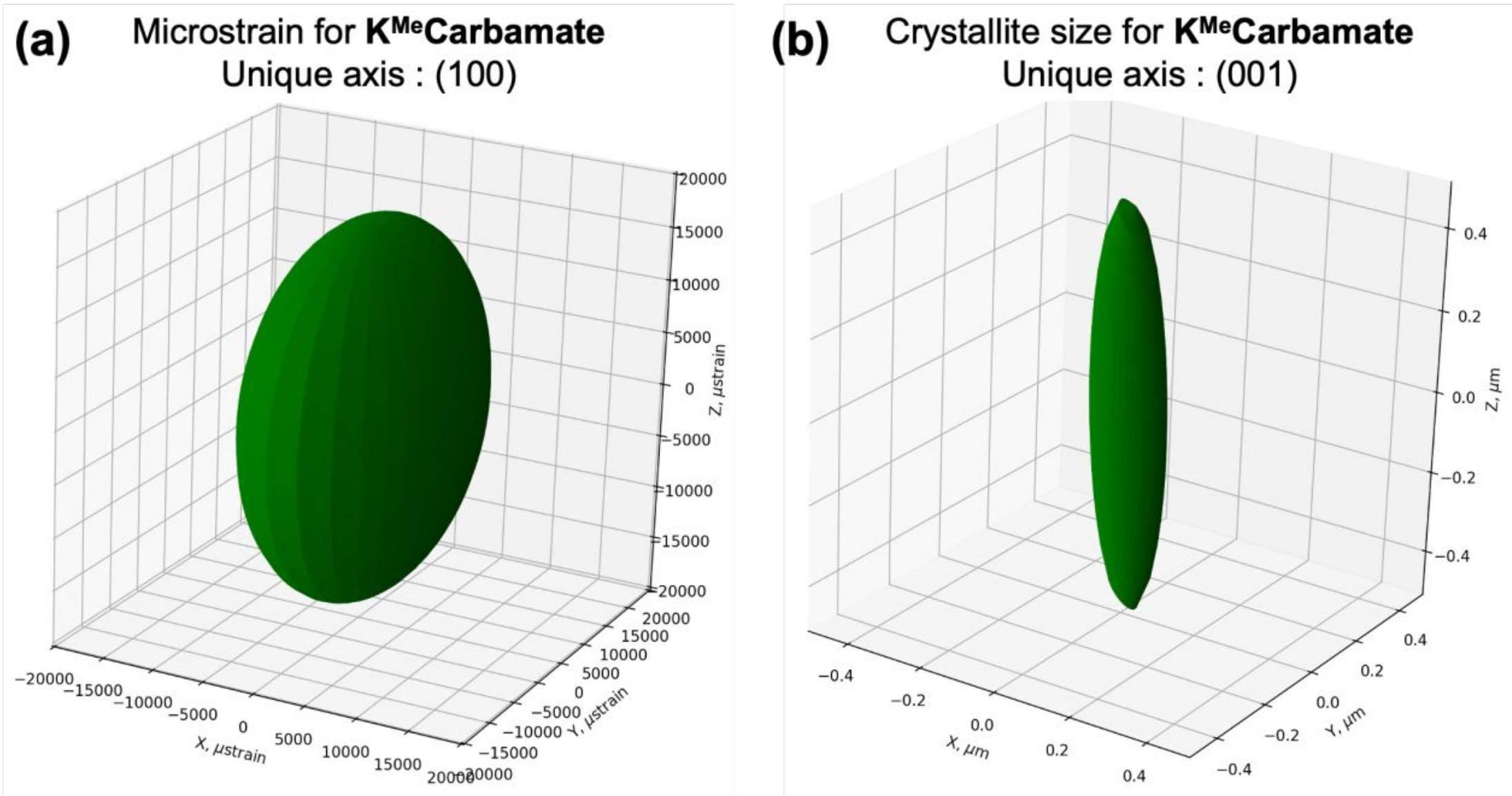

Figure S29. (**a**) Anisotropic microstrain [unique axis (100)] and (**b**) crystallite size [unique axis (001)] for **K^{Me}Carbamate**.

Table S1. Crystallographic data for **K^{Me}Carbamate**.

Formula	$C_5 H_5 K N_2 O_2$
Formula weight	164.21 g/mol
Radiation Source	TPS19A
λ (Å)	0.77489
Crystal system	Monoclinic
Space group	$P\ 2_1$/c
Temperature (K)	298 K
a (Å)	4.07466(18)
b (Å)	5.9243(5)
c (Å)	28.3952(28)
β (°)	92.755(6)
V (Å^3)	684.66(8)
Cell units, Z	4
μ strain (Δd/d)	Equatorial: 0.0095(3), Axial: 0.0177(1), unique axis (100)
Domain size (μm)	Equatorial: 0.205(19), Axial: 1.00 fixed, unique axis (001)
Zero shift (°)	-0.0037(8)
Rwp	8.5 %
R_F^2	11.7 %
2θ range (°)	2.4-64.0
Limiting indices	$0 \leq h \leq 5$, $0 \leq k \leq 8$, $-38 \leq l \leq 38$
Observe unique reflections	1835
Restraints	16
Constraints	1

Table S2. Atomic coordinates and displacement parameters of **K^{Me}Carbamate.**

Atom	x	y	z	Occupancy	Uiso
K1	0.2259(12)	0.5121(17)	0.29193(23)	1.0000	0.0402(23)
O1	0.215(4)	0.372(3)	0.2045(7)	1.0000	0.0275(30)
O2	0.304(4)	0.7357(31)	0.1887(6)	1.0000	0.0275(30)
C1	0.307(6)	0.526(6)	0.1828(8)	1.0000	0.0275(30)
N1	0.460(5)	0.471(5)	0.1353(8)	1.0000	0.0275(30)
N2	0.572(5)	0.259(6)	0.1246(8)	1.0000	0.0275(30)
C2	0.685(7)	0.279(5)	0.0811(10)	1.0000	0.0275(30)
C3	0.648(6)	0.501(7)	0.0633(10)	1.0000	0.0275(30)
C4	0.507(8)	0.619(4)	0.0986(14)	1.0000	0.0275(30)
C5	0.830(7)	0.077(6)	0.0591(10)	1.0000	0.0275(30)
H1	0.94(5)	-0.05(4)	0.084(6)	1.0000	0.055(6)
H2	0.99(5)	0.18(3)	0.041(7)	1.0000	0.055(6)
H3	0.66(4)	-0.04(5)	0.044(7)	1.0000	0.055(6)
H4	0.70(3)	0.56(4)	0.025(6)	1.0000	0.055(6)
H5	0.45(5)	0.77(4)	0.102(7)	1.0000	0.055(6)

Table S3. The bond distances and bond angle list of **K^{Me}Carbamate**.

Bond pairs	Length (Å)	Bond angle triads	Degree (°)
K1^{1} – K1^{2}	4.07466(26)	O1^{1} – K1^{1} – O2^{7}	88.8(5)
K1^{1} – K1^{3}	4.173(8)	K1^{1} – O1^{1} – C1^{1}	105.1(22)
K1^{1} – K1^{4}	4.07466(5)	K1^{6} – O2^{1} – C1^{1}	130.6(18)
K1^{1} – K1^{5}	4.173(8)	O1^{1} – C1^{1} – O2^{1}	134.0(31)
K1^{1} – O1^{1}	2.617(16)	N2^{1} – N1^{1} – C4^{1}	110.8(23)
K1^{1} – O1^{3}	2.793(20)	N1^{1} – N2^{1} – C2^{1}	104.7(25)
K1^{1} – O1^{6}	3.117(17)	N2^{1} – C2^{1} – C3^{1}	111.9(29)
K1^{1} – O2^{5}	2.786(19)	N2^{1} – C2^{1} – C5^{1}	118.6(31)
K1^{1} – O2^{7}	2.559(16)	C3^{1} – C2^{1} – C5^{1}	129(3)
K1^{1} – N2^{6}	2.871(23)	C2^{1} – C3^{1} – C4^{1}	104.7(30)
K1^{1} – C1^{1}	3.134(24)	C2^{1} – C3^{1} – H4^{1}	125(14)
O1^{1} – C1^{1}	1.172(28)	C4^{1} – C3^{1} – H4^{1}	130(12)
O2^{1} – C1^{1}	1.25(4)	N1^{1} – C4^{1} – C3^{1}	107.8(25)
N1^{1} – N2^{1}	1.37(3)	N1^{1} – C4^{1} – H5^{1}	119(16)
N1^{1} – C1^{1}	1.548(23)	C3^{1} – C4^{1} – H5^{1}	133(16)
N1^{1} – C4^{1}	1.38(3)	C2^{1} – C5^{1} – H1^{1}	115(11)
N2^{1} – C2^{1}	1.345(27)	C2^{1} – C5^{1} – H2^{1}	91(11)
C2^{1} – C3^{1}	1.42(4)	C2^{1} – C5^{1} – H3^{1}	115(14)
C2^{1} – C5^{1}	1.48(4)	H1^{1} – C5^{1} – H2^{1}	118(14)
C3^{1} – C4^{1}	1.37(3)	H1^{1} – C5^{1} – H3^{1}	93(13)
C3^{1} – H4^{1}	1.17(16)	H2^{1} – C5^{1} – H3^{1}	127(14)
C4^{1} – H5^{1}	0.91(21)		
C5^{1} – H1^{1}	1.09(19)		
C5^{1} – H2^{1}	1.07(22)		
C5^{1} – H3^{1}	1.06(24)		

Symmetry codes: ^{1} +X,+Y,+Z; ^{2} −1+X,+Y,+Z; ^{3} −X,1/2+Y,1/2−Z; ^{4}1+X,+Y,+Z; ^{5} −X,−1/2+Y,1/2−Z; ^{6} 1−X,1/2+Y,1/2−Z; ^{7} 1−X,−1/2+Y,1/2−Z.

References.

(1) Cho, S.-L.; Liao, C.-J.; Lu, T.-T. Synthetic Methodology for Preparation of Dinitrosyl Iron Complexes. *J. Biol. Inorg. Chem.* **2019**, *24* (4), 495-515. DOI: 10.1007/s00775-019-01668-z.
(2) Hung, M.-C.; Tsai, M.-C.; Lee, G.-H.; Liaw, W.-F. Transformation and Structural Discrimination between the Neutral $\{Fe(NO)_2\}^{10}$ Dinitrosyliron Complexes (DNICs) and the Anionic/Cationic $\{Fe(NO)_2\}^{9}$ DNICs. *Inorg. Chem.* **2006**, *45* (15), 6041-6047.
(3) Sheldrick, G. M.; SADABS. SADABS. *University of Gottingen: Gottingen, Germany* **1996**.
(4) Sheldrick, G. M.; SHELXTL. SHELXTL. *Siemens Analytical X-ray InstrumentsInc.: Madison, WI,* **1994**.
(5) Neese, F. Software Update: The ORCA Program System—Version 6.0. *WIREs Computational Molecular Science* **2025**, *15* (2), e70019. DOI: https://doi.org/10.1002/wcms.70019.
(6) Altomare, A.; Cuocci, C.; Giacovazzo, C.; Moliterni, A.; Rizzi, R.; Corriero, N.; Falcicchio, A. EXPO2013: a kit of tools for phasing crystal structures from powder data. *Journal of Applied Crystallography* **2013**, *46* (4), 1231-1235. DOI: doi:10.1107/S0021889813013113.
(7) Toby, B. H.; Von Dreele, R. B. GSAS-II: the genesis of a modern open-source all purpose crystallography software package. *Journal of Applied Crystallography* **2013**, *46* (2), 544-549. DOI: doi:10.1107/S0021889813003531.
(8) McDonald, T. M.; Mason, J. A.; Kong, X.; Bloch, E. D.; Gygi, D.; Dani, A.; Crocellà, V.; Giordanino, F.; Odoh, S. O.; Drisdell, W. S.; et al. Cooperative Insertion of CO_2 in Diamine-Appended Metal-Organic Frameworks. *Nature* **2015**, *519* (7543), 303-308. DOI: 10.1038/nature14327.
(9) Esfahani, H. J.; Ghaemi, A.; Shahhosseini, S. Improving CO_2 Adsorption Efficiency of an Amine-modified MOF-808 through the Synthesis of its Graphene Oxide Composites. *Sci. Rep.* **2024**, *14* (1), 18871. DOI: 10.1038/s41598-024-69767-9.
(10) Tseng, Y.-T.; Ching, W.-M.; Liaw, W.-F.; Lu, T.-T. Dinitrosyl Iron Complex [K-18-crown-6-ether][$(NO)_2Fe(^{Me}PyrCO_2)$]: Intermediate for Capture and Reduction of Carbon Dioxide. *Angew. Chem. Int. Ed.* **2020**, *59*, 11819-11823.
